# Ocean drones enabling long-term earthquake monitoring in target zones

**DOI:** 10.1038/s41598-025-03250-x

**Published:** 2025-05-30

**Authors:** Diogo Luiz de Oliveira Coelho, Marcelo B. de Bianchi, Ítalo C. B. S. Maurício, Carlos A. M. Chaves, Sergio L. Fontes, Ricardo G. Borges

**Affiliations:** 1 Departamento de Geofísica, Observatório Nacional, Rio de Janeiro, 20921-400 Brazil; 2 Departamento de Geofísica, Instituto de Astronomia, Geofísica e Ciências Atmosféricas, Universidade de São Paulo, São Paulo, 05508-090 Brazil; 3 The Leopoldo Américo Miguez de Mello Research, Development and Innovation Center, Rio de Janeiro, 21941-915 Brazil

**Keywords:** Ocean drones, Marine seismology, Earthquake, Seismology, Geophysics, Physical oceanography

## Abstract

Seismic station coverage in the oceans is limited due to high costs and logistical challenges, leading to insufficient earthquake data from oceanic regions. Ocean drones, with quiet operation, buoyancy-driven mechanics, and autonomous underwater profiling, provide a promising alternative for near-real-time data acquisition. We evaluated an oceanic seismological platform using 6 years (2015–2021) of passive acoustic monitoring data from ocean drones in the Santos Basin, southeastern Brazil, originally not designed for earthquake monitoring. Our analysis identified 12 potential earthquake signals, characterized by low-frequency seismic energy and emergent patterns. These findings demonstrate that ocean gliders are highly effective for earthquake monitoring, offering significant advantages for long-term, targeted seismic observations in coastal and marginal areas where conventional methods often face operational limitations.

## Introduction

The majority of surface of our planet is covered by water ($\approx 70\%$), and, historically, seismic data has predominantly been collected from land areas due to the sparse distribution of seismic stations in the ocean. Seismic monitoring in the oceans has been constrained by the high costs and complexities associated with deploying fixed instruments adapted to the extreme conditions of the ocean floor, such as ocean-bottom seismometers^1,2^, moored hydrophones^3,4^, and distributed optical fiber sensors^5,6^. Most of these experiments suffer with power restrictions^7^, reduced durability^1,8^, recovery issues^1^, failure of the leveling system^9^, effects of the ocean noise^10,11^ and expensive installation and maintenance investments^12^. The data acquisition campaign necessitates a specialized research vessel survey such as a detailed bathymetry information to allow for selecting instrument deployments sites^13^. These requirements significantly restrict the spatial extent and density of deployments. Additionally, the instruments lack the capability to transmit data during deployment, leading to delays of months or even years between data acquisition and the availability of processing results.

**Fig. 1:**
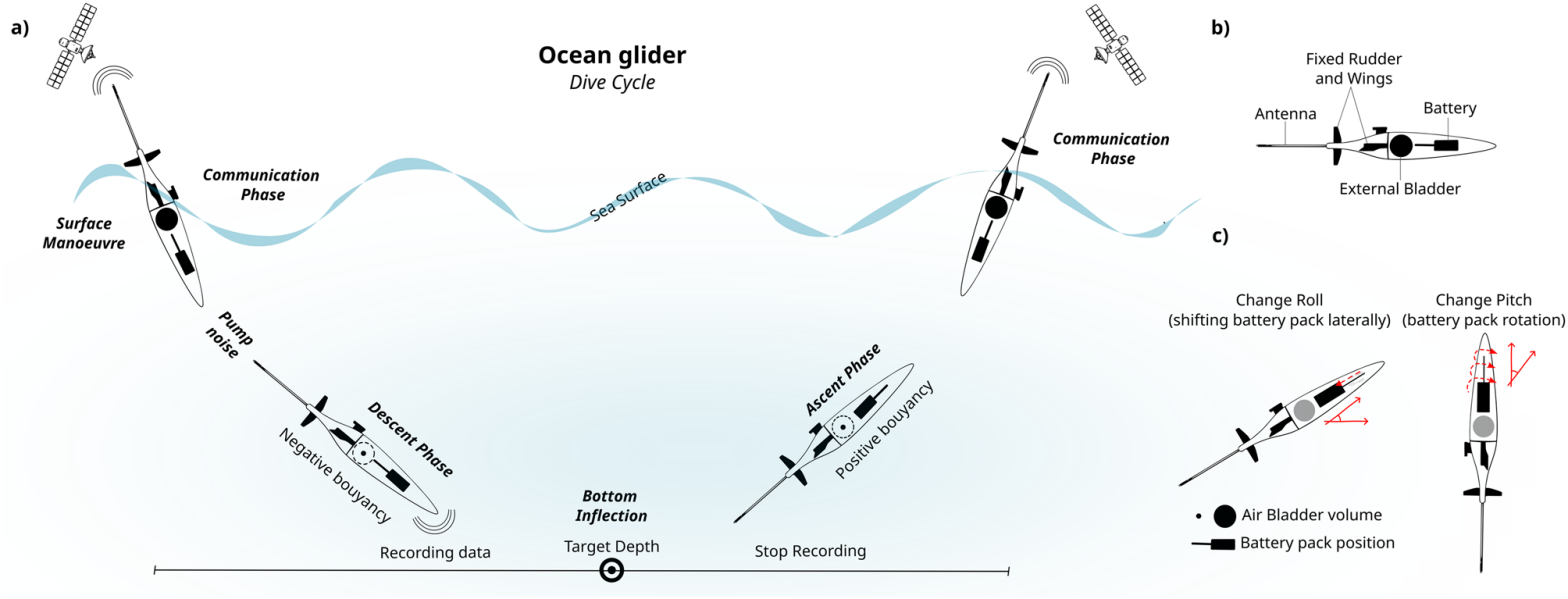
Schematic diagram illustrating the dive cycle of an ocean glider. (**a**) Typical dive profile, showing changes in battery position and bladder volume that control descent and ascent. Data were recorded exclusively during the descent phase. (**b**) Diagram of the glider and its main components. (**c**) The battery pack shifts forward and backward to adjust pitch, and rotates to induce roll, enabling the glider to turn.

Traditional drifting sensors, such as ARGO floats, acronym for Array for Real-time Geostrophic Oceanography^14^, and MERMAID floats, acronym for Mobile Earthquake Recording in Marine Areas by Independent Divers^15^, can rapidly cover large areas and provide near-real-time water column sampling, thereby reducing the data gap in oceanic areas^16^. These profiling instruments offer a cost-effective solution for recording hydroacoustic data from the oceans in near-real-time, eliminating the need for deployment and recovery by large research vessels. They are capable of continuous operation for years without requiring retrieval. Over the past decade, there has been a significant increase in the use of autonomous vehicles in marine environments, particularly ocean drones equipped with passive acoustic monitoring (PAM) systems^17^. Ocean drones, hereafter referred to as ocean gliders, are capable of being piloted from office along desired routes, controlled by pumping oil in and out of an external bladder or by using a piston, inducing a vertical motion in the water column between the surface and a specified depth^18,19^. Furthermore, the battery packs can be rotated to perform heading adjustment^20^. The entire glider cycle is illustrated in Fig. 1. Ocean gliders are equipped with a plethora of sensors, and can be used to detect natural and anthropogenic sounds in marine environments, including the study of marine mammal behavior^21^, anthropogenic exploration and production activities^22^, real-time marine forecasting^23^, integrated offshore seismic survey^24^, autonomous ocean-bottom nodes^25^, vertical seafloor geodesy^26^, and monitoring of underwater activity^19^. Unlike traditional profiling floats, ocean gliders offer greater controllability and flexibility, making them an optimal choice for coastal regions or marginal seas^23^. Recent studies have investigated the detection and classification of signals generated by seismic events, such as the earthquake that occurred on November 15, 2017, in Pohang, South Korea^18^, and the eruptive activity of the submarine West Mata volcano^27^. These studies have ignited our interest in exploring the hydroacoustic wave propagation in an ocean utilizing autonomous underwater vehicles.

**Fig. 2:**
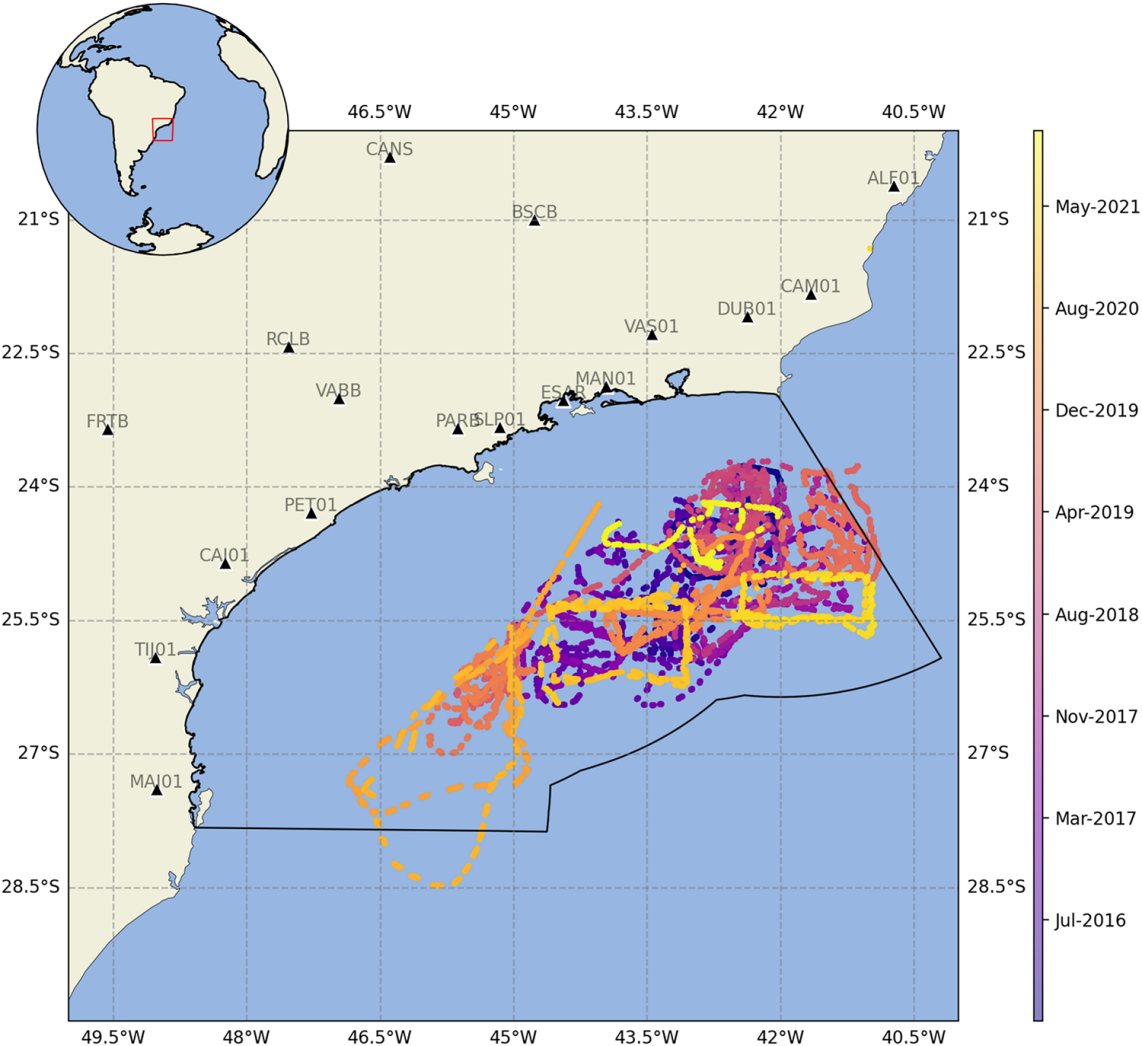
The trajectories of PMPAS-BS gliders over 6 years of monitoring. Locations of dives conducted between November 2015 and November 2021. Black triangles indicate the stations of the Brazilian Seismographic Network (RSBR). The black solid line represents the geographical boundaries of the Santos Basin. Generated using Cartopy (https://scitools.org.uk/cartopy/), a Python library for geospatial visualization.

The aim of this study is to evaluate the effectiveness of autonomous underwater vehicles in earthquake monitoring. Figure 2 presents the trajectories of 52 ocean glider missions conducted between November 2015 and November 2021 as part of the Santos Basin underwater soundscape monitoring Project (PMPAS-BS)^22^. The primary challenge of our project is repurposing an experiment not originally designed for seismic monitoring, which involves dealing with short daily recording windows, suboptimal sensor configurations, and several sources of internal and external noise. We utilize data from cataloged seismic events, including those from the Global Centroid-Moment-Tensor (CMT) Project for global earthquakes^28^ and the Bulletin of the Brazilian Catalog (SISBRA) for local earthquakes. This study presents data on both local and teleseismic earthquakes and compares it with data from the Brazilian Seismographic Network (RSBR) located near the southeast coast of Brazil. Our findings showcase the potential of ocean gliders to monitor earthquakes in marginal oceanic areas, contributing to our understanding of offshore and global seismicity.

**Fig. 3:**
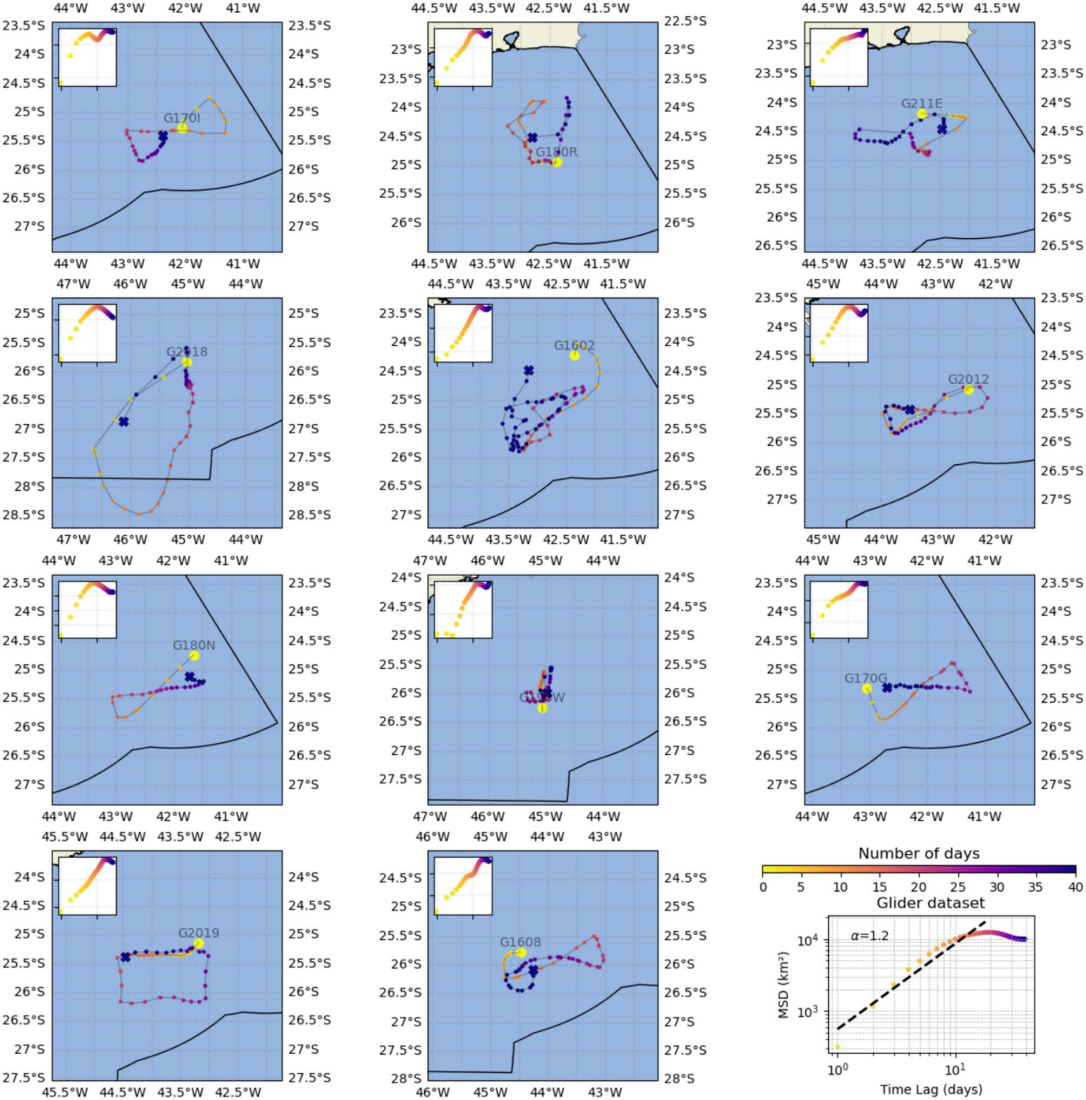
Plot of the 11 glider trajectories in the Santos Basin. Each panel shows the mean squared displacement in the top-left inset. The last panel presents the mean squared displacement (MSD) of the entire dataset versus time on a log–log scale, with the estimated fit shown as a dashed black line. The diffusion exponent ($\alpha$) is presented. Colors represent the number of days, and the initial and final positions are marked by a large circle and cross, respectively. Time is measured in days, and dispersion is in $\text {km}^{2}$.

## Results

Our non-continuous dataset from the PMPAS-BS spans from November 2015 to November 2021. The completeness of the single ocean glider in operation are illustrated in Fig. S1. While surfacing, the ocean gliders cease recording due to the high self-noise generated during the maneuver, resulting in a temporary absence of data during this period. Furthermore, the average length of hydroacoustic daily data is only 6 h, representing approximately 2 dives per day, which is the minimal request as mandated by government regulations. It is significant to mention that the original design of this experiment did not specifically account for earthquake monitoring, as indicated by the sensitivity levels of the hydrophone models (Fig. S2). Consequently, the recorded signals are anticipated to have a low signal-to-noise ratio because of the high-frequency characteristics of the hydrophone models. The results section comprises teleseismic earthquakes extracted from the Global Centroid-Moment-Tensor (CMT) Project online catalog and local earthquakes identified through the Bulletin of the Brazilian Catalog (SISBRA).

### Global earthquakes

**Fig. 4:**
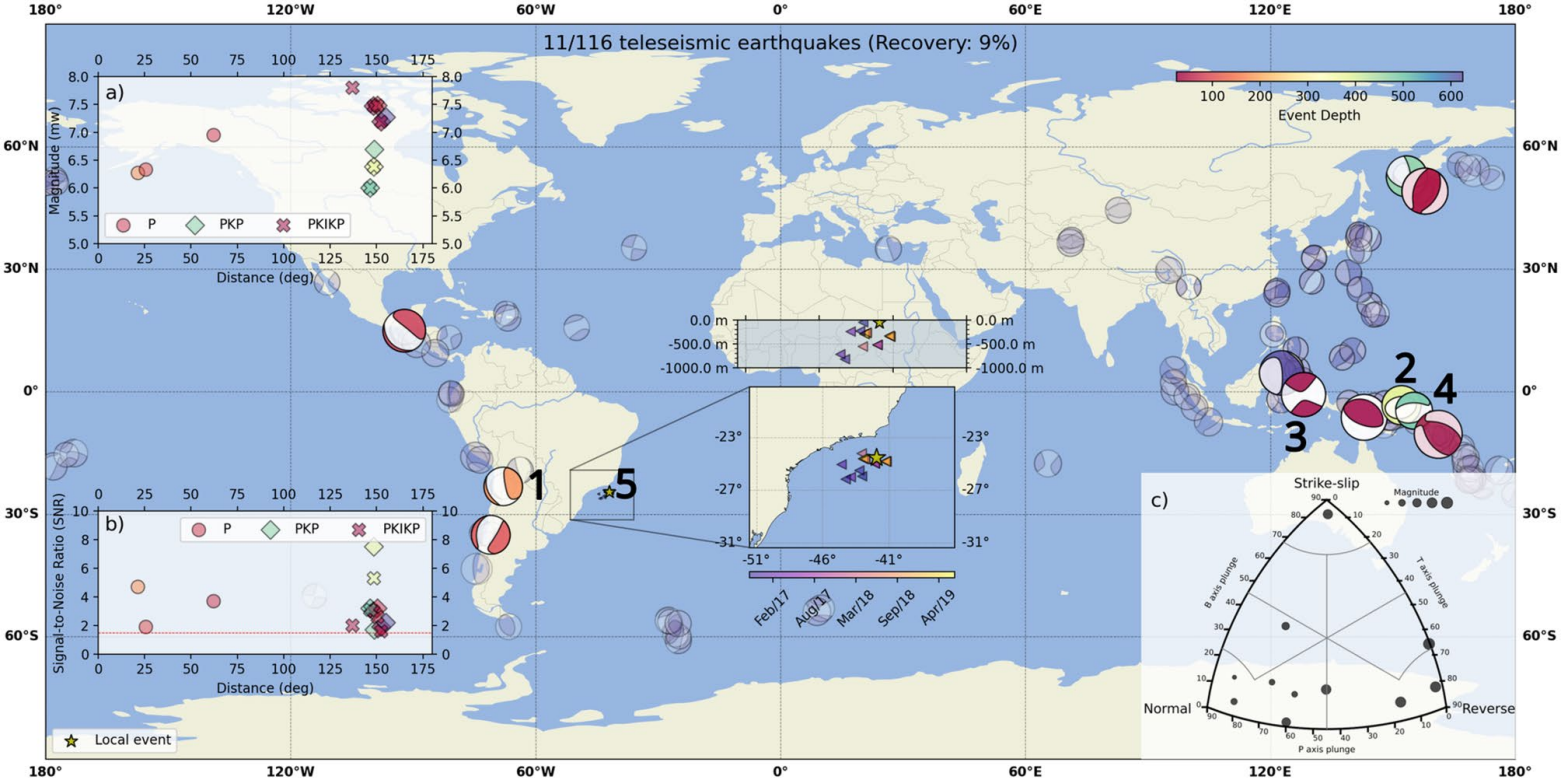
Analysis of the earthquakes detected during the ocean gliders missions. The epicentral locations of the 11 earthquakes are illustrated, indicating both the depth and focal mechanisms of each event. Notably, the central insets highlight the specific locations of the ocean gliders at the moment of registering the P-wave arrival from the teleseismic event. (**a**) Distribution of epicentral distances and magnitudes, highlighting the depth of each event. (**b**) Distribution of epicentral distances and signal-to-noise ratios (SNR). (**c**) Focal mechanisms classification^56^ into three main areas: normal, reverse and strike-slip. Generated using Cartopy (https://scitools.org.uk/cartopy/), a Python library for geospatial visualization.

Due to anthropogenic, environmental, or instrumental factors, noise is inevitably introduced during the glider data acquisition phase. This noise can lead to false event detection, making the identification of seismic phases amidst the noise a significant challenge. Our earthquake detection approach, which requires minimal intervention, utilizes spectral analysis based on the estimated theoretical phase arrival times of seismic body waves. Within a window centered on the arrival of the event, we analyze changes in amplitude and signal-to-noise ratio (SNR) to automatically classify potential earthquakes signals (see Methods). The effectiveness of the earthquake detection results is assessed by the ratio of detected earthquakes to the potential waveforms from seismic events with a magnitude higher than 6 that occurred around the world. To evaluate the maximum potential of the gliders in detecting signals from global earthquakes, we applied two thresholds based on the epicentral distance ($\Delta$), defined as the distance between the earthquake’s epicenter and the seismic station: one for direct P waves up to 100 degrees, and another for PKP and PKIKP arrivals, which are compressional P waves that traverse the Earth’s interior, outer core, and inner core, respectively. From a raw dataset of 781 unique earthquakes ($magnitude \ge 6.0$) between November 2015 and November 2021, only 116 unique earthquake waveforms were extracted. Of these 116 potential events ($21^{\circ } \le \Delta \le 175^{\circ }$), which can be observed in the Fig. S7, only 14 unique seismic events were detected automatically: 4 related to mantle phases (P), and 10 to core-transmitted phases (PKP and PKIKP). Upon further visual inspection, it was determined that 3 of the 14 automatically detected events lacked a distinct time-frequency signature characteristic of an earthquake, such as a sharp onset (noting that background noise can sometimes obscure a steep onset) and high spectral energy at low frequencies. As a result, this left 3 unique earthquake detections related to P phases and 8 to PKP and PKIKP phases. The rejected signals were consequently reclassified as false triggers, which account for $\sim 21\%$ of our detections. This reclassification underscores the importance of thorough examination in ensuring the accuracy of earthquake detection, particularly given the hydrophone sensitivity and the primary purpose of the ocean glider, which is not for earthquake monitoring. These reported events exhibited reasonable time-domain signal-to-noise ratios ranging from 1.6 to 7.5 in the 2.5–5 Hz and 0.7–2 Hz bands. Additionally, the STA/LTA (Short-Time Average with a 2-second window, and Long-Time Average with a 30-second window) maximum values ranged from 3.5 to 11.4. Table S1 summarizes the regional and teleseismic earthquakes, which exhibit similar spectral characteristics and good time-domain signal-to-noise ratio in the 0.07–5 Hz range. As depicted in Fig. 4, the ocean glider recorded P waves from distances ranging between $21.4^{\circ }$ and $62.2^{\circ }$, and 8 arrivals of phases that transited the outter and inner core of the Earth (restricted to the neighborhood of $148^{\circ }$), with magnitudes ranging between 6 and 7.8 Mw and hypocentral-depths (*H*) estimates varying between 12 and 621.5 km. The Fig. 4 also shows that ocean gliders primarily recorded earthquakes occurring along the subduction zones of the Pacific Rim.

**Fig. 5:**
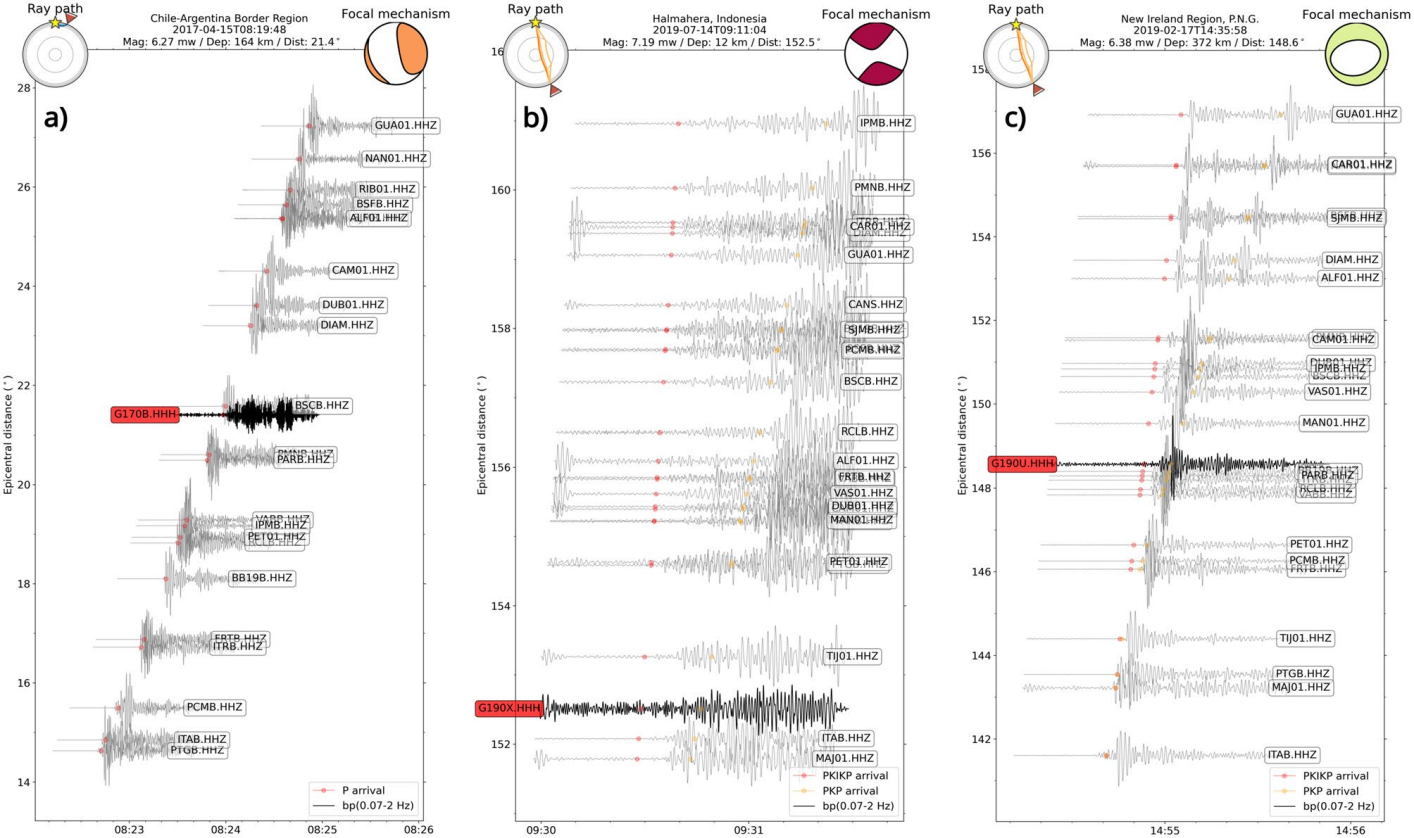
Waveform comparison of intermediate-focus, deep-focus, and shallow-focus earthquakes from ocean gliders and RSBR stations for earthquakes 1, 2, and 3, respectively, as identified in Fig. 4. The waveforms were normalized and filtered using a bandpass filter between 0.7 and 2 Hz. Land stations are represented by grey lines, and ocean gliders by black lines. (**a**) Chile-Argentina border earthquake with a magnitude of 6.27 Mw, which occurred on April 15, 2017, at 08:19:43 UTC. (**b**) New Ireland region earthquake with a magnitude of 6.38 Mw, which occurred on February 17, 2019, at 14:35:55 UTC. (**c**) Indonesian earthquake with a magnitude of 7.19 Mw, which occurred on July 14, 2019, at 09:10:51 UTC. The circles indicate the theoretical arrival times calculated using the ak135 Earth model^57^. The top insets of the figure display the ray paths (left) and the focal mechanism (right).

The expected delay time in P-wave arrival times resulting from the acoustic conversion of seismic phases at the seafloor interface and their propagation to the ocean glider through the water column was estimated using a modification of the ak135f Earth model^29^, roughly adapted to our offshore geological context (Fig. S3). The Fig. 5a displays the normalized waveforms of the nearest teleseismic P-wave arrivals from 6.27 Mw Chile-Argentina border earthquake (ID:201704150819A), which occurred at a depth of 164 km on 15 April 2017 at 08:19:43 UTC, along with vertical component seismograms of nearby coastal stations (grey lines) from RSBR, as well as the earthquake’s ray path and focal mechanism. The P-wave arrival times for the land stations were estimated using the ak135 Earth model, and the waveforms were trimmed to 30 s before and 60 s after the theoretical arrival, revealing clear delay times across the stations relative to their distance. The Fig. S8 displays the spectral analysis of the signal recorded from the ocean glider, where the P-wave arrival is confined to a relatively narrow band (0.7–5 Hz) and exhibits high SNR and STA/LTA values of 4.7 and 8.3, respectively. This indicates a clear emergent signal observed near the theoretical arrival time, with estimated residual times of approximately -3 s.

**Fig. 6:**
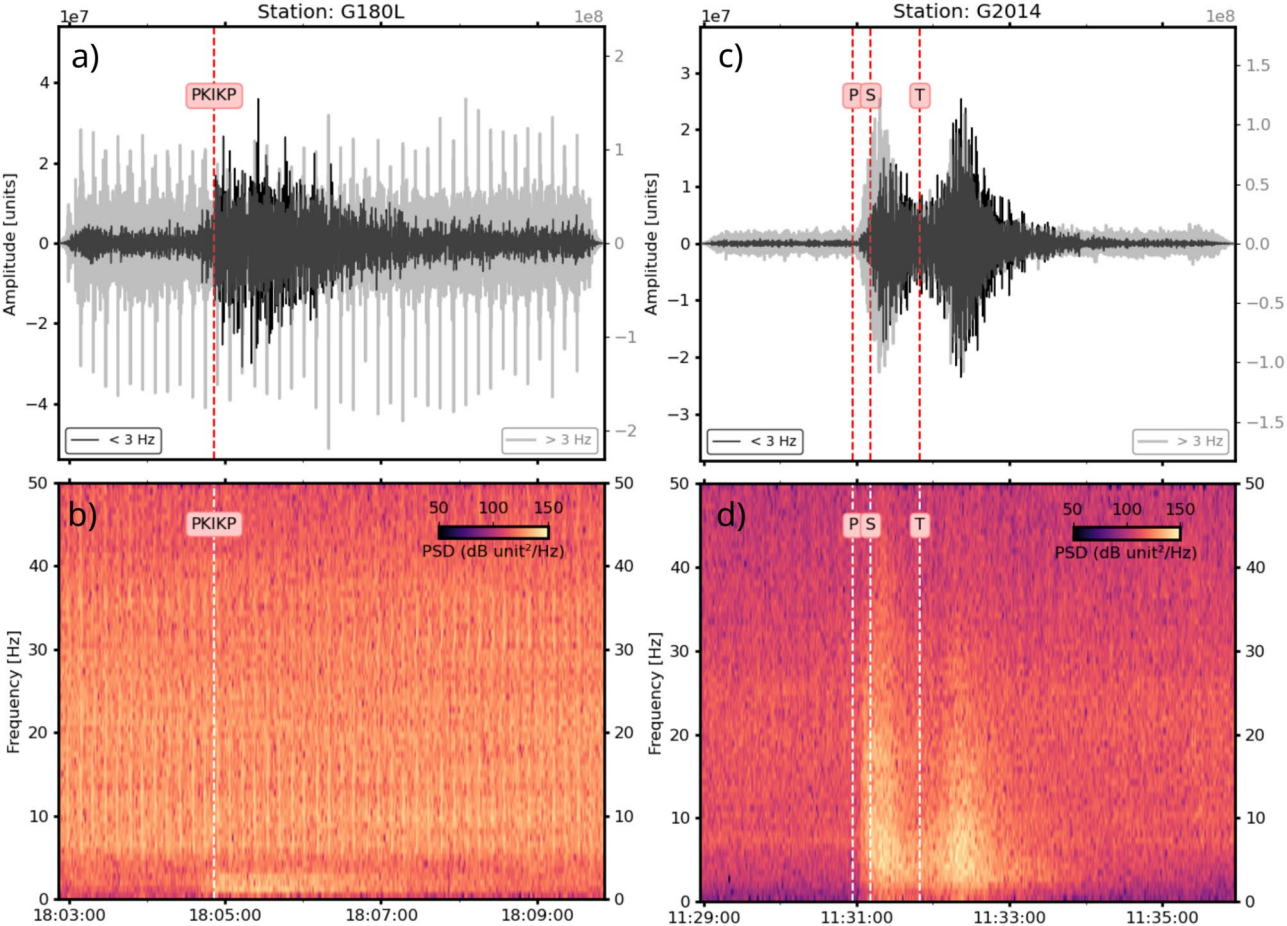
Waveforms, spectrograms, time series windows centered on the theoretical arrival times, and STA/LTA ratio curves for events 4 and 5. (**a**) Earthquake with a magnitude of 6.0 Mw, which occurred on July 11, 2019, at 17:08:38. The vertical dashed lines (red/white) and the letter PKP indicate the theoretical arrival time calculated using the modified ak135f. (**b**) Local earthquake of magnitude 3.7 mb, which occurred on March 25, 2020, at 11:30:39. The vertical dashed lines (red/white) and the letters P, S, and T indicate the theoretical arrival times calculated using the ak135f (Fig. S41) for P-wave, S-wave and hydroacoustic phases, respectively. The ray paths of these local phases are depicted in Fig. S4. The black and grey lines are the waveforms filtered below and above 20 Hz, respectively. The red, blue and orange horizontal lines represent the preset lower and upper limits for the STA/LTA ratio trigger. The insets at the top of the figure show the global map and the ray paths of a given seismic phase. The waveforms are presented in digital units.

The predominance of intermediate ($70< H < 350 \text {km}$) and deep-focus (H $> 350 \text {km}$) earthquakes detected by the ocean glider is evident, with 7 out of the 11 detected earthquakes occurring within these depth intervals. These seismic events present high SNR and STA/LTA values, considering our specific case, ranging from 1.6 to 7.5 and 3.5 to 11.4, respectively, even with earthquakes with magnitude near to 6 Mw. Larger magnitude earthquakes, which release more energy, tend to produce higher amplitude signals that are easier to detect, as observed in the PKP and PKIKP phases detected, most earthquakes with magnitude higher than $\approx 7$ Mw were automatically detected after distances higher than $137^{\circ }$. Despite their high magnitudes, these events exhibit lower SNR compared to other recorded events, with SNR values ranging from 1.6 to 3.2 and STA/LTA maxima between 3.5 and 6.8. This is illustrated in Fig. 5b with the shallow-focus ($H=12 \text {km}$) magnitude 7.19 Mw strike-slip earthquake (ID: 201907140910A) that occurred in the Halmahera region of Indonesia on July 14, 2019, at 09:10:51 UTC. Even deep-focus earthquakes with dip-slip focal mechanisms and magnitude 7 exhibit low SNR and STA/LTA near the first arrival time, as shown in Figs. S7–S13 in the supplementary material. The Fig. 5c presents an unexpected example of low magnitude and high SNR and LTA/STA values with the magnitude 6.4 Mw earthquake (ID:201902171435A) that occurred at the New Ireland region (Papua New Guinea) on February 17, 2019, at 14:35:58 UTC, at 372 km depth and approximately 16,500  km away (Dist: $148.5^{\circ }$). Similarly, the Fig. 6a and b depicts a magnitude 6.0 Mw earthquake (ID:201907111708A) occurred at the Solomon Islands (northeast of Australia) at July 11, 2019, at 17:08:39 UTC at 493 km depth approximately 16,000 km away (Dist: $146.5^{\circ }$), presenting values of SNR and STA/LTA of 3.2 and 9.3, respectively. This event exhibited values of SNR and STA/LTA of 7.5 and 11.4, respectively, enabling clear observation of the arrival of the outer (orange circle) and inner (red circle) core phases, in comparison with coastal inland stations from the RSBR. The paths of each seismic phase are shown in the upper left inset of the figure, while the focal mechanism of the fault is displayed in the upper right inset. A comparison of records from ocean gliders with those from inland seismic stations in the same region shows similar signal characteristics. Notably, the ocean glider data demonstrate a pronounced dominance of PKP phase amplitudes in the detection process. This is illustrated in Figs. 5a and 6a, and in the supplementary material, specifically in Figs. S13–S24.

### Local earthquakes

Our analysis focused on local and nearby regional earthquakes using the SISBRA seismic bulletin, published by the Seismology Center at São Paulo University^30^. This bulletin is based on a re-analysis of cataloged seismic events and offers a tabulated compilation of information, including coordinates, origin times, and magnitudes of earthquakes primarily detected and analyzed by the Seismology Center in Brazil. We employed the same methodology previously used for detecting nearby earthquakes. However, local events (with epicentral distances less than 100 km) pose a challenge because their energy includes both low frequencies and high frequencies. This variation in frequency content impedes the detection of local events using the methodology that was effective for detecting regional and teleseismic events. Therefore, we used the signatures of regional events as a basis for identifying local events, visually inspecting the results of our analysis without the final classification step. Out of 142 events listed in the SISBRA catalog along the coast, only one was classified as an earthquake. In Fig. 6b (complete version in Fig. S41), the clear energetic arrival of P, S, and T waves from a local 3.7 mR earthquake, which occurred on 2020-03-25 at 11:30:39, is shown. Theoretical travel times estimated from the velocity model are shown by the red and white curves, while the corresponding ray paths are depicted in Fig. S4. The estimated residual time was -5.6 s, indicating a reasonable travel-time estimation given the approximate location of our ocean glider. The STA/LTA curve displayed a steep onset within the frequency band of 2–5 Hz, while the centered theoretical P-wave time series depicted an emergent signals with an SNR of 1.5. However, the energy observed in the spectrogram from the P and S waves exhibited emergent signals across the entire frequency range ($0.7 \text {Hz}< f < 50 \text {Hz}$). Furthermore, approximately 1 min later (reflecting the wave propagation speed at water column $\approx 1.5\,\text {km}/\text {s}$), we observe a typical T wave characterized by its emergent signal, spindle-shaped, long-duration ($> 60\,s$), and predominant frequency energy between 1 and 10 Hz. This seismic event was effectively detected for several reasons: (1) high magnitude, (2) low background noise, (3) short epicentral distance, and most importantly, (4) the event occurred during the glider dive.

## Discussion

The primary discovery derived from this analysis is the observed relationship between detection rates and factors such as magnitude, epicentral distance, and focal depth, as is commonly observed in other similar studies^3,31–33^. Our observations suggest that the primary factor influencing the detection of earthquakes using hydroacoustic datasets is focal depth. This factor is closely related to the propagation paths, which can vary in efficiency based on the observed amplitude and frequency content. Specifically, 7 out of 11 detections were associated with intermediate and deep-focus earthquakes, highlighting the impact of this parameter on detection accuracy. The amplitude and frequency content of the observed P, PKP, and PKIKP phases changed significantly with event depth. A high attenuation zone in the upper mantle^34,35^ promotes the decay of seismic wave amplitude and energy, particularly at high frequencies. Consequently, the frequency content of shallow-focus earthquakes is predominantly around 1 Hz, while deep-focus earthquakes exhibit predominant frequencies of approximately 5 Hz, as confirmed by their high SNR and STA/LTA values across different frequency bands.

Regarding the amplitudes of seismic waves that traveled through the core, our observations, consistent with other hydroacoustic studies^3,33^, indicate that the magnitude contributes to high-amplitude arrivals. Additionally, our findings and results from MERMAID data^31^ demonstrate that amplitudes from outer core phases are higher than those from inner core phases. This discrepancy is attributed to the lower attenuation effect of the outer core compared to the inner core, which is due to significant bulk attenuation in the inner core^36^. Anomalously high values of SNR and STA/LTA ratois for PKP phases from earthquakes with magnitudes around 6 from distant locations can be explained by the caustic effect produced by the outer core^37^. The large amplitudes are concentrated at epicentral distances around $145^{\circ }$^3,37–40^. Analysis of earthquake focal mechanisms and recovery rates reveals a predominance of mechanisms associated with dip-slip events(see Fig. 4D). However, the signal-to-noise ratio (SNR) and STA/LTA ratio values show no significant differences between dip-slip and strike-slip earthquakes. Given the limited scope of our sample, additional data are needed to validate these observations.

Our automatic signal identification utilizes differences in spectral properties to detect earthquake signals and reject noise and false triggers. Our results showed a relatively small percentage of false triggers, approximately 20%, compared to other studies using hydrophones^41^, despite our suboptimal hydrophone configuration. Examining high-frequency waveforms and the spectrograms, we detected signals indicative of seismic surveys. Typically, these airgun arrays produce high-intensity, broadband (10–500 Hz) that are impulsive over extended periods^42^, as illustrated in Fig. 6a and b. In the case of the Southeast coast of Brazil, a prolific oil and gas region, seismic activities can persist for months^22^, with the energy emitted detectable at considerable distances. Even surrounded by noisy medium, the earthquakes signals were clear detected. These results demonstrate the efficacy of ocean gliders, in detecting and classifying seismic events on both local and global scales, as previous shown for other mobile independent divers^15,33^. This remains valid even under severe background noise levels.

## Conclusion

Our work represents a significant advancement in seismic monitoring of natural and anthropogenic sources in predefined areas using ocean gliders. We analyzed 6 years of data from the Santos Basin Underwater Soundscape Monitoring Project, which included 52 ocean glider missions. Our analysis identified at least 11 global and 1 local earthquakes from international catalogs. The recognition rate for global earthquakes with magnitudes above 6.0, according to the GCMT catalog, is approximately 9%, with epicentral distances ranging from 21 to 155 degrees. Our results demonstrate a commendable automatic earthquake recognition rate of approximately 20%. It is important to note that the equipment was not initially designed for earthquake monitoring, which involved managing short recordings and various sources of internal and external noise. These oceanographic mobile platforms show promising capabilities due to their ability to integrate various sensors for multidisciplinary monitoring and their near-real-time communication via Iridium satellite connections. Additionally, acquisition campaigns can be tailored to enhance seismic monitoring by directing gliders to specific waypoints based on predetermined patterns^19^. Moreover, their versatility extends to exploring extraterrestrial environments^43^, thanks to their autonomous operation in challenging environments, requiring minimal power and intervention over extended periods.

## Materials and methods

### PMPAS-BS description

The Santos Basin Underwater Soundscape Monitoring Project (PMPAS-BS in Portuguese acronym) is an unprecedented passive underwater acoustic measurements in the southwestern South Atlantic. It utilizes a variety of technologies, including ocean gliders, drifting profilers, coastal submarine observatories, and instrumented mooring lines, more details in^22^. Our dataset comprises 52 glider campaigns conducted between 2015 and 2021 (Fig. 2). Due to satellite restrictions on high-volume data traffic (e.g., raw 128 kHz WAV files), the data could not be transmitted and remained stored in the equipment. It was recovered at the end of the mission, which occurred every 30–60 days. The acoustic recordings are not continuous but rather defined by windows: in the gliders, there are basically 3 h of data per dive, with a minimum of 2 dives per day, totaling 6 h per day. The typical seismological MSEED files were decoded from the standard raw WAV audio file format. The data set were reduced from a 128 kHz to 100 Hz sampling rate for earthquake occurrence analyses, as well as, time-aligned and metadata management. The names of the stations, previously mentioned, were schematized in function of the data acquisition campaign, as follows: G+year+hexatrigesimal two-digit algorithm conversion according to the campaign that occurred in the year.

### Three-dimensional sampling layouts

Ocean gliders execute successive dives along pre-defined trajectories, utilizing satellite communication while at the sea surface to transmit data to servers and update their mission plans as necessary. They are propelled by buoyancy changes, achieved by pumping oil in and out of an external bladder, which induces V-shaped ascent and descent profiles in the water column between the sea surface and a target depth, at preset speed of $\approx 13\, \text {cm}/\text {s}$. Direction is determined either by rotating the internal battery or by employing a rudder, depending on the glider type, while underwater navigation relies on the use of a compass (Fig. 1). Ocean gliders generate noise during maneuvers, such as the noise produced by the buoyancy pump, which emits a broadband signal covering the entire recorded spectrum, 1–62 kHz, potentially masking the collected data^19^. The glider dive cycle occurs every 6 h, with GPS location data transmitted during the surface phase of each survey. The underwater trajectory is determined assuming a linear trajectory^44,45^ between successive surfaced GPS points and measurements from the Conductivity, Temperature, Depth (CTD) sensor. Due to satellite data limits, raw acoustic data were stored onboard and retrieved during glider recovery every 30–60 days^22^. Ocean glider pilots typically aim to minimize surface time by reducing the volume of transmitted data. This precaution is especially crucial in coastal ocean regions, which are inherently riskier due to potential collisions with ships and strong currents, resulting in a heightened workload for glider pilots^23^.

Ocean gliders offer unparalleled opportunities for long-term and spatially extensive monitoring. The controlled, adaptable nature of ocean glider missions contrasts sharply with the passive drift of ARGO floats. Unlike current-driven sampling patterns employed by typical floats, glider missions follow purposefully designed trajectories, providing greater control over data collection and enabling targeted observations. Argo floats, as well as MERMAIDs floats, primarily drift with ocean currents and collect data as they move between the surface and a mid-water level. While this approach is important for large-scale monitoring of oceanographic parameters, such as temperature and salinity, its spatial coverage is dictated by ocean circulation, lacking precision and repeatability. In contrast, ocean gliders can execute carefully programmed missions that align with expected variability in the target processes. For instance, repetitive patterns likecross-slope transects or butterfly loops enabling systematic spatial coverage and offering high temporal resolution data of an area of interest^19^.

The movements of the floats are highly coherent and primarily determined by ocean circulation^46^. Along the southeastern continental margin of Brazil, the Brazil Current (BC) flows southward along the South American coastline and is characterized by eddy-dominated dynamics, with the recurrent formation of vortex and meanders^47^. To evaluate the navigational capabilities of ocean gliders, we compared absolute dispersion-defined as the mean squared displacement (MSD) of individual particles from their initial positions- between ARGOs trajectories and gliders in the region. The drifter data for the region were obtained through the ARGO program (https://argo.ucsd.edu/). For the glider data, since most of the 52 acquisition campaigns lasted less than 30 days on average, we opted to use missions lasting more than 40 days, totaling 11 missions. The longer the mission duration, the more divergent the trajectories between float and glider data, as the navigability becomes more evident.

The MSD behavior facilitates the categorization of different types of dynamics based on its relationship with time, often expressed as a power law ($MSD \propto t^{\alpha }$) with linear growth over time. Here, $\alpha$ represents the diffusion exponent, helping to distinguish diffusive behaviors and providing insights into the underlying physical processes. Figure 3 illustrates 11 glider trajectories in the region, along with their respective MSD estimates. These trajectories exhibit common sampling patterns, such as circular, square, and butterfly shapes. The average MSD of the glider trajectories deviates from linear behavior, indicating complex dynamics likely influenced by oriented navigation. In contrast, Fig. S5 presents 11 ARGO trajectories in the area, which show an average linear MSD behavior with an $\alpha$ slightly greater than 1, indicative of superdiffusion, where particles spread faster than in normal diffusion. This behavior aligns with the poleward path of the BC. Overall, these findings highlight the differences between the data acquisition systems of drifters and gliders, emphasizing the gliders’ ability to perform sustained, long-term observations along predefined and carefully planned missions.

### Earthquake signal detection

The earthquake signals detected were analyzed using a detection scheme inspired by the automated preliminary matching^31^ and the automatic discrimination of underwater acoustic signals algorithm^48^. Our simplified version allows the extraction of spectral information from a signal and provides an efficient filter for false triggers for signals from epicentral distances greater than 100 km. Centered on the theoretical phase-arrival times associated with global cataloged earthquakes, our approach incorporates a spectral analysis of five bandpass filters covering the upper half of the original frequency range, 25–50 Hz; the second filter corresponds to 10–25 Hz, and so on: 5–10 Hz, 2–5 Hz, 0.7–2 Hz. The five filters cover a sufficient number of frequency ranges to exploit the differences in the spectra of the expected signals. Typically, bandpass filters are designed to attenuate all signals outside the specified band, allowing for a detailed examination of the spectral content to discriminate between earthquake signals, cruise ships, airgun shots, and the instrument’s self-noise. It is standard practice to use a cosine taper to smoothly set the data to zero at the boundaries before performing the band-pass filter^31^. Additionally, the algorithm integrates the recursive STA/LTA method^49^, which measures the instantaneous amplitude of the seismic signal by comparing the average absolute amplitude in two consecutive moving-time windows: the short-time window (STA), which is sensitive to seismic events, and the long-time window (LTA), which provides information about background noise amplitude. When the seismic signal arrives, the STA/LTA changes rapidly, causing the corresponding STA/LTA value to increase significantly.

By combining these two methods, the hybrid algorithm computes the signal-to-noise ratio (SNR) and the maximum value of the STA/LTA curve for each frequency band within a time window of $\pm 10$ s around the theoretical arrival time as a recognition criterion. If the signal presents $STA/LTA \ge 3$ and $SNR \ge 1.5$ in the last two frequency bands and, no signal appears jointly in the other bands, the signal is marked as an earthquake; otherwise, it is marked as noise. This method enhances the detection and analysis of seismic signals, improving the accuracy and reliability of identifying the time of arrival of true global earthquakes based on the analysis of the relative power distribution among different frequency bands, as can be observed in the panels of the Fig. S8. As the southeast coast of Brazil is dominated by offshore operations, elevating the amplitude of irregular man-made seismic noise and decreasing effectiveness of the trigger methods, as well as the self-noise affect the effectiveness of this type of trigger. Therefore, this hybrid procedure is fundamental to improving the automatic detection of seismic events.

## Supplementary Information

Supplementary Information.

## Data Availability

The dataset originates from the Santos Basin Underwater Soundscape Monitoring Project, with the primary goal of quantifying and evaluating anthropogenic hydroacoustic noise associated with the exploration and production (E&P) activities in the offshore region of southeastern Brazil. The datasets generated during the current study are available in the Zenodo repository^53^, https://doi.org/10.5281/zenodo.14559493. We used data from Seismographic Network of South and SouthEast Brazil^54^(10.7914/SN/ON).

## Code availability

The implementation codes are available online^55^ at https://github.com/dIOGOLOC/Codes_Ocean_drones_monitoring_earthquakes_in_ultradeep_waters.
