# Supplementary material for: Ocean drones enabling long-term earthquake monitoring in target zones
Source: Sci Rep. 2025 May 30;15:19089. doi: 10.1038/s41598-025-03250-x (PMC12125381; doi:10.1038/s41598-025-03250-x)
Supplement: Supplementary file 1 — Supplementary Information. [file 41598_2025_3250_MOESM1_ESM.pdf]

Scientific Reports
Supporting Information for

Ocean drones enabling long-term earthquake monitoring in target zones

Diogo L. de Oliveira Coelho[1], Marcelo B. de Bianchi[2], Ítalo C.B.S. Maurício[1], Carlos A. M. Chaves[2], Sergio L. Fontes[1], Ricardo G. Borges[3]

1. Geophysics Department, Observatório Nacional, Rio de Janeiro, Brazil
2. Geophysics Department, Universidade de São Paulo, São Paulo, Brazil
3. The Leopoldo Américo Miguez de Mello Research, Development and Innovation Center, Rio de Janeiro, Brazil

Contents of this file

Figures S1 to S41
Table S1

This supplement provides additional examples of real data that were collected and analyzed. It includes: data completeness, hydrophone frequency response, the Earth model and ray paths used in this study, ARGOs trajectories analisys, and the schematic of the automatic event detection algorithm and spectral analysis results. Additionally, further results of automatically detected global earthquakes and detection performance between glider and inland stations; and an example of local event.

Introduction

Data Completeness

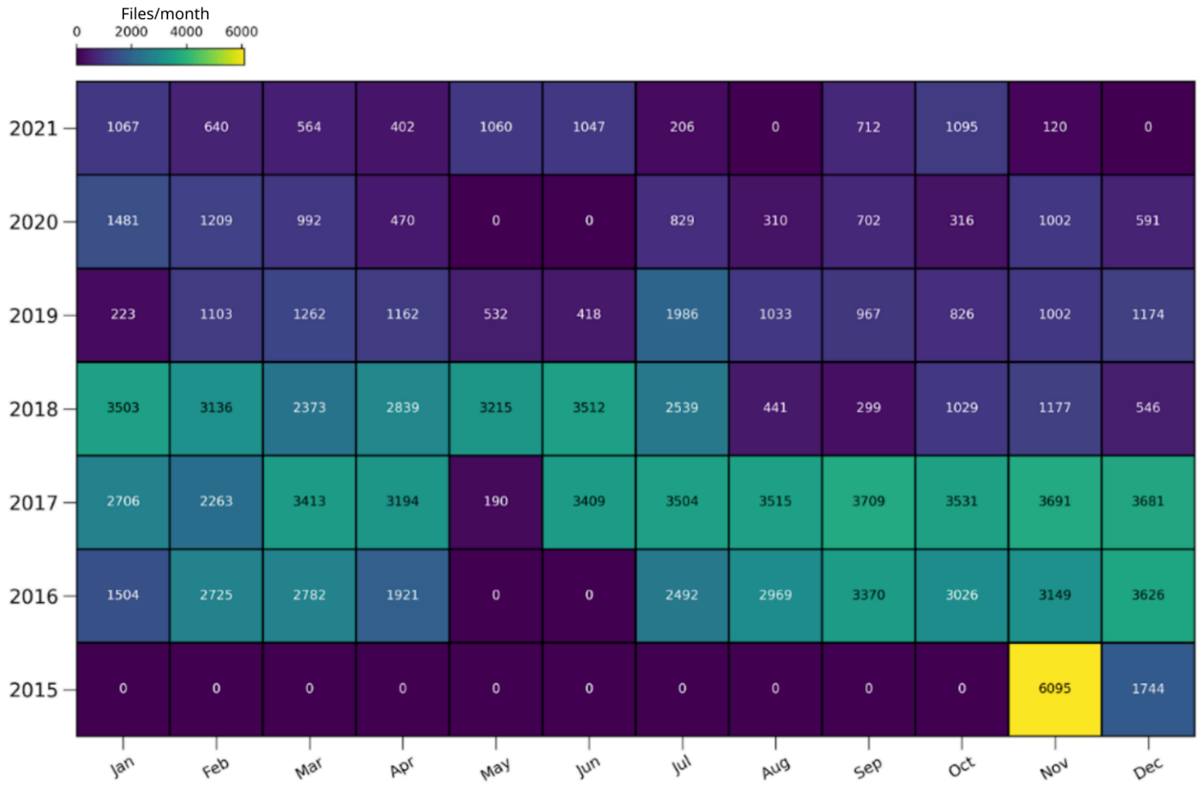

Figure S1: Completeness of ocean glider data. Completeness of ocean glider data resampled from audio files (.wav) to the format adopted in Seismology (.mseed).

Materials and Methods

Hydrophone frequency response

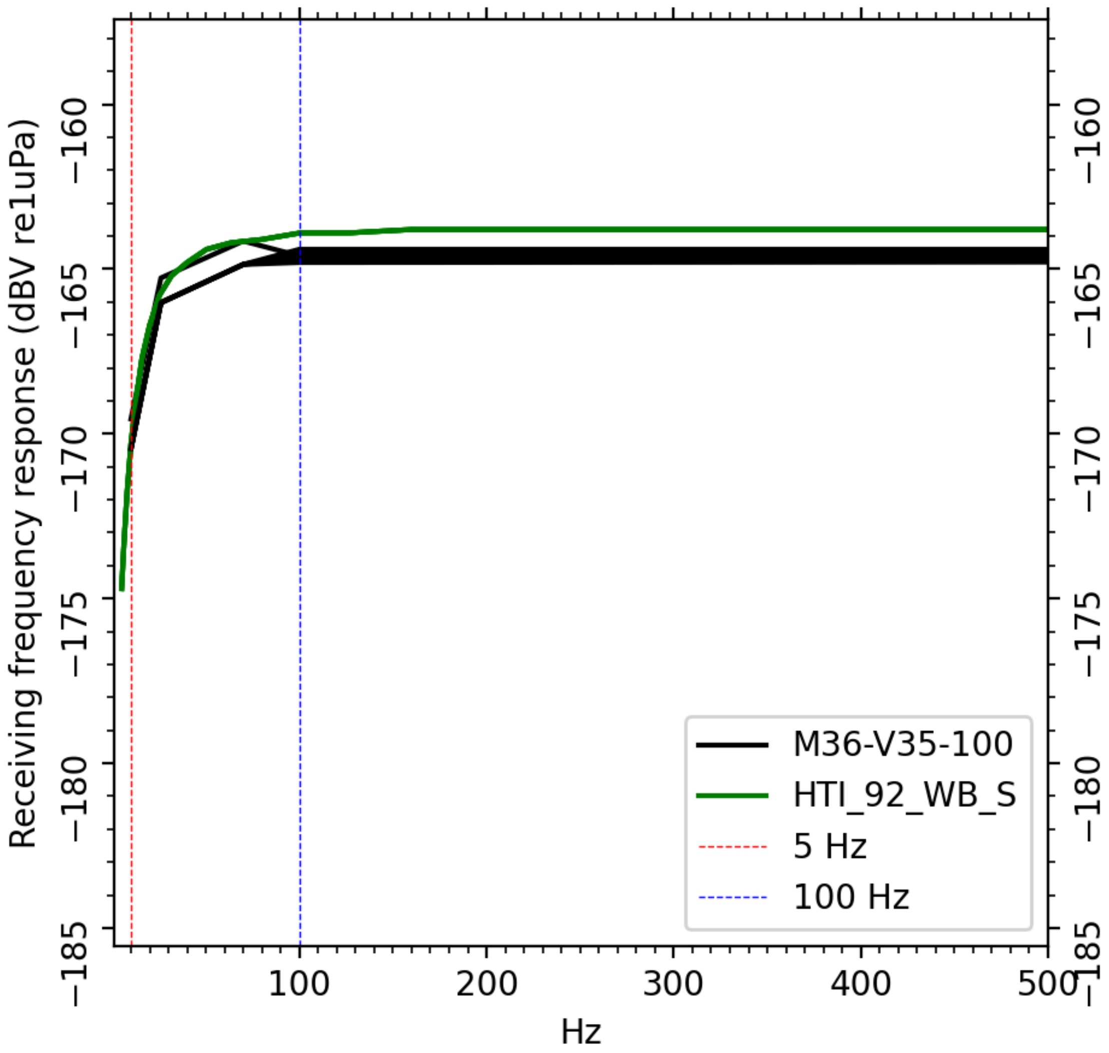

Figure S2: Hydrophone receiving frequency specifications. Typical receiving frequency characteristics of hydrophones types: M36-V35-100 and HTI-92-WB deployed during the Santos Basin underwater soundscape monitoring Project (PMPAS-BS) between 2015 and 2021.

Earth velocity model - ak135f

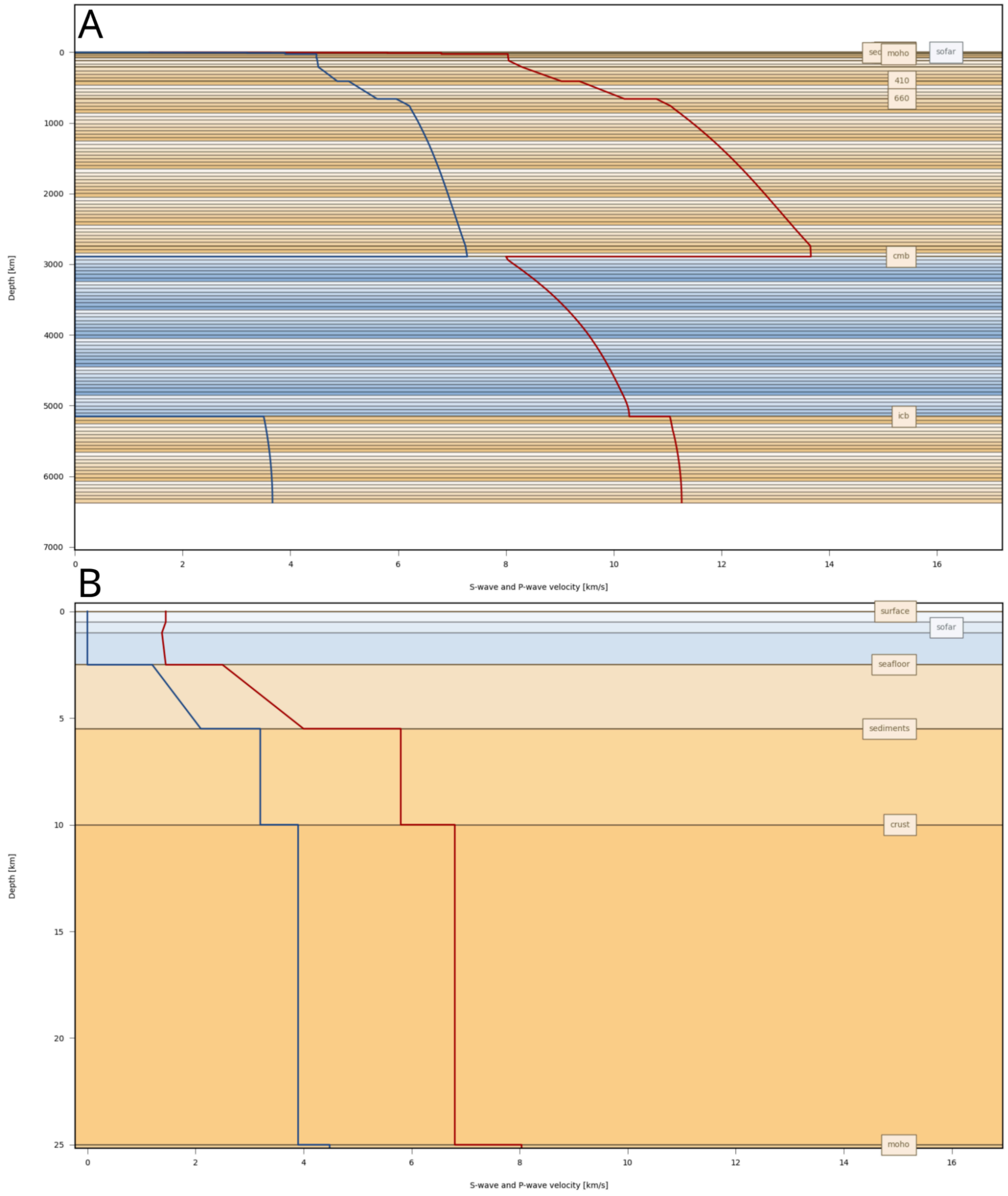

Figure S3: Modified upper crustal structure version of ak135-f. Panel (a) presents the ak135-f Earth model, a variant of the ak135 velocity model, supplemented with a density model and bulk and shear seismic quality factors. Panel (b) provides a zoomed-in view from the the top of the velocity model, depicting a 2.5 km thick water layer, a 3 km thick sedimentary layer, and an average crustal thickness of 25 km.

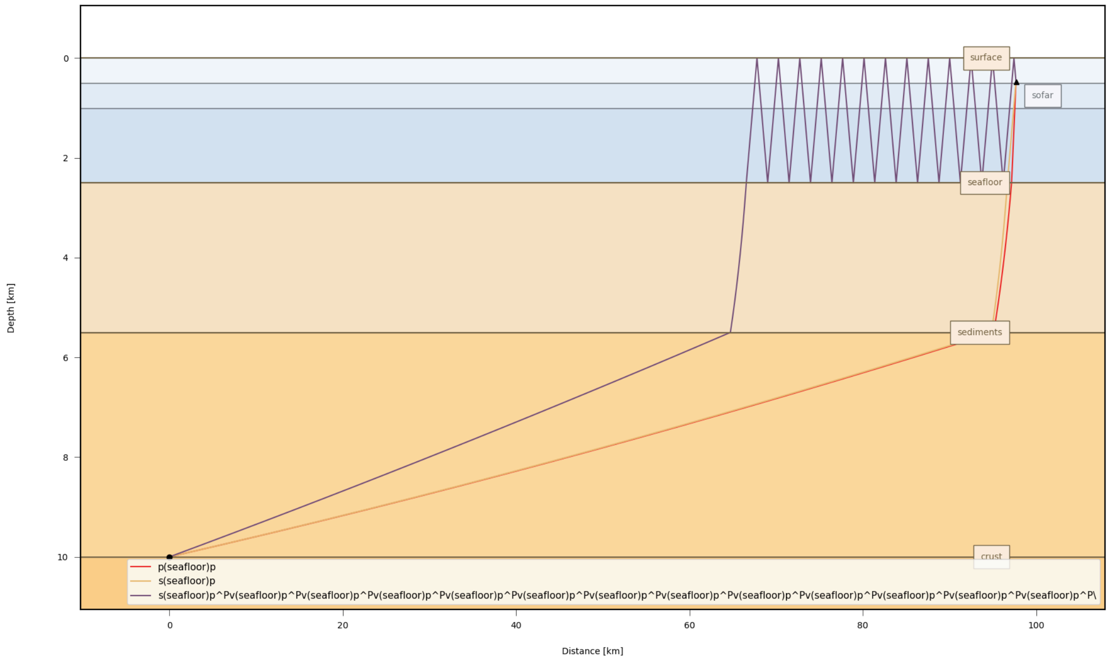

Figure S4: Ray paths for a layered Earth model. Ray paths for a layered Earth model for the following phases: P (p-to-p), S (s-to-p), and T (s-to-T). Computations are done for a spherical earth in the CAKE software (Pyrocko).

Three-dimensional sampling layouts

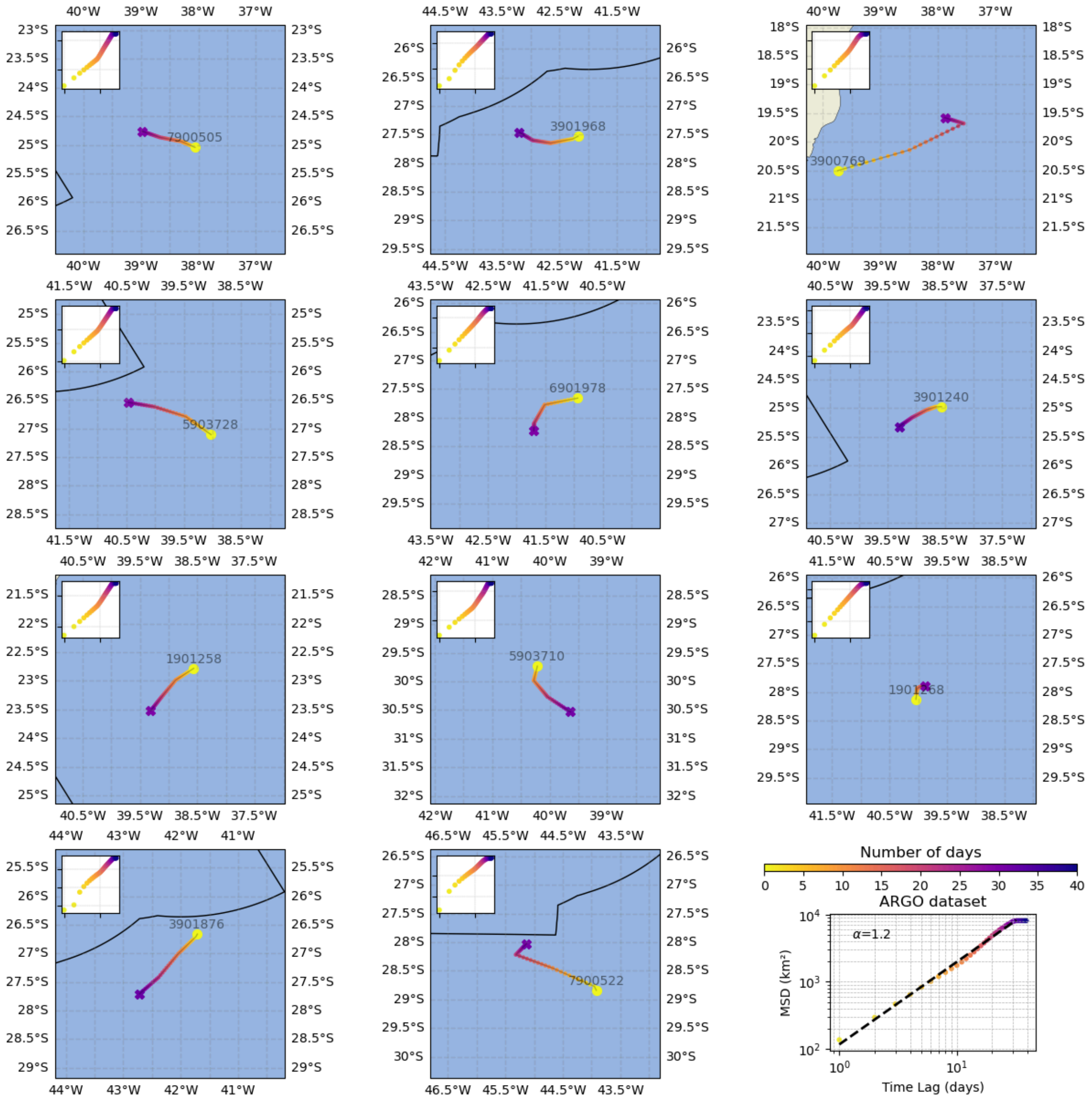

Figure S5. Plot of the 11 ARGOs trajectories near the Santos Basin. Each panel shows the mean squared displacement in the top-left inset. The last panel presents the mean squared displacement (MSD) of the entire dataset versus time on a log-log scale, with the estimated fit shown as a dashed black line. The diffusion exponent (α) is presented. Colors represent the number of days, and the initial and final positions are marked by a large circle and cross, respectively. Time is measured in days, and dispersion is in km² .

Earthquake signal detection

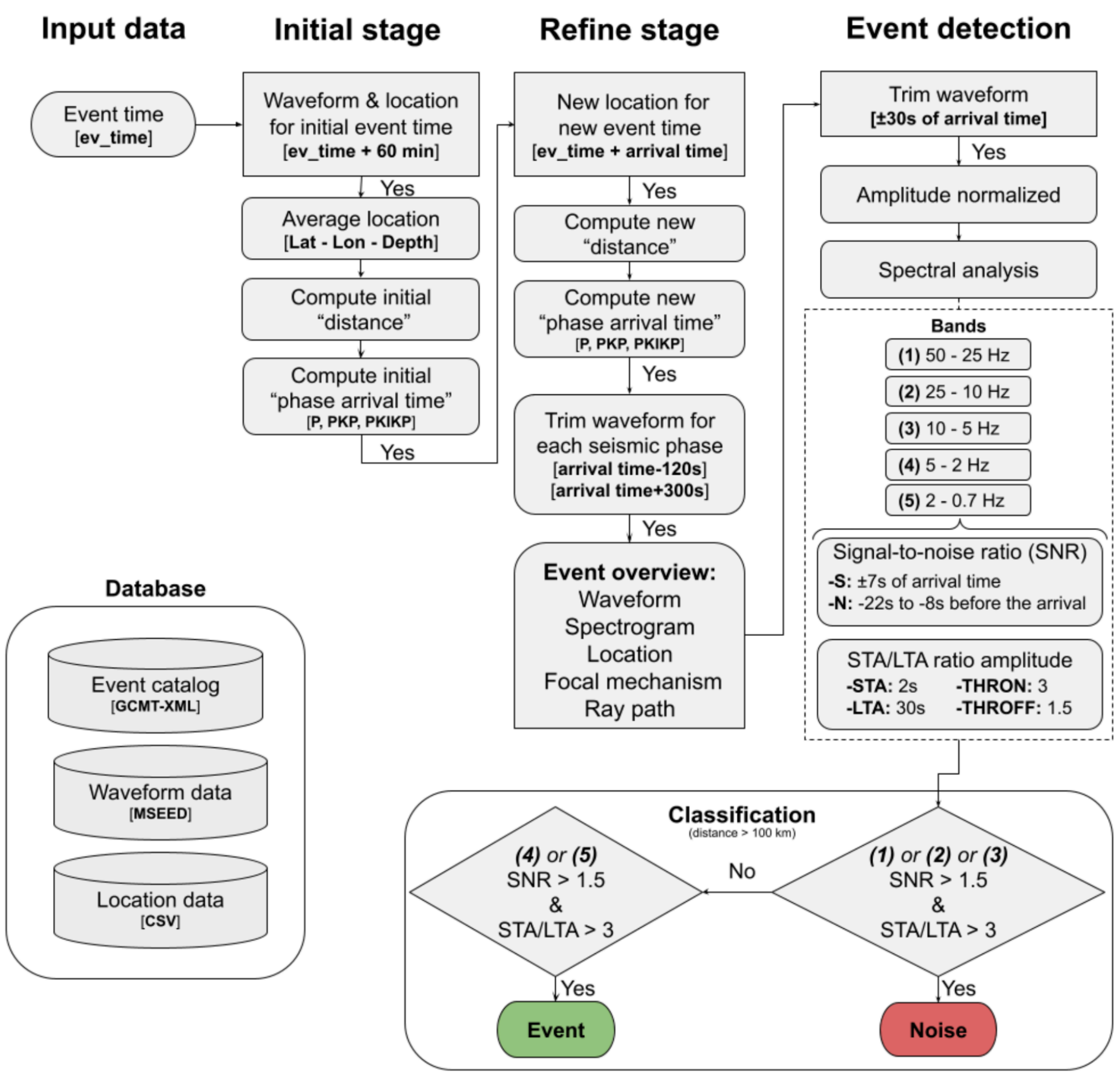

Figure S6: Overview of the automated event detection algorithm. Algorithm to detect global earthquakes (epicentral distance > 100 km) automatically, and the database utilized (bottom left).

Results

Possibly global earthquakes in the glider records

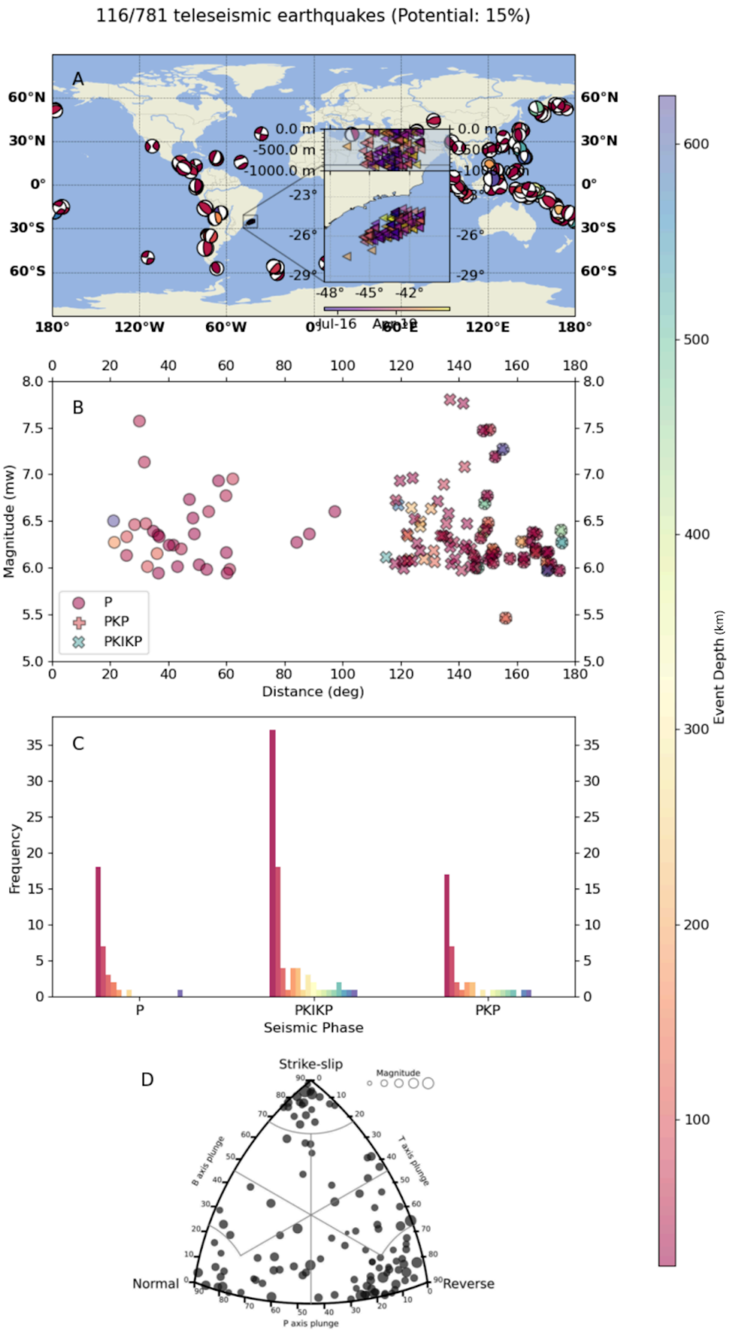

Figure S7: Indications of global earthquakes in glider records. (A) The epicentral locations of the 116 earthquakes are illustrated, indicating both the depth and focal mechanisms of each event. Notably, the central insets highlights the specific locations of the ocean gliders at the moment of registering the P-wave arrival from the teleseismic event. (B) Distributions of epicentral distances and magnitudes, highlighting the depth of each event. (C) Histograms of each potential registered phase. (D) Classification of focal mechanisms divided into three main types: normal, reverse, and strike-slip. Generated using Cartopy (https://scitools.org.uk/cartopy/), a Python library for geospatial visualization.

Spectral analysis

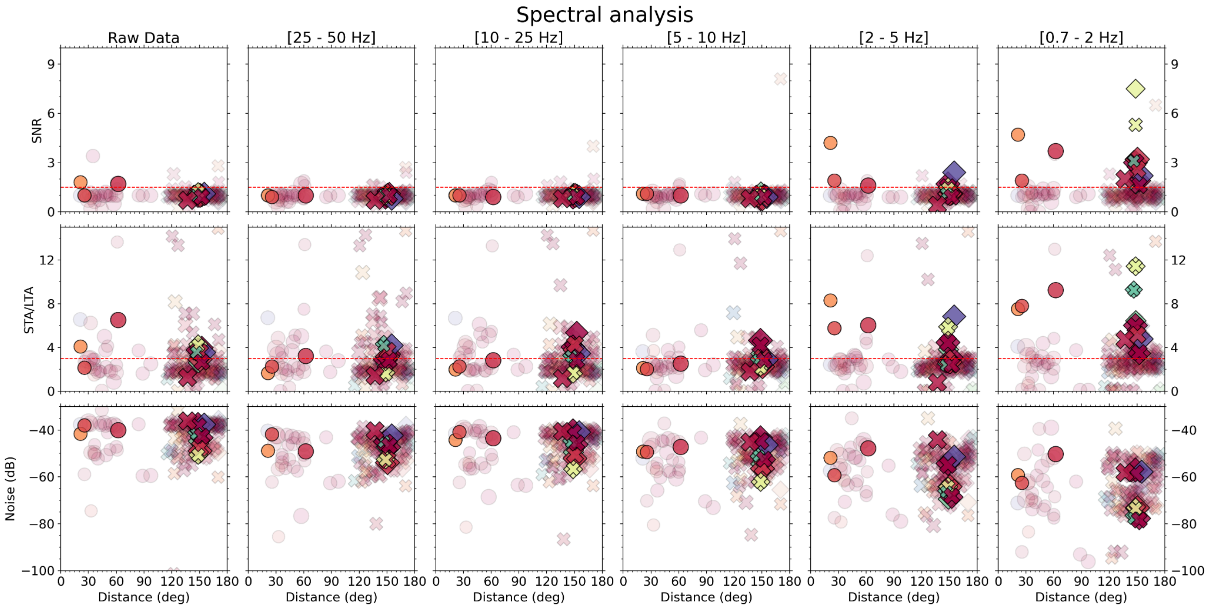

Figure S8: Spectral analysis using the five frequency bands. Spectral analysis using the following frequency bands: 25-50 Hz, 10-25 Hz, 5-10 Hz, 2-5 Hz, 0.7-2 Hz. The rows are the signal-to-noise ratio, STA/LTA ratio, and noise level, respectively. The horizontal red lines show SNR $\geqslant 1.5$ and STA/LTA $\geqslant 3$. The colors and types are illustrated in Figure S7.

Global earthquakes automatically detected

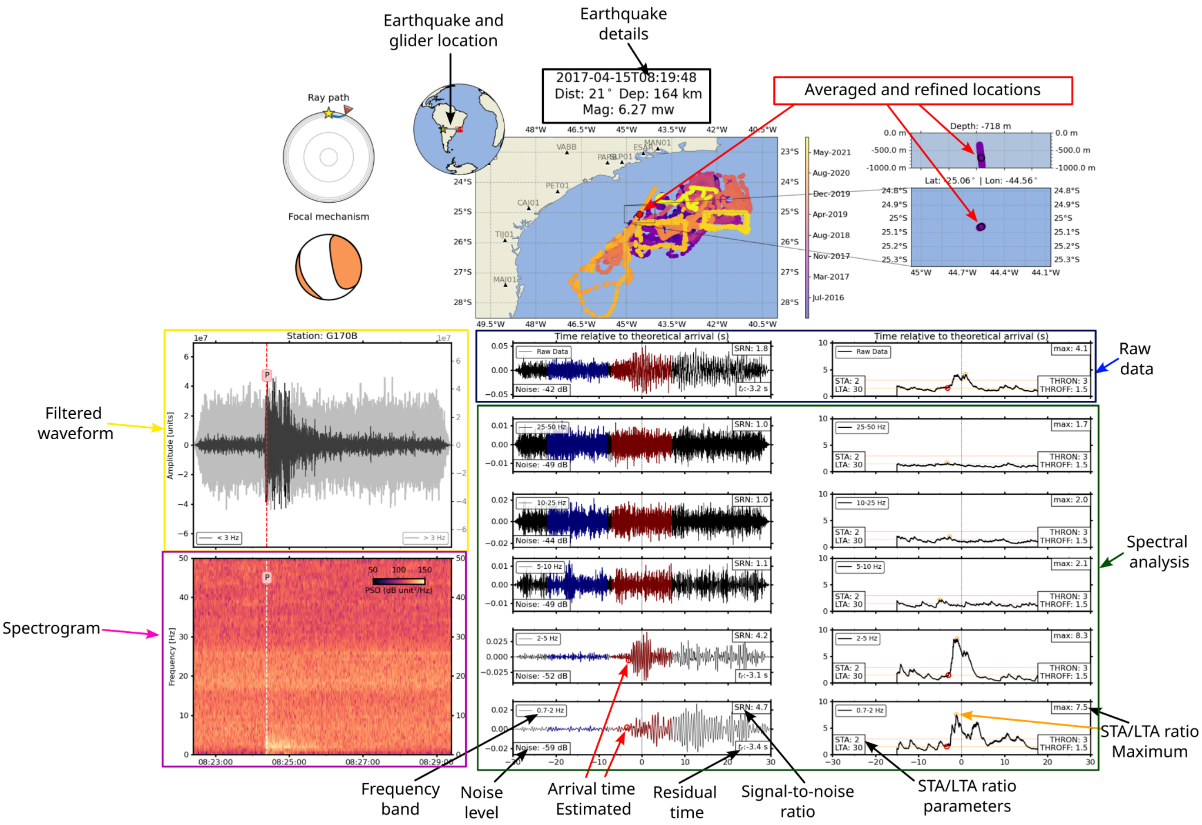

Example and explanation of a mosaic showing the analysis of the magnitude 6.27 mw earthquake that occurred on April 15, 2017, at 08:19:48. Global map showing station-event locations, ray path of the seismic phase, and focal mechanism (colors indicate event depth). Lower panel: wide-window waveform filtered (black and grey lines are waveforms filtered below and above 3 Hz, respectively, and the amplitude is presented in digital units) and its spectrogram. Vertical dashed lines (red/white) and the letter indicate the theoretical arrival time estimated using the modified ak135f model. Spectral analysis using five frequency bands centered on the theoretical wave arrival (between -30 and 30 seconds), waveforms, and STA/LTA ratio curves for each band and the raw data. The red dot indicates the estimated phase onset based on SNR > 1.5 and STA/LTA ratio > 3 in the last two frequency bands (see algorithm). The dashed oranges horizontal lines represent the preset lower (1.5) and upper (3) limits for the STA/LTA ratio trigger, and the orange dot is the maximum value.

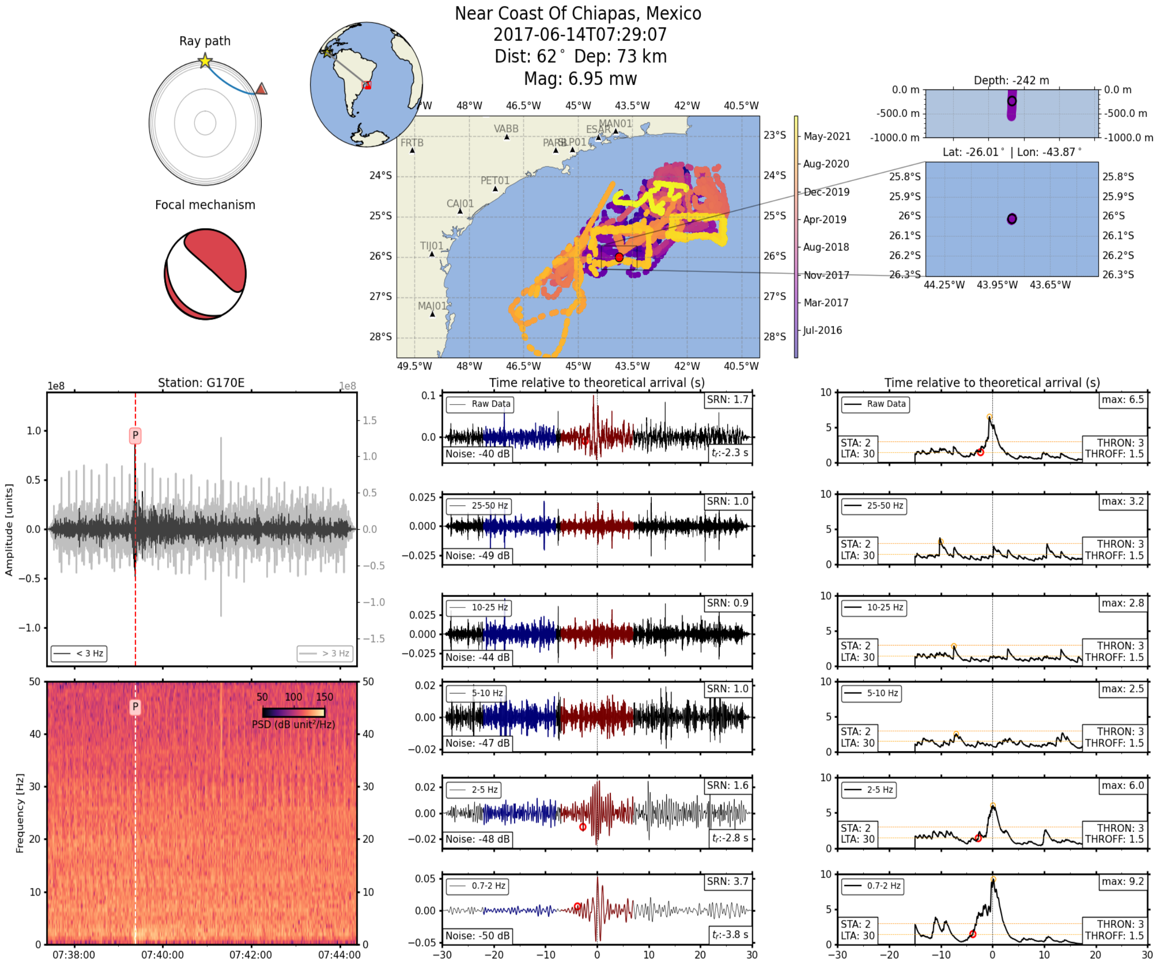

Figure S9: **Waveforms, spectrograms, time series windows centered on the theoretical arrival times, and STA/LTA ratio curves.** Mosaic showing the analysis of the magnitude 6.95 mw earthquake (id: 201706140729A) that occurred on June 14, 2017, at 07:29:07 at Near Coast Of Chiapas, Mexico. Upper panel: Global map showing station-event locations, ray path of the seismic phase, and focal mechanism (colors indicate event depth). Trajectories of the ocean glider and zoomed-in views at the moment of the record in latitude-longitude and depth-longitude plots. Lower panel: wide-window waveform filtered (black and grey lines are waveforms filtered below and above 3 Hz, respectively, and the amplitude is presented in digital units) and its spectrogram. Vertical dashed lines (red/white) and the letter indicate the theoretical arrival time estimated using the ak135f model. Spectral analysis using five frequency bands centered on the theoretical wave arrival (between -30 and 30 seconds), waveforms, and STA/LTA ratio curves for each band and the raw data. The red dot indicates the estimated phase arrival based on SNR > 1.5 and STA/LTA ratio > 3 in the last two frequency bands. The dashed oranges horizontal lines represent the preset lower (1.5) and upper (3) limits for the STA/LTA ratio trigger, and the orange dot is the maximum value.

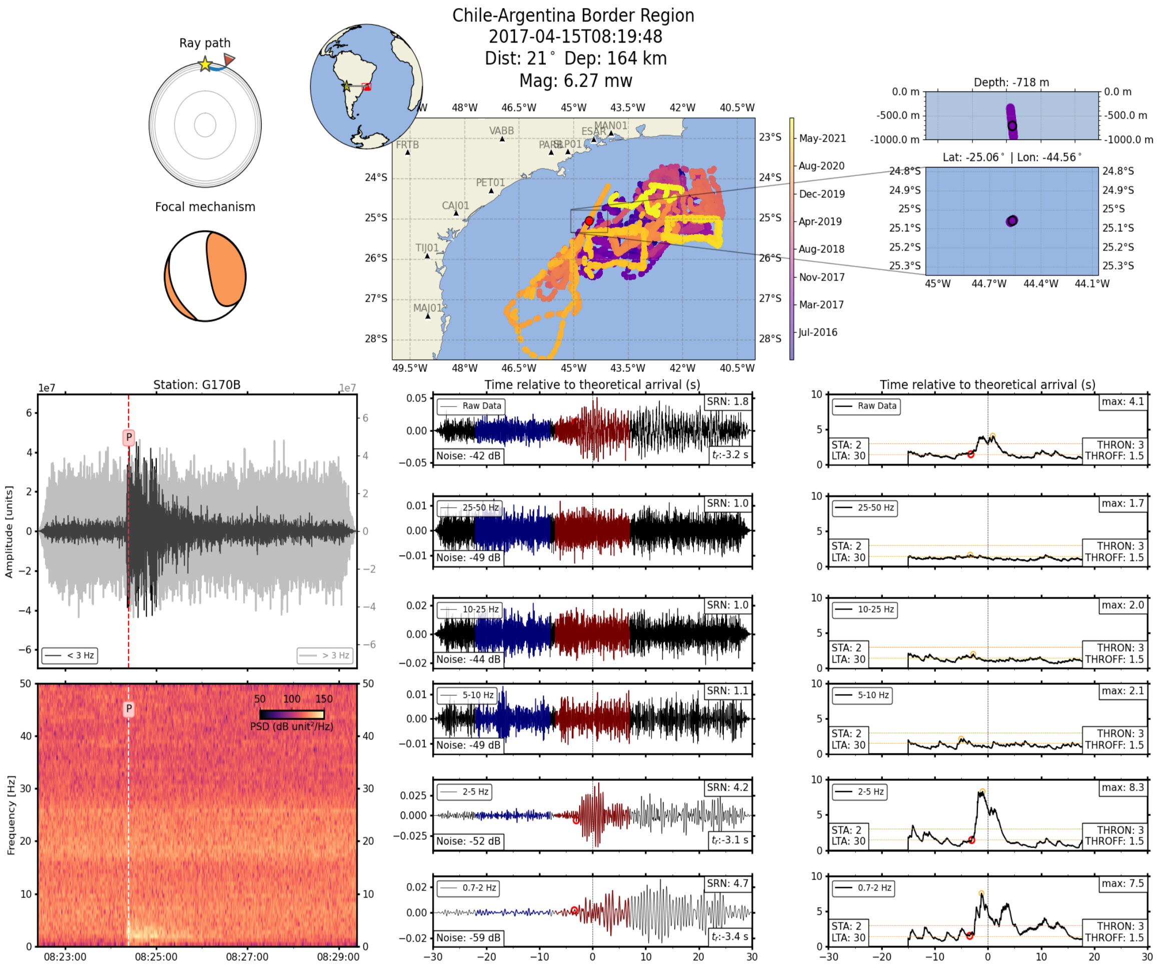

Figure S10: **Waveforms, spectrograms, time series windows centered on the theoretical arrival times, and STA/LTA ratio curves.** Mosaic showing the analysis of the magnitude 6.27 mw earthquake (id: 201704150819A) that occurred on April 15, 2017, at 08:19:48 at Chile-Argentina Border Region. Upper panel: Global map showing station-event locations, ray path of the seismic phase, and focal mechanism (colors indicate event depth). Trajectories of the ocean glider and zoomed-in views at the moment of the record in latitude-longitude and depth-longitude plots. Lower panel: wide-window waveform filtered (black and grey lines are waveforms filtered below and above 3 Hz, respectively, and the amplitude is presented in digital units) and its spectrogram. Vertical dashed lines (red/white) and the letter indicate the theoretical arrival time estimated using the ak135f model. Spectral analysis using five frequency bands centered on the theoretical wave arrival (between -30 and 30 seconds), waveforms, and STA/LTA ratio curves for each band and the raw data. The red dot indicates the estimated phase arrival based on SNR > 1.5 and STA/LTA ratio > 3 in the last two frequency bands. The dashed oranges horizontal lines represent the preset lower (1.5) and upper (3) limits for the STA/LTA ratio trigger, and the orange dot is the maximum value.

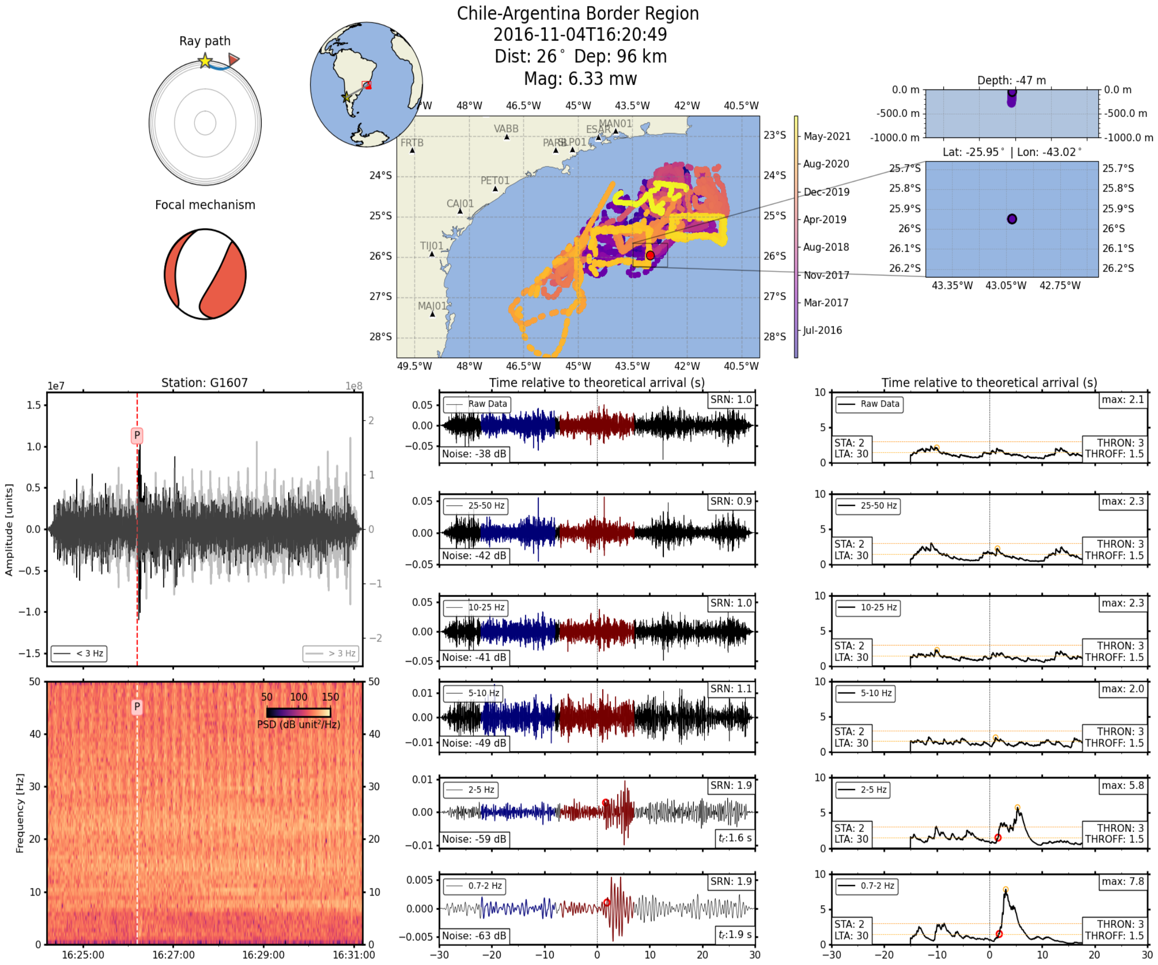

Figure S11: **Waveforms, spectrograms, time series windows centered on the theoretical arrival times, and STA/LTA ratio curves.** Mosaic showing the analysis of the magnitude 6.33 mw earthquake (id: 201611041620A) that occurred on November 04, 2016, at 16:20:49 at Central Chile. Upper panel: Global map showing station-event locations, ray path of the seismic phase, and focal mechanism (colors indicate event depth). Trajectories of the ocean glider and zoomed-in views at the moment of the record in latitude-longitude and depth-longitude plots. Lower panel: wide-window waveform filtered (black and grey lines are waveforms filtered below and above 3 Hz, respectively, and the amplitude is presented in digital units) and its spectrogram. Vertical dashed lines (red/white) and the letter indicate the theoretical arrival time estimated using the ak135f model. Spectral analysis using five frequency bands centered on the theoretical wave arrival (between -30 and 30 seconds), waveforms, and STA/LTA ratio curves for each band and the raw data. The red dot indicates the estimated phase arrival based on SNR > 1.5 and STA/LTA ratio > 3 in the last two frequency bands. The dashed oranges horizontal lines represent the preset lower (1.5) and upper (3) limits for the STA/LTA ratio trigger, and the orange dot is the maximum value.

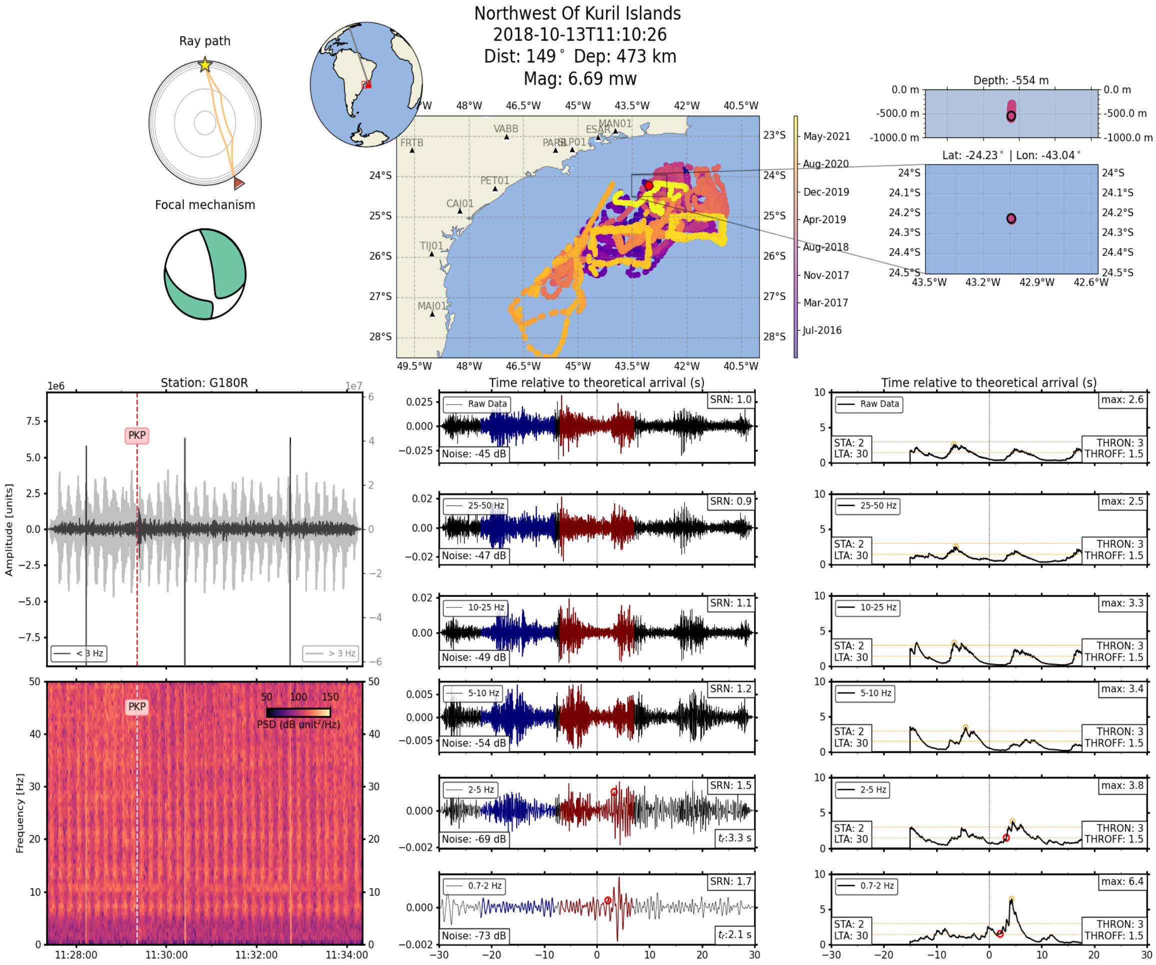

Figure S12: **Waveforms, spectrograms, time series windows centered on the theoretical arrival times, and STA/LTA ratio curves.** Mosaic showing the analysis of the magnitude 6.69 mw earthquake (id: 201810131110A) that occurred on October 13, 2018, at 11:10:26 at Northwest Of Kuril Islands. Upper panel: Global map showing station-event locations, ray path of the seismic phase, and focal mechanism (colors indicate event depth). Trajectories of the ocean glider and zoomed-in views at the moment of the record in latitude-longitude and depth-longitude plots. Lower panel: wide-window waveform filtered (black and grey lines are waveforms filtered below and above 3 Hz, respectively, and the amplitude is presented in digital units) and its spectrogram. Vertical dashed lines (red/white) and the letter indicate the theoretical arrival time estimated using the ak135f model. Spectral analysis using five frequency bands centered on the theoretical wave arrival (between -30 and 30 seconds), waveforms, and STA/LTA ratio curves for each band and the raw data. The red dot indicates the estimated phase arrival based on SNR > 1.5 and STA/LTA ratio > 3 in the last two frequency bands. The dashed oranges horizontal lines represent the preset lower (1.5) and upper (3) limits for the STA/LTA ratio trigger, and the orange dot is the maximum value.

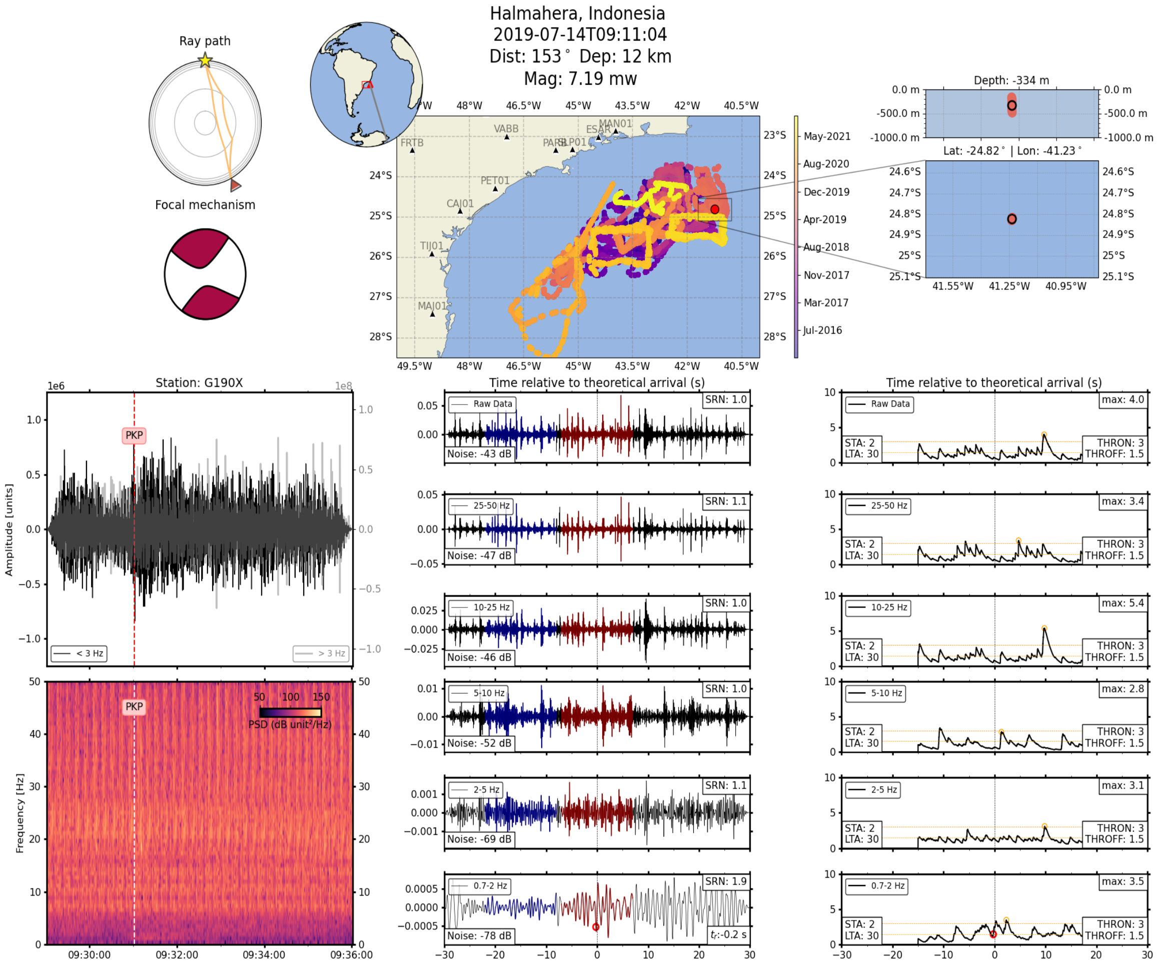

Figure S13: **Waveforms, spectrograms, time series windows centered on the theoretical arrival times, and STA/LTA ratio curves.** Mosaic showing the analysis of the magnitude 7.19 mw earthquake (id: 201907140910A) that occurred on July 14, 2019, at 09:11:04 at Halmahera, Indonesia. Upper panel: Global map showing station-event locations, ray path of the seismic phase, and focal mechanism (colors indicate event depth). Trajectories of the ocean glider and zoomed-in views at the moment of the record in latitude-longitude and depth-longitude plots. Lower panel: wide-window waveform filtered (black and grey lines are waveforms filtered below and above 3 Hz, respectively, and the amplitude is presented in digital units) and its spectrogram. Vertical dashed lines (red/white) and the letter indicate the theoretical arrival time estimated using the ak135f model. Spectral analysis using five frequency bands centered on the theoretical wave arrival (between -30 and 30 seconds), waveforms, and STA/LTA ratio curves for each band and the raw data. The red dot indicates the estimated phase arrival based on SNR > 1.5 and STA/LTA ratio > 3 in the last two frequency bands. The dashed oranges horizontal lines represent the preset lower (1.5) and upper (3) limits for the STA/LTA ratio trigger, and the orange dot is the maximum value.

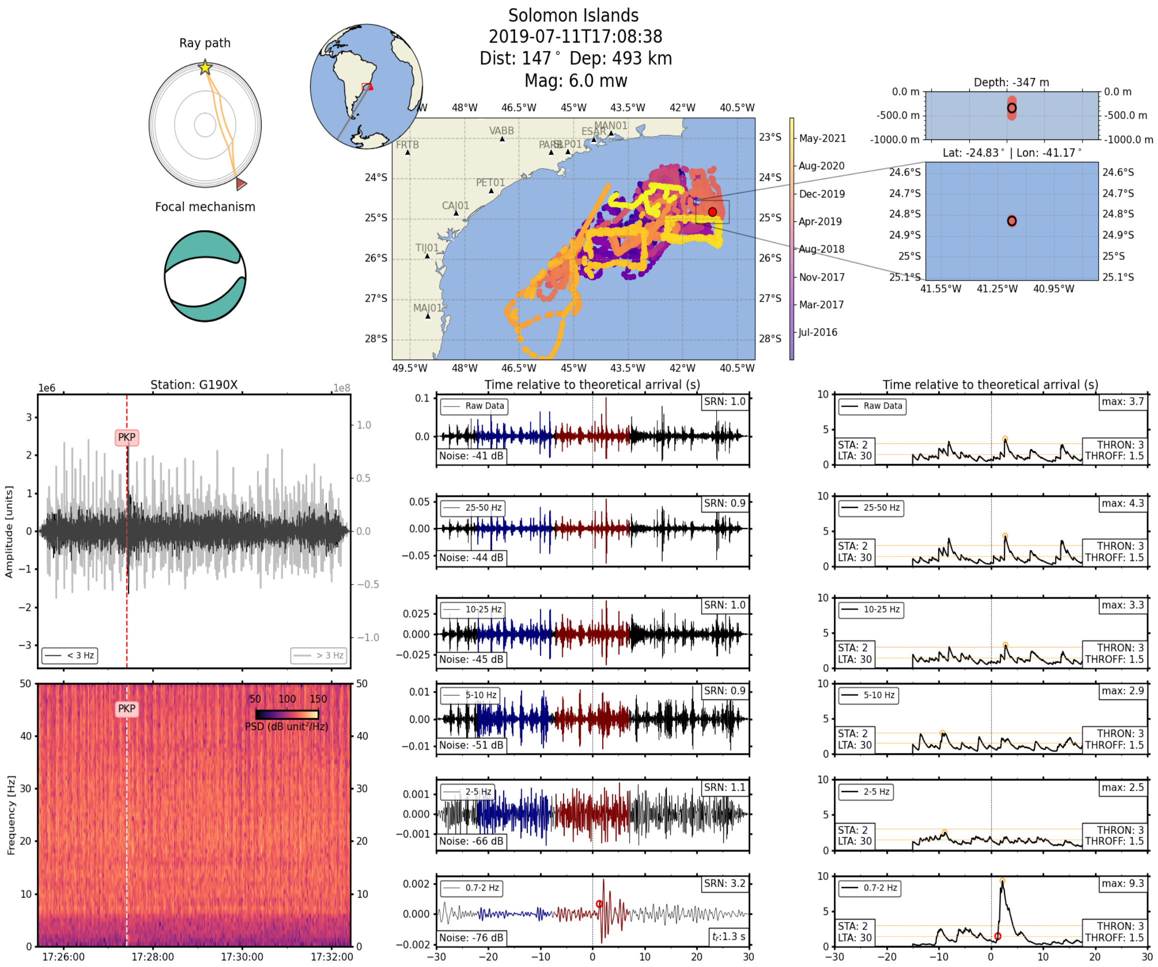

Figure S14: **Waveforms, spectrograms, time series windows centered on the theoretical arrival times, and STA/LTA ratio curves.** Mosaic showing the analysis of the magnitude 6.0 mw earthquake (id: 201907111708A) that occurred on July 11, 2019, at 17:08:38 at Solomon Islands. Upper panel: Global map showing station-event locations, ray path of the seismic phase, and focal mechanism (colors indicate event depth). Trajectories of the ocean glider and zoomed-in views at the moment of the record in latitude-longitude and depth-longitude plots. Lower panel: wide-window waveform filtered (black and grey lines are waveforms filtered below and above 3 Hz, respectively, and the amplitude is presented in digital units) and its spectrogram. Vertical dashed lines (red/white) and the letter indicate the theoretical arrival time estimated using the ak135f model. Spectral analysis using five frequency bands centered on the theoretical wave arrival (between -30 and 30 seconds), waveforms, and STA/LTA ratio curves for each band and the raw data. The red dot indicates the estimated phase arrival based on SNR > 1.5 and STA/LTA ratio > 3 in the last two frequency bands. The dashed oranges horizontal lines represent the preset lower (1.5) and upper (3) limits for the STA/LTA ratio trigger, and the orange dot is the maximum value.

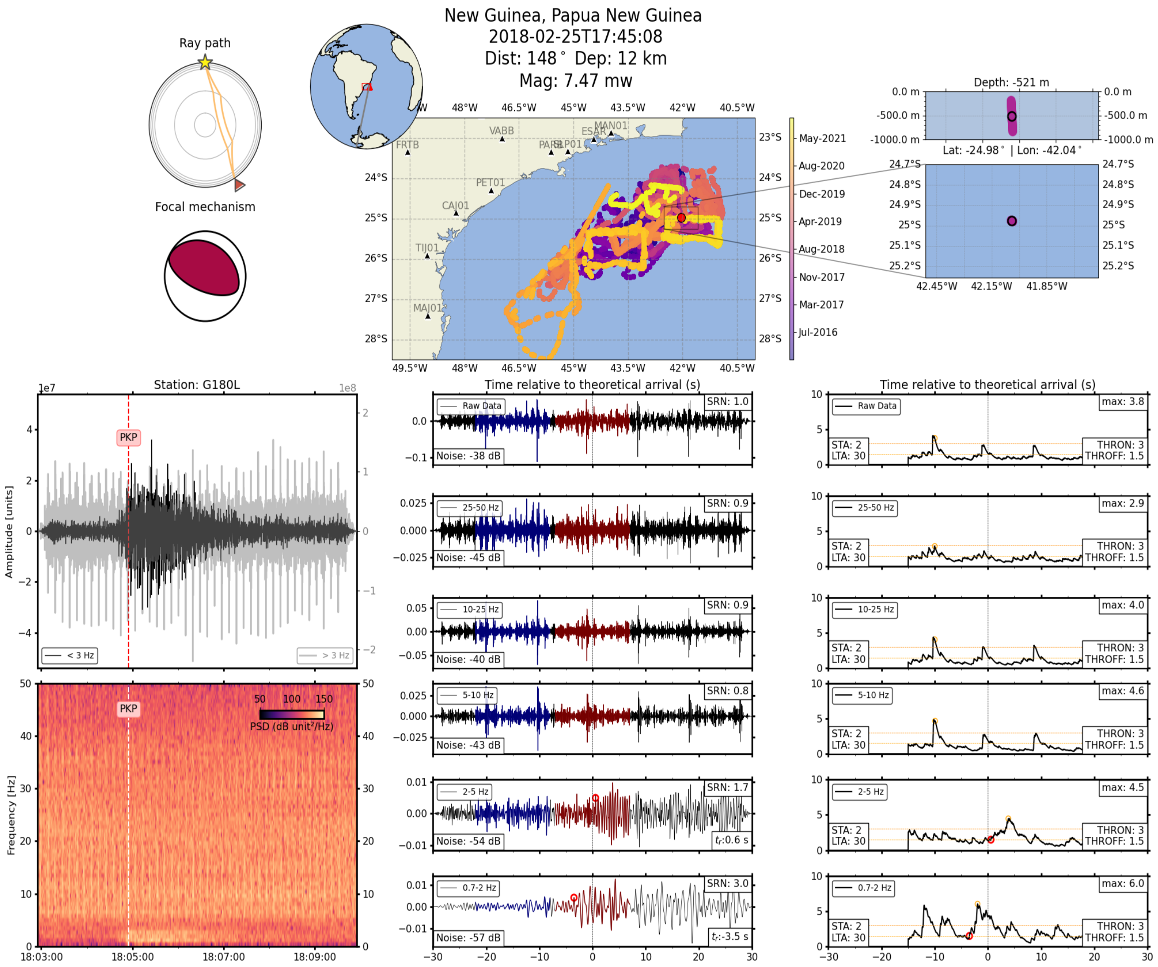

Figure S15: **Waveforms, spectrograms, time series windows centered on the theoretical arrival times, and STA/LTA ratio curves.** Mosaic showing the analysis of the magnitude 7.47 mw earthquake (id: 201802251744A) that occurred on February 25, 2018, at 17:45:08 at New Guinea, Papua New Guinea. Upper panel: Global map showing station-event locations, ray path of the seismic phase, and focal mechanism (colors indicate event depth). Trajectories of the ocean glider and zoomed-in views at the moment of the record in latitude-longitude and depth-longitude plots. Lower panel: wide-window waveform filtered (black and grey lines are waveforms filtered below and above 3 Hz, respectively, and the amplitude is presented in digital units) and its spectrogram. Vertical dashed lines (red/white) and the letter indicate the theoretical arrival time estimated using the ak135f model. Spectral analysis using five frequency bands centered on the theoretical wave arrival (between -30 and 30 seconds), waveforms, and STA/LTA ratio curves for each band and the raw data. The red dot indicates the estimated phase arrival based on SNR > 1.5 and STA/LTA ratio > 3 in the last two frequency bands. The dashed oranges horizontal lines represent the preset lower (1.5) and upper (3) limits for the STA/LTA ratio trigger, and the orange dot is the maximum value.

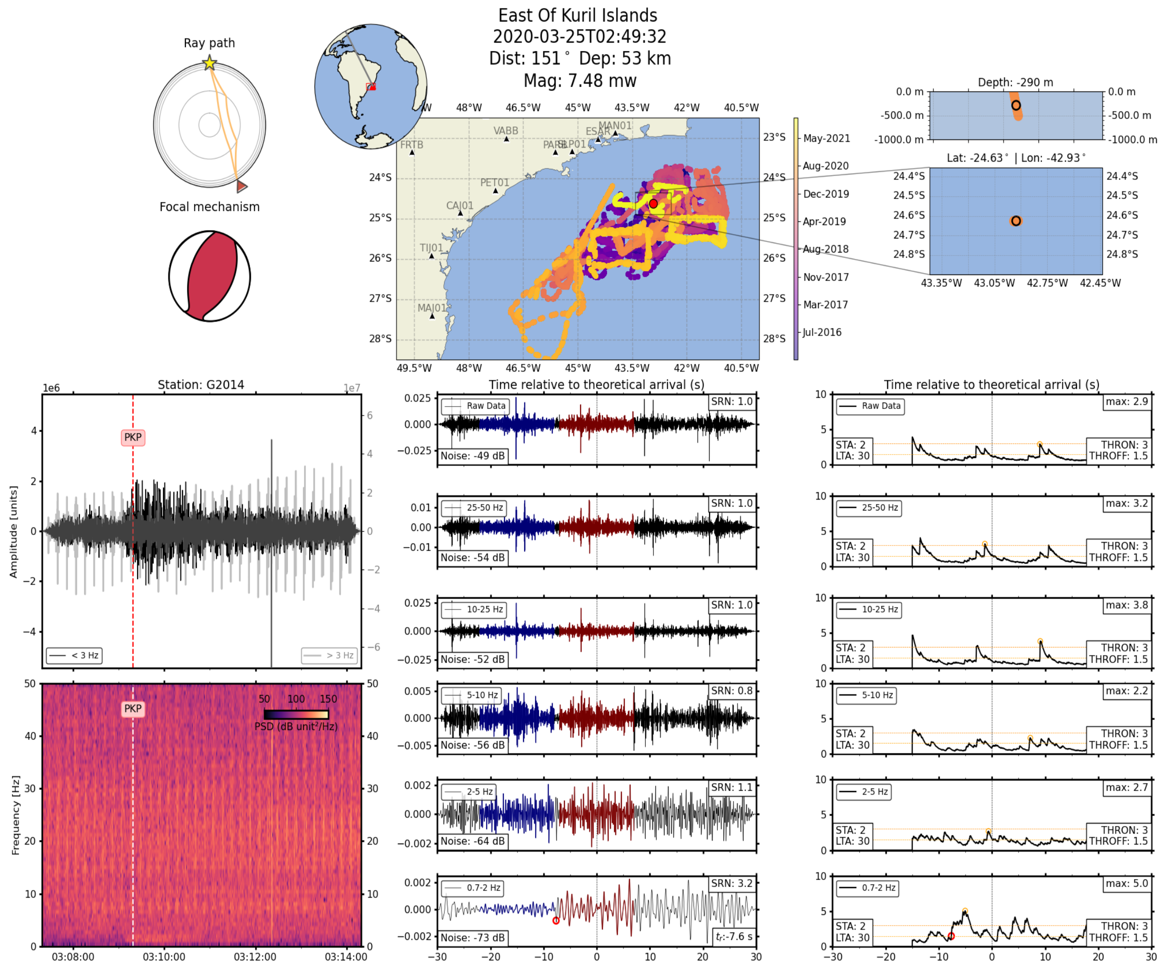

Figure S16: **Waveforms, spectrograms, time series windows centered on the theoretical arrival times, and STA/LTA ratio curves.** Mosaic showing the analysis of the magnitude 7.48 mw earthquake (id: 202003250249A) that occurred on March 25, 2020, at 02:49:32 at East Of Kuril Islands. Upper panel: Global map showing station-event locations, ray path of the seismic phase, and focal mechanism (colors indicate event depth). Trajectories of the ocean glider and zoomed-in views at the moment of the record in latitude-longitude and depth-longitude plots. Lower panel: wide-window waveform filtered (black and grey lines are waveforms filtered below and above 3 Hz, respectively, and the amplitude is presented in digital units) and its spectrogram. Vertical dashed lines (red/white) and the letter indicate the theoretical arrival time estimated using the ak135f model. Spectral analysis using five frequency bands centered on the theoretical wave arrival (between -30 and 30 seconds), waveforms, and STA/LTA ratio curves for each band and the raw data. The red dot indicates the estimated phase arrival based on SNR > 1.5 and STA/LTA ratio > 3 in the last two frequency bands. The dashed oranges horizontal lines represent the preset lower (1.5) and upper (3) limits for the STA/LTA ratio trigger, and the orange dot is the maximum value.

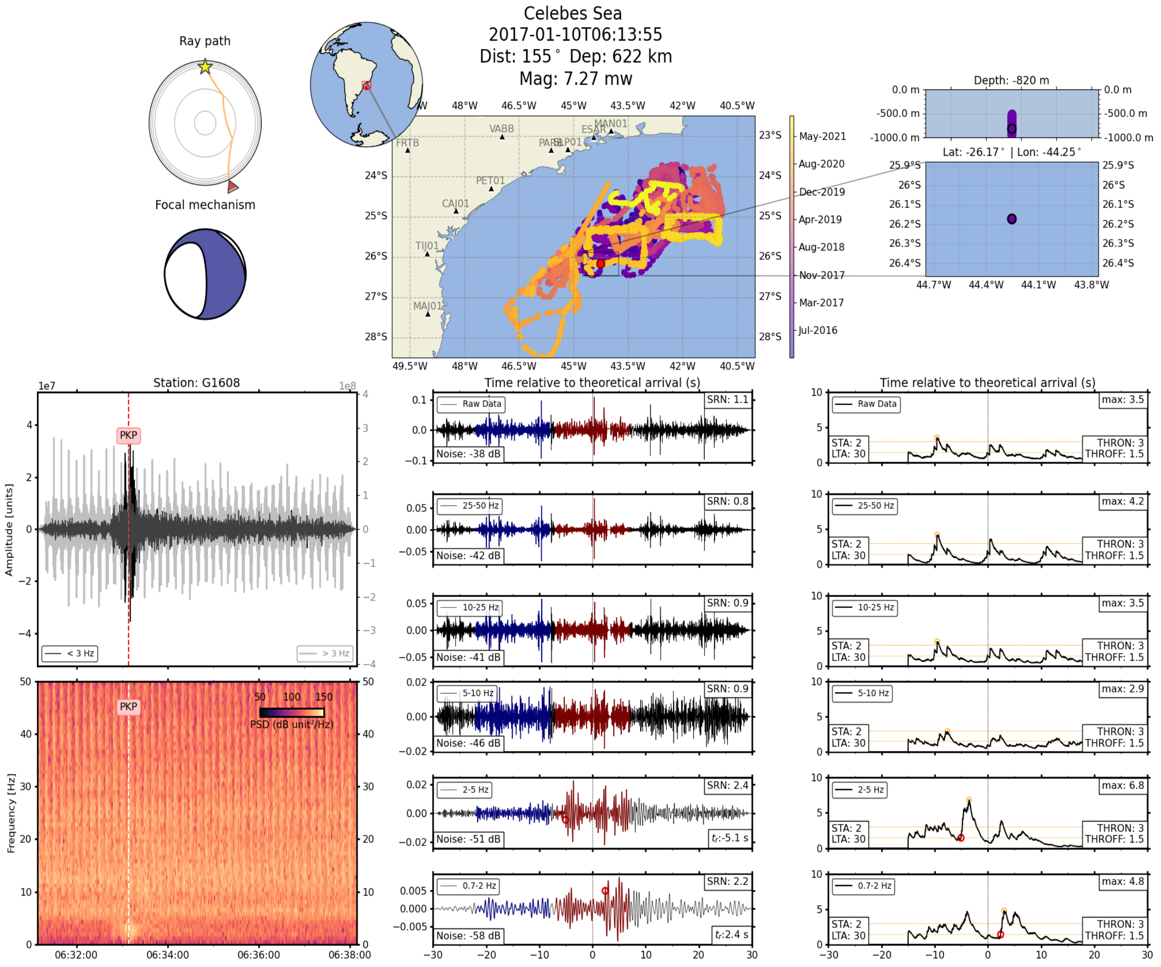

Figure S17: **Waveforms, spectrograms, time series windows centered on the theoretical arrival times, and STA/LTA ratio curves.** Mosaic showing the analysis of the magnitude 7.27 mw earthquake (id: 201701100613A) that occurred on January 10, 2017, at 06:13:55 at Celebes Sea. Upper panel: Global map showing station-event locations, ray path of the seismic phase, and focal mechanism (colors indicate event depth). Trajectories of the ocean glider and zoomed-in views at the moment of the record in latitude-longitude and depth-longitude plots. Lower panel: wide-window waveform filtered (black and grey lines are waveforms filtered below and above 3 Hz, respectively, and the amplitude is presented in digital units) and its spectrogram. Vertical dashed lines (red/white) and the letter indicate the theoretical arrival time estimated using the ak135f model. Spectral analysis using five frequency bands centered on the theoretical wave arrival (between -30 and 30 seconds), waveforms, and STA/LTA ratio curves for each band and the raw data. The red dot indicates the estimated phase arrival based on SNR > 1.5 and STA/LTA ratio > 3 in the last two frequency bands. The dashed oranges horizontal lines represent the preset lower (1.5) and upper (3) limits for the STA/LTA ratio trigger, and the orange dot is the maximum value.

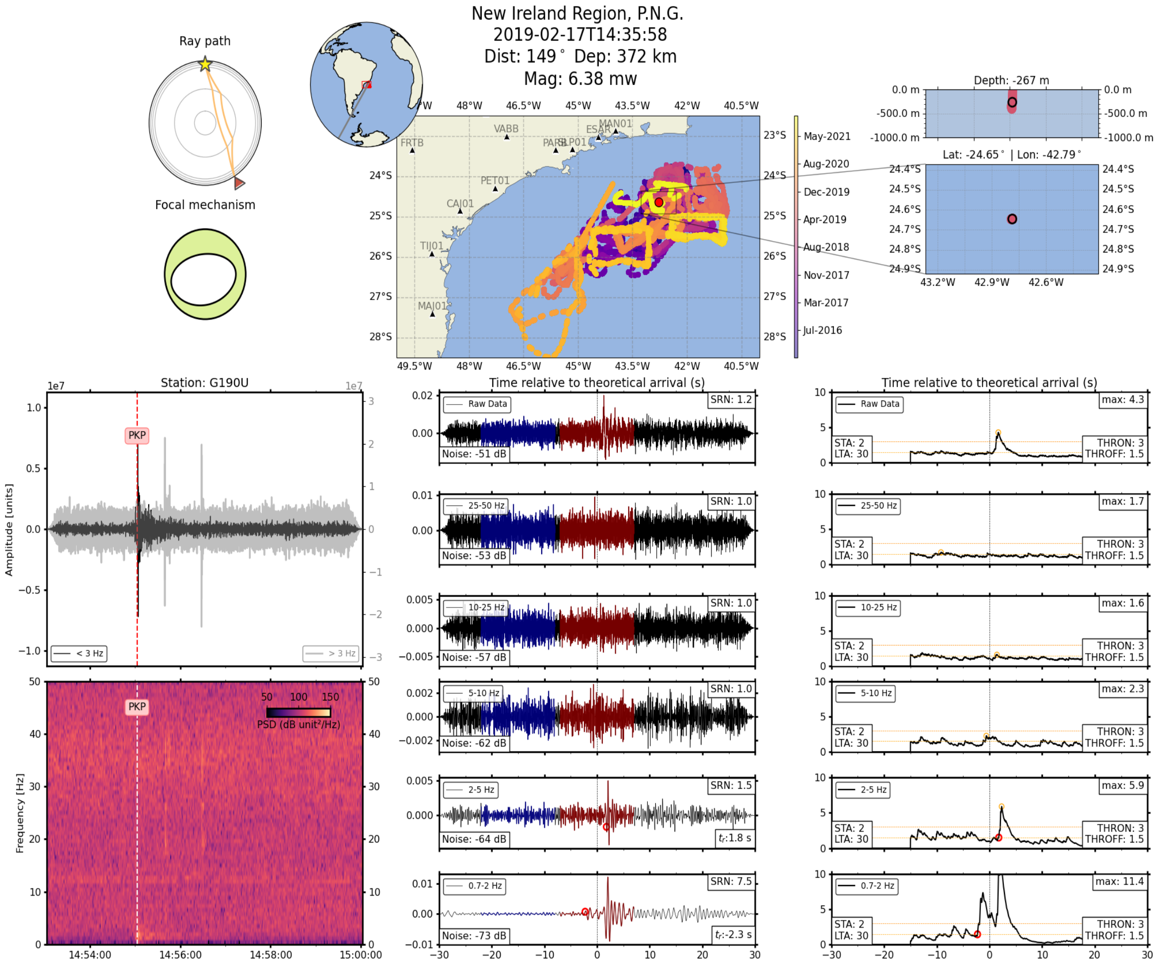

Figure S18: **Waveforms, spectrograms, time series windows centered on the theoretical arrival times, and STA/LTA ratio curves.** Mosaic showing the analysis of the magnitude 6.38 mw earthquake (id: 201902171435A) that occurred on February 17, 2019, at 14:35:58 at New Ireland Region, P.N.G.. Upper panel: Global map showing station-event locations, ray path of the seismic phase, and focal mechanism (colors indicate event depth). Trajectories of the ocean glider and zoomed-in views at the moment of the record in latitude-longitude and depth-longitude plots. Lower panel: wide-window waveform filtered (black and grey lines are waveforms filtered below and above 3 Hz, respectively, and the amplitude is presented in digital units) and its spectrogram. Vertical dashed lines (red/white) and the letter indicate the theoretical arrival time estimated using the ak135f model. Spectral analysis using five frequency bands centered on the theoretical wave arrival (between -30 and 30 seconds), waveforms, and STA/LTA ratio curves for each band and the raw data. The red dot indicates the estimated phase arrival based on SNR > 1.5 and STA/LTA ratio > 3 in the last two frequency bands. The dashed oranges horizontal lines represent the preset lower (1.5) and upper (3) limits for the STA/LTA ratio trigger, and the orange dot is the maximum value.

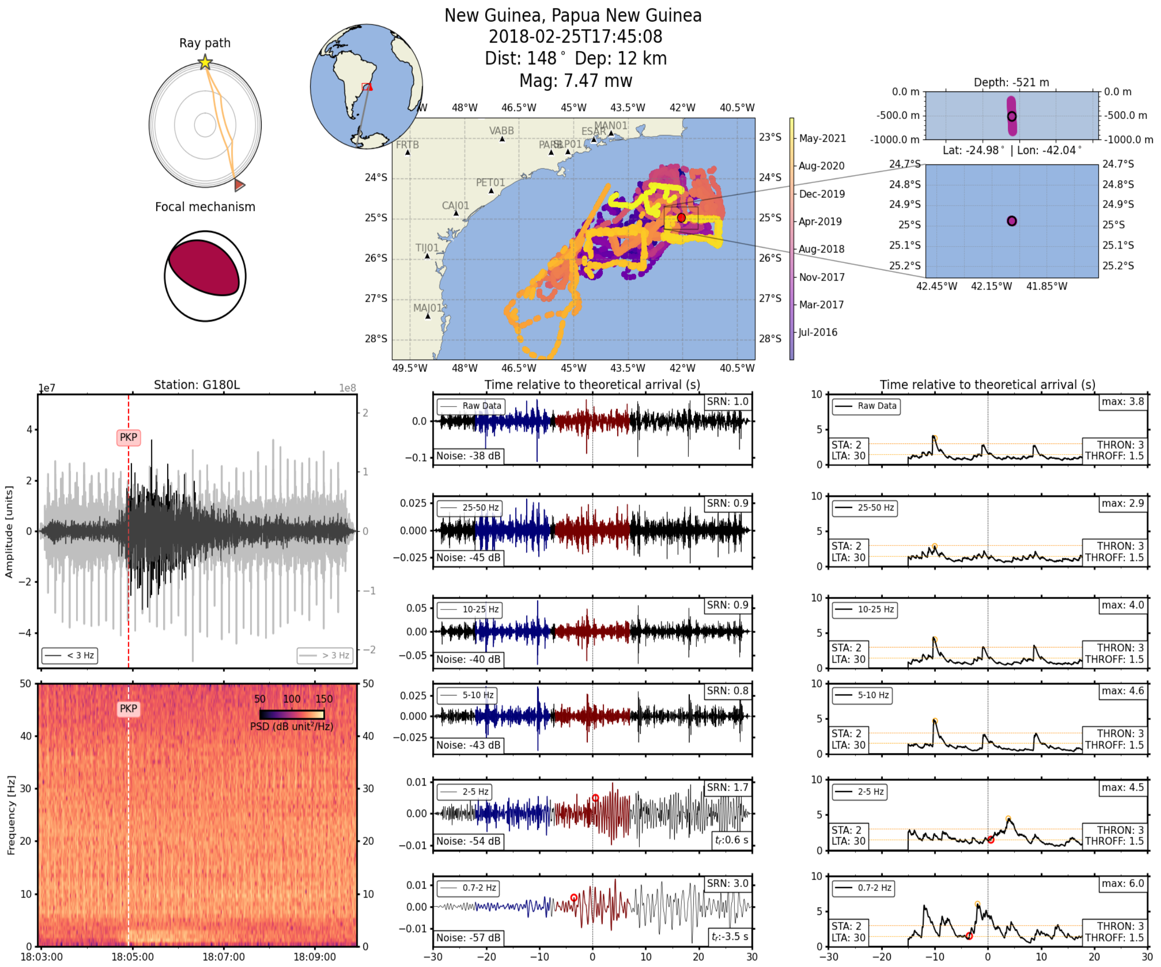

Figure S19: **Waveforms, spectrograms, time series windows centered on the theoretical arrival times, and STA/LTA ratio curves.** Mosaic showing the analysis of the magnitude 7.47 mw earthquake (id: 201802251744A) that occurred on February 25, 2018, at 17:45:08 at New Guinea, Papua New Guinea. Upper panel: Global map showing station-event locations, ray path of the seismic phase, and focal mechanism (colors indicate event depth). Trajectories of the ocean glider and zoomed-in views at the moment of the record in latitude-longitude and depth-longitude plots. Lower panel: wide-window waveform filtered (black and grey lines are waveforms filtered below and above 3 Hz, respectively, and the amplitude is presented in digital units) and its spectrogram. Vertical dashed lines (red/white) and the letter indicate the theoretical arrival time estimated using the ak135f model. Spectral analysis using five frequency bands centered on the theoretical wave arrival (between -30 and 30 seconds), waveforms, and STA/LTA ratio curves for each band and the raw data. The red dot indicates the estimated phase arrival based on SNR > 1.5 and STA/LTA ratio > 3 in the last two frequency bands. The dashed oranges horizontal lines represent the preset lower (1.5) and upper (3) limits for the STA/LTA ratio trigger, and the orange dot is the maximum value.

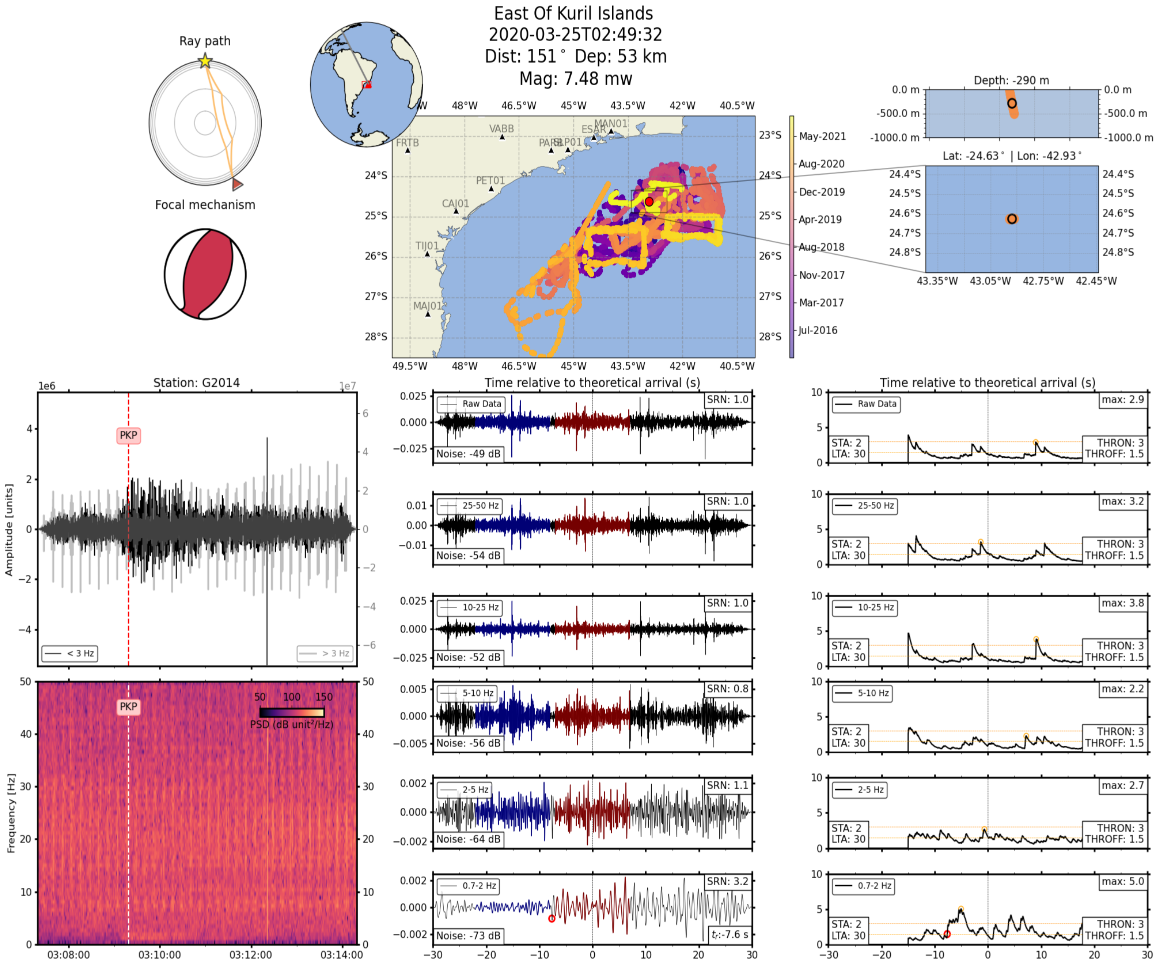

Figure S20: **Waveforms, spectrograms, time series windows centered on the theoretical arrival times, and STA/LTA ratio curves.** Mosaic showing the analysis of the magnitude 7.48 mw earthquake (id: 202003250249A) that occurred on March 25, 2020, at 02:49:32 at East Of Kuril Islands. Upper panel: Global map showing station-event locations, ray path of the seismic phase, and focal mechanism (colors indicate event depth). Trajectories of the ocean glider and zoomed-in views at the moment of the record in latitude-longitude and depth-longitude plots. Lower panel: wide-window waveform filtered (black and grey lines are waveforms filtered below and above 3 Hz, respectively, and the amplitude is presented in digital units) and its spectrogram. Vertical dashed lines (red/white) and the letter indicate the theoretical arrival time estimated using the ak135f model. Spectral analysis using five frequency bands centered on the theoretical wave arrival (between -30 and 30 seconds), waveforms, and STA/LTA ratio curves for each band and the raw data. The red dot indicates the estimated phase arrival based on SNR > 1.5 and STA/LTA ratio > 3 in the last two frequency bands. The dashed oranges horizontal lines represent the preset lower (1.5) and upper (3) limits for the STA/LTA ratio trigger, and the orange dot is the maximum value.

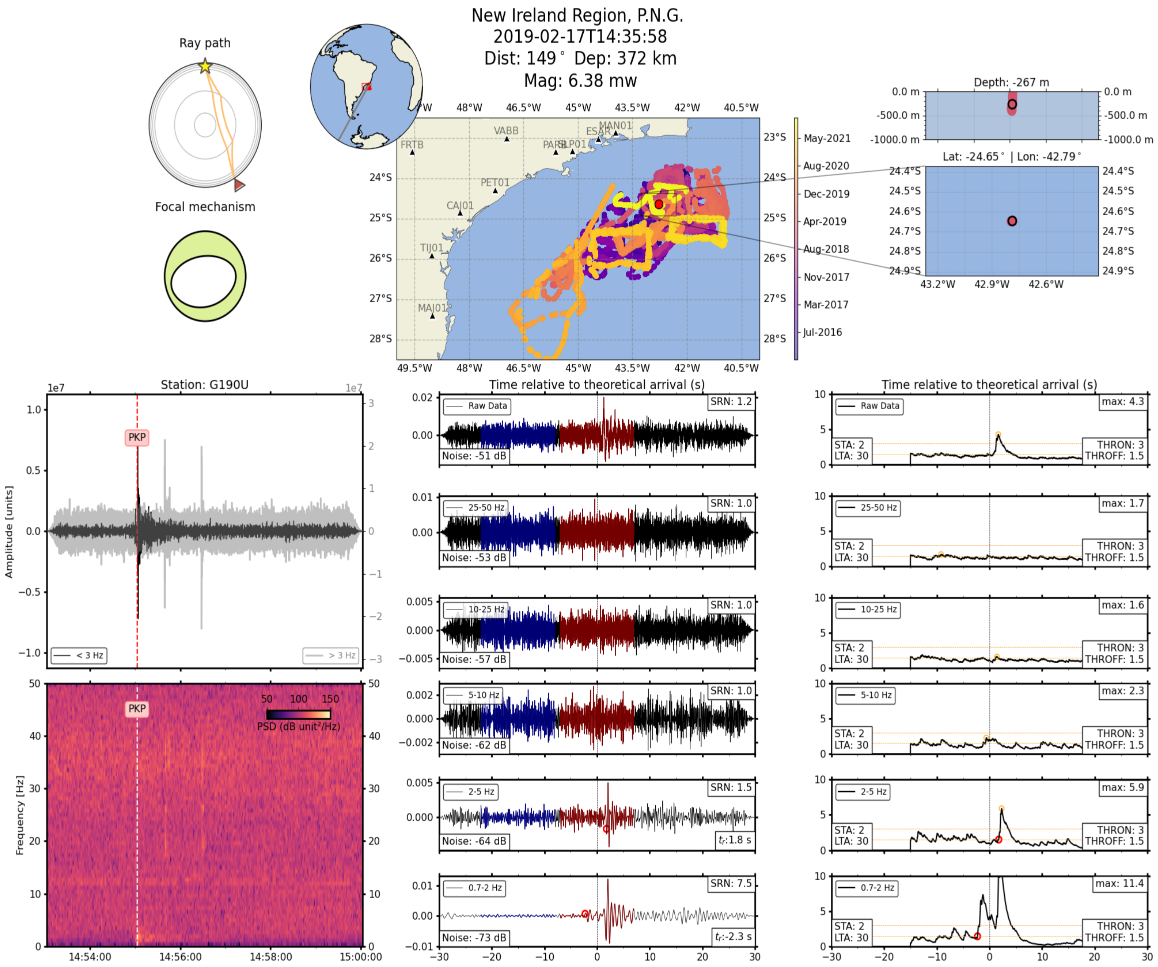

Figure S21: **Waveforms, spectrograms, time series windows centered on the theoretical arrival times, and STA/LTA ratio curves.** Mosaic showing the analysis of the magnitude 6.38 mw earthquake (id: 201902171435A) that occurred on February 17, 2019, at 14:35:58 at New Ireland Region, P.N.G.. Upper panel: Global map showing station-event locations, ray path of the seismic phase, and focal mechanism (colors indicate event depth). Trajectories of the ocean glider and zoomed-in views at the moment of the record in latitude-longitude and depth-longitude plots. Lower panel: wide-window waveform filtered (black and grey lines are waveforms filtered below and above 3 Hz, respectively, and the amplitude is presented in digital units) and its spectrogram. Vertical dashed lines (red/white) and the letter indicate the theoretical arrival time estimated using the ak135f model. Spectral analysis using five frequency bands centered on the theoretical wave arrival (between -30 and 30 seconds), waveforms, and STA/LTA ratio curves for each band and the raw data. The red dot indicates the estimated phase arrival based on SNR > 1.5 and STA/LTA ratio > 3 in the last two frequency bands. The dashed oranges horizontal lines represent the preset lower (1.5) and upper (3) limits for the STA/LTA ratio trigger, and the orange dot is the maximum value.

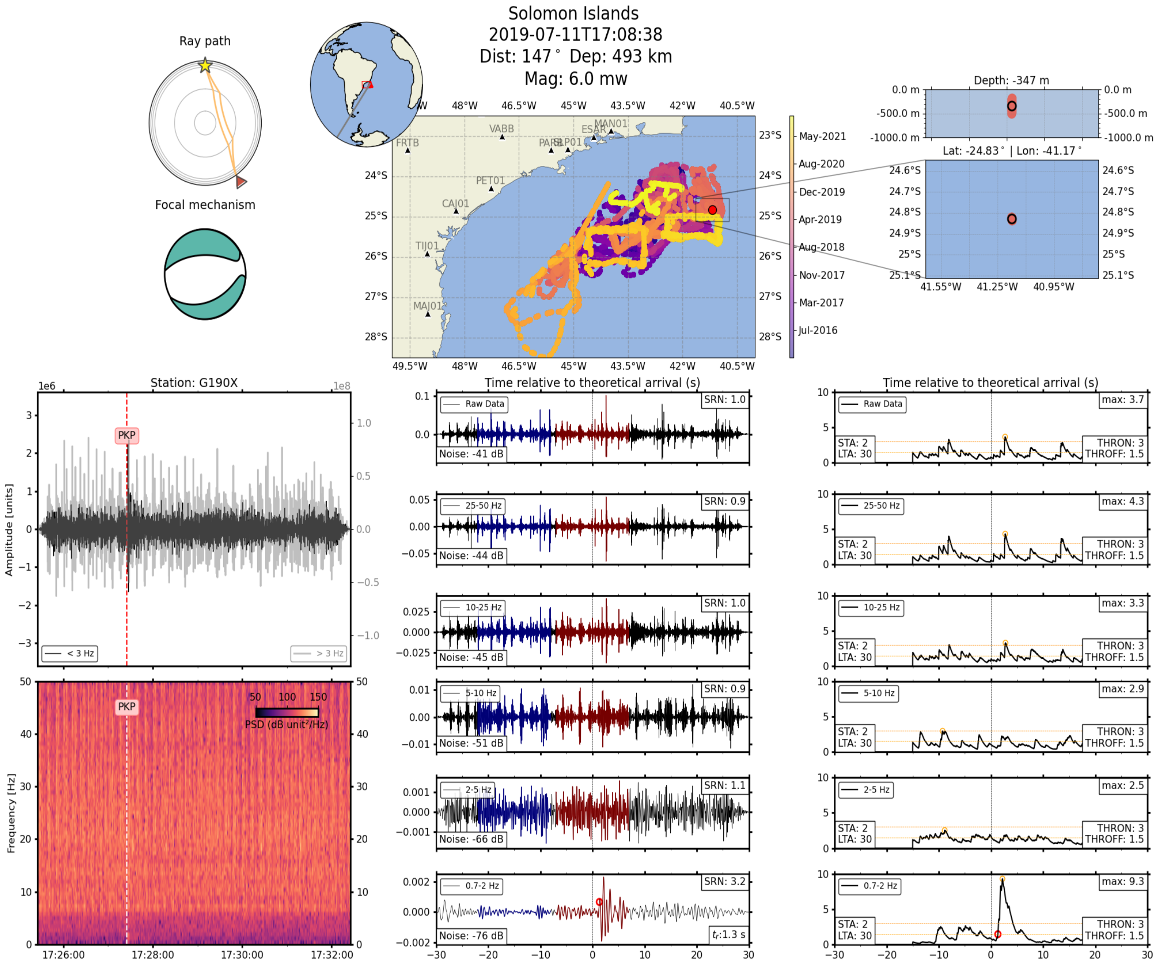

Figure S22: **Waveforms, spectrograms, time series windows centered on the theoretical arrival times, and STA/LTA ratio curves.** Mosaic showing the analysis of the magnitude 6.0 mw earthquake (id: 201907111708A) that occurred on July 11, 2019, at 17:08:38 at Solomon Islands. Upper panel: Global map showing station-event locations, ray path of the seismic phase, and focal mechanism (colors indicate event depth). Trajectories of the ocean glider and zoomed-in views at the moment of the record in latitude-longitude and depth-longitude plots. Lower panel: wide-window waveform filtered (black and grey lines are waveforms filtered below and above 3 Hz, respectively, and the amplitude is presented in digital units) and its spectrogram. Vertical dashed lines (red/white) and the letter indicate the theoretical arrival time estimated using the ak135f model. Spectral analysis using five frequency bands centered on the theoretical wave arrival (between -30 and 30 seconds), waveforms, and STA/LTA ratio curves for each band and the raw data. The red dot indicates the estimated phase arrival based on SNR > 1.5 and STA/LTA ratio > 3 in the last two frequency bands. The dashed oranges horizontal lines represent the preset lower (1.5) and upper (3) limits for the STA/LTA ratio trigger, and the orange dot is the maximum value.

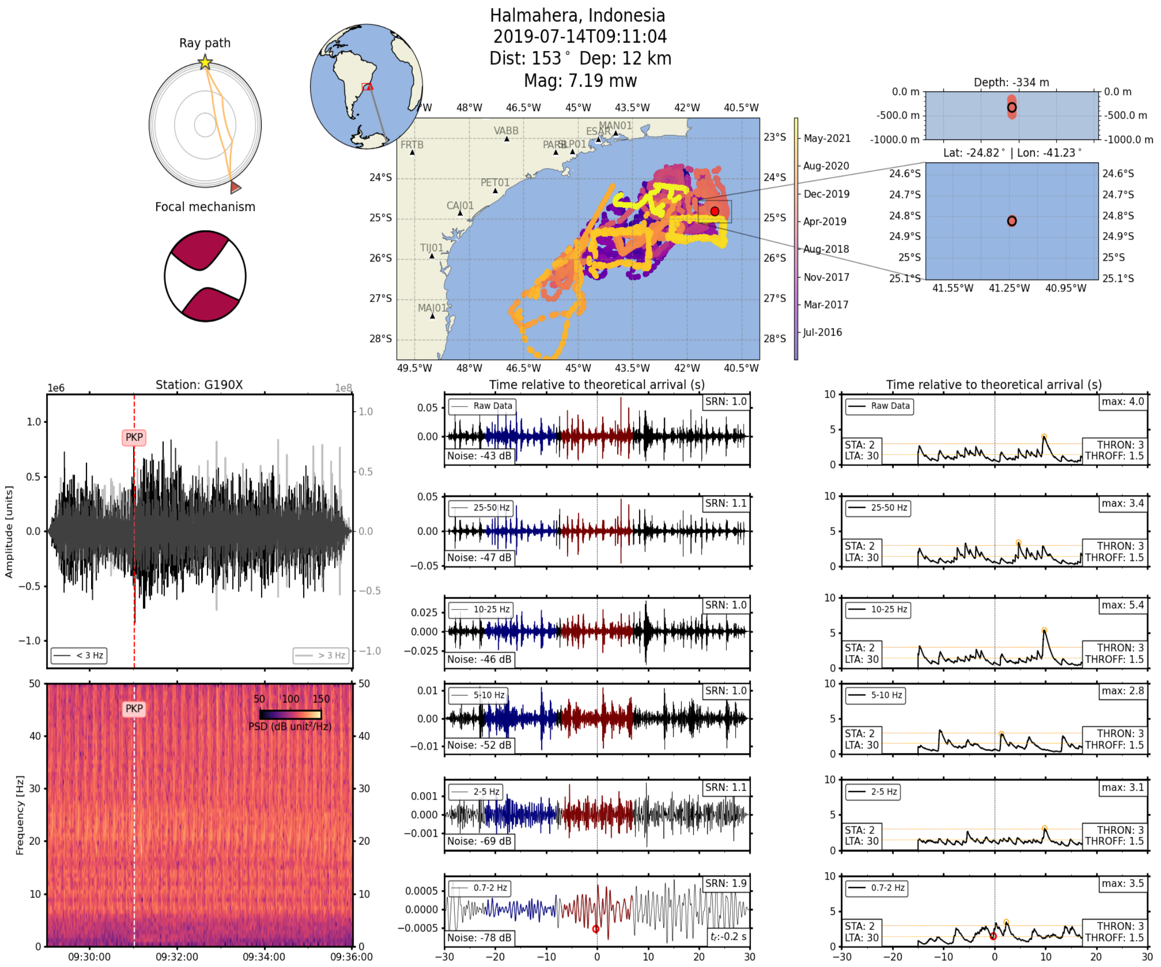

Figure S23: **Waveforms, spectrograms, time series windows centered on the theoretical arrival times, and STA/LTA ratio curves.** Mosaic showing the analysis of the magnitude 7.19 mw earthquake (id: 201907140910A) that occurred on July 14, 2019, at 09:11:04 at Halmahera, Indonesia. Upper panel: Global map showing station-event locations, ray path of the seismic phase, and focal mechanism (colors indicate event depth). Trajectories of the ocean glider and zoomed-in views at the moment of the record in latitude-longitude and depth-longitude plots. Lower panel: wide-window waveform filtered (black and grey lines are waveforms filtered below and above 3 Hz, respectively, and the amplitude is presented in digital units) and its spectrogram. Vertical dashed lines (red/white) and the letter indicate the theoretical arrival time estimated using the ak135f model. Spectral analysis using five frequency bands centered on the theoretical wave arrival (between -30 and 30 seconds), waveforms, and STA/LTA ratio curves for each band and the raw data. The red dot indicates the estimated phase arrival based on SNR > 1.5 and STA/LTA ratio > 3 in the last two frequency bands. The dashed oranges horizontal lines represent the preset lower (1.5) and upper (3) limits for the STA/LTA ratio trigger, and the orange dot is the maximum value.

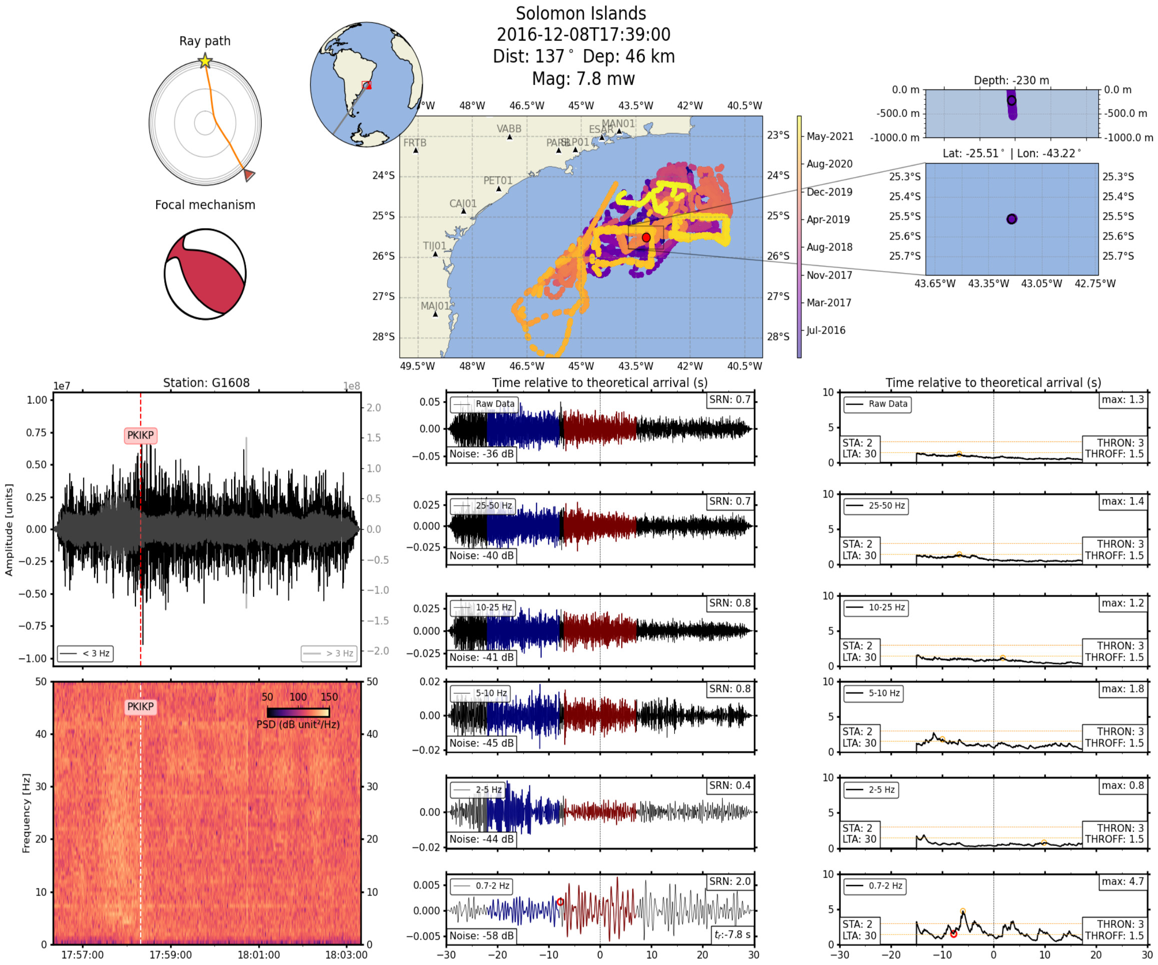

Figure S24: **Waveforms, spectrograms, time series windows centered on the theoretical arrival times, and STA/LTA ratio curves.** Mosaic showing the analysis of the magnitude 7.8 mw earthquake (id: 201612081738A) that occurred on December 08, 2016, at 17:39:00 at Solomon Islands. Upper panel: Global map showing station-event locations, ray path of the seismic phase, and focal mechanism (colors indicate event depth). Trajectories of the ocean glider and zoomed-in views at the moment of the record in latitude-longitude and depth-longitude plots. Lower panel: wide-window waveform filtered (black and grey lines are waveforms filtered below and above 3 Hz, respectively, and the amplitude is presented in digital units) and its spectrogram. Vertical dashed lines (red/white) and the letter indicate the theoretical arrival time estimated using the ak135f model. Spectral analysis using five frequency bands centered on the theoretical wave arrival (between -30 and 30 seconds), waveforms, and STA/LTA ratio curves for each band and the raw data. The red dot indicates the estimated phase arrival based on SNR > 1.5 and STA/LTA ratio > 3 in the last two frequency bands. The dashed oranges horizontal lines represent the preset lower (1.5) and upper (3) limits for the STA/LTA ratio trigger, and the orange dot is the maximum value.

Detection performance between glider and inland stations

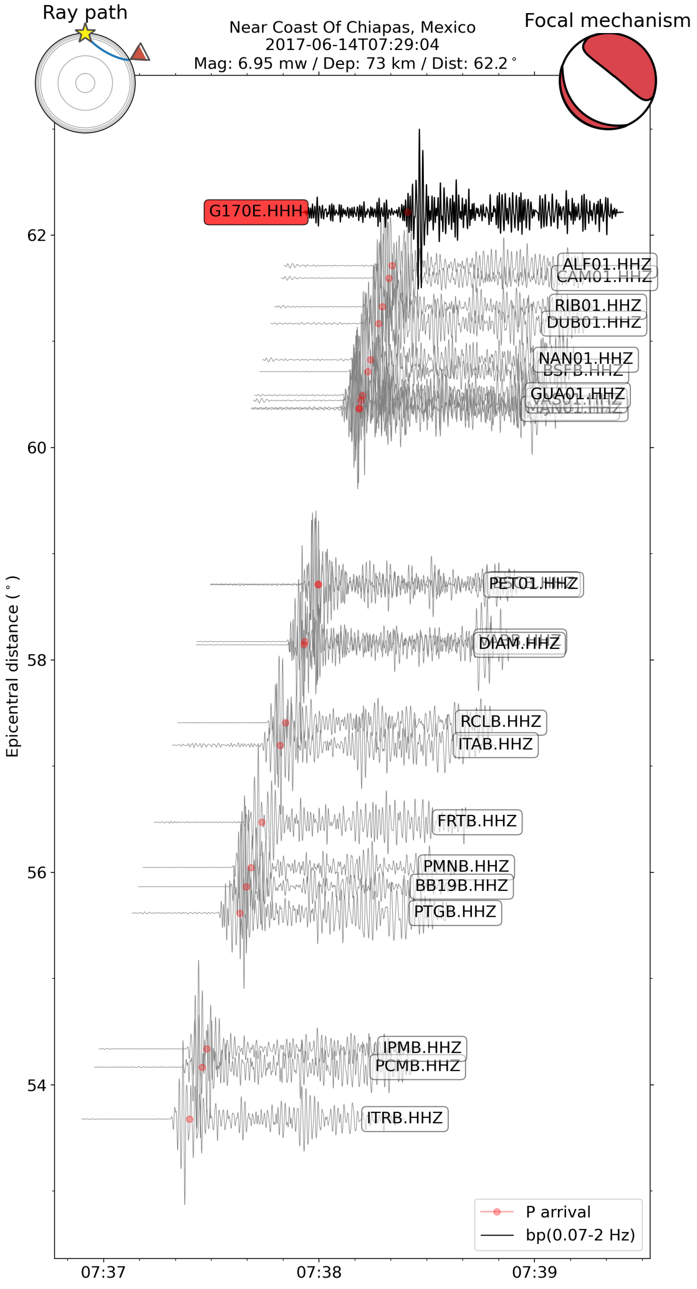

Figure S25: **Comparison of waveforms between ocean gliders and RSBR inland stations.** Comparison of waveforms from the glider (black line) and the Brazilian Seismographic Network stations (grey lines) for the magnitude 6.95 mw earthquake (id: 201706140729A) on June 14, 2017, at 07:29:07 UTC at Near Coast Of Chiapas, Mexico. The waveforms were filtered using a bandpass filter between 0.7 and 2 Hz. Theoretical travel times of P waves according to the ak135f model (land station) and modified ak135f model (glider) are indicated by red circles.

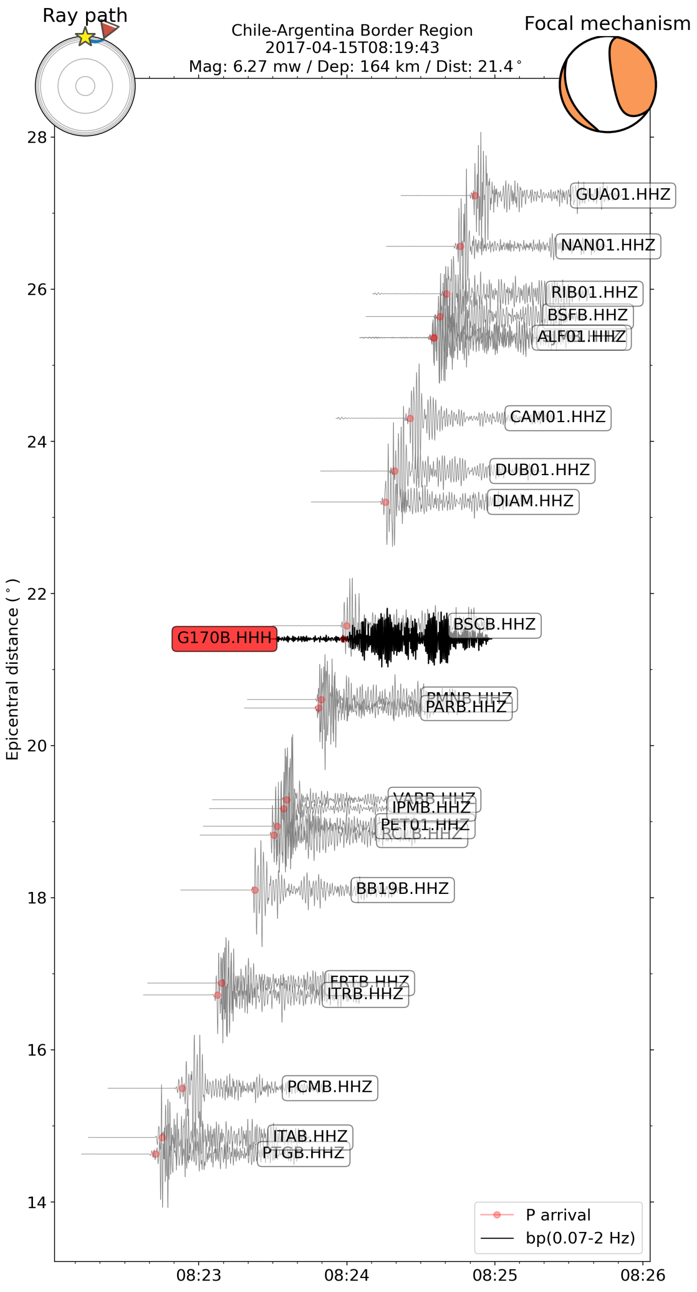

Figure S26: **Comparison of waveforms between ocean gliders and RSBR inland stations.** Comparison of waveforms from the glider (black line) and the Brazilian Seismographic Network stations (grey lines) for the magnitude 6.27 mw earthquake (id: 201704150819A) on April 15, 2017, at 08:19:48 UTC at Chile-Argentina Border Region. The waveforms were filtered using a bandpass filter between 0.7 and 2 Hz. Theoretical travel times of P waves according to the ak135f model (land station) and modified ak135f model (glider) are indicated by red circles.

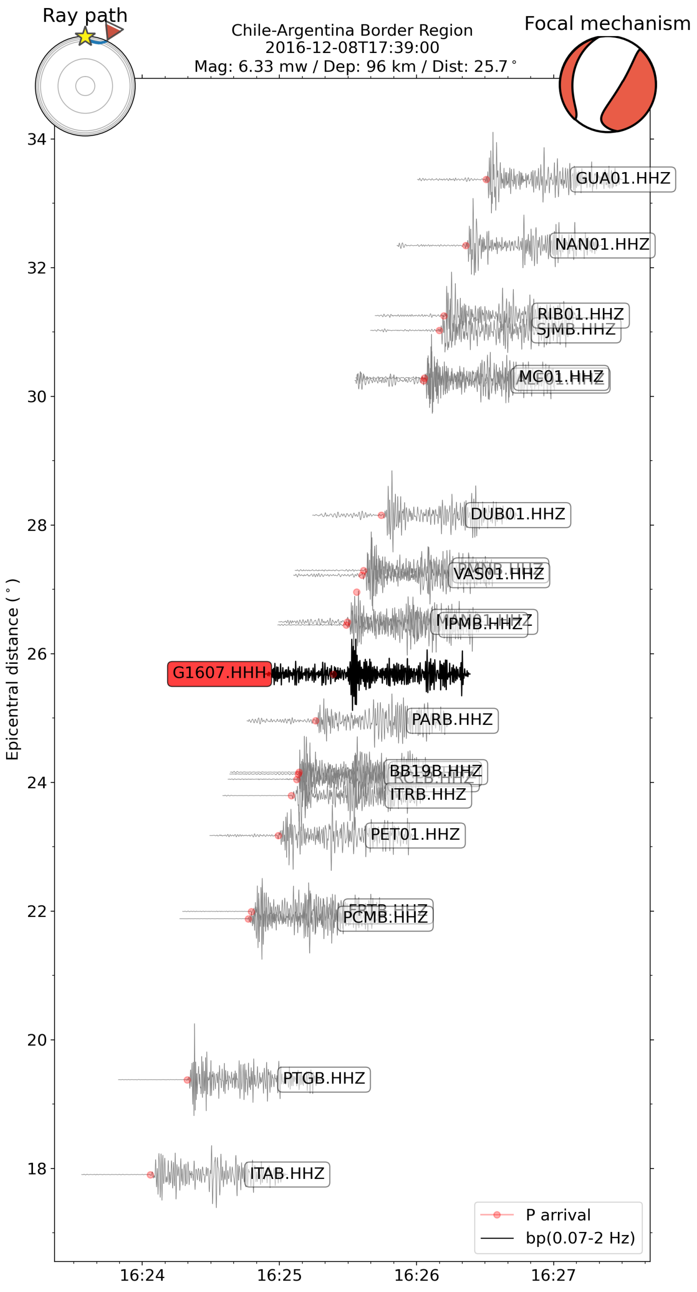

Figure S27: **Comparison of waveforms between ocean gliders and RSBR inland stations.** Comparison of waveforms from the glider (black line) and the Brazilian Seismographic Network stations (grey lines) for the magnitude 6.33 mw earthquake (id: 201611041620A) on November 04, 2016, at 16:20:49 UTC at Central Chile. The waveforms were filtered using a bandpass filter between 0.7 and 2 Hz. Theoretical travel times of P waves according to the ak135f model (land station) and modified ak135f model (glider) are indicated by red circles.

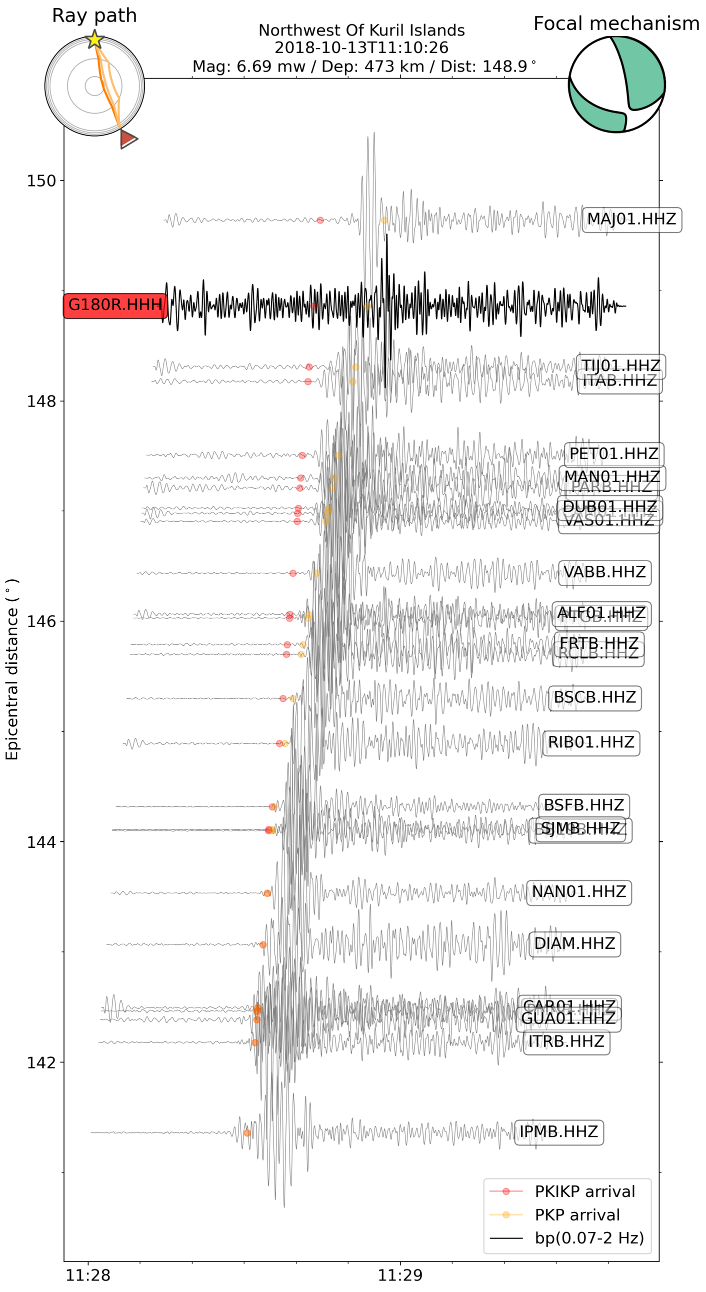

Figure S28: **Comparison of waveforms between ocean gliders and RSBR inland stations.** Comparison of waveforms from the glider (black line) and the Brazilian Seismographic Network stations (grey lines) \ for the magnitude 6.69 mw earthquake (id: 201810131110A) on October 13, 2018, at 11:10:26 UTC at Northwest Of Kuril Islands. The waveforms were filtered using a bandpass filter between 0.7 and 2 Hz. Theoretical travel times of P waves according to the ak135f model (land station) and modified ak135f model (glider) are indicated by red circles (PKIKP) and orange circles (PKP).

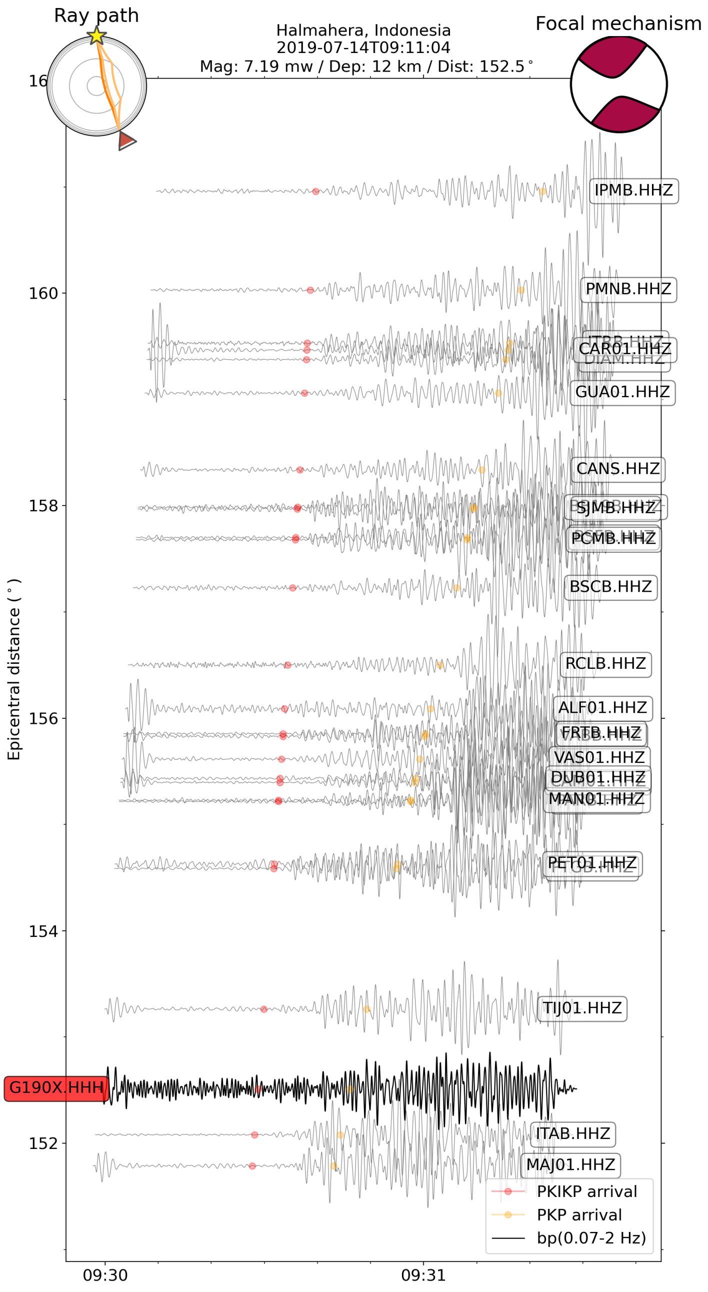

Figure S29: **Comparison of waveforms between ocean gliders and RSBR inland stations.** Comparison of waveforms from the glider (black line) and the Brazilian Seismographic Network stations (grey lines) \ for the magnitude 7.19 mw earthquake (id: 201907140910A) on July 14, 2019, at 09:11:04 UTC at Halmahera, Indonesia. The waveforms were filtered using a bandpass filter between 0.7 and 2 Hz. Theoretical travel times of P waves according to the ak135f model (land station) and modified ak135f model (glider) are indicated by red circles (PKIKP) and orange circles (PKP).

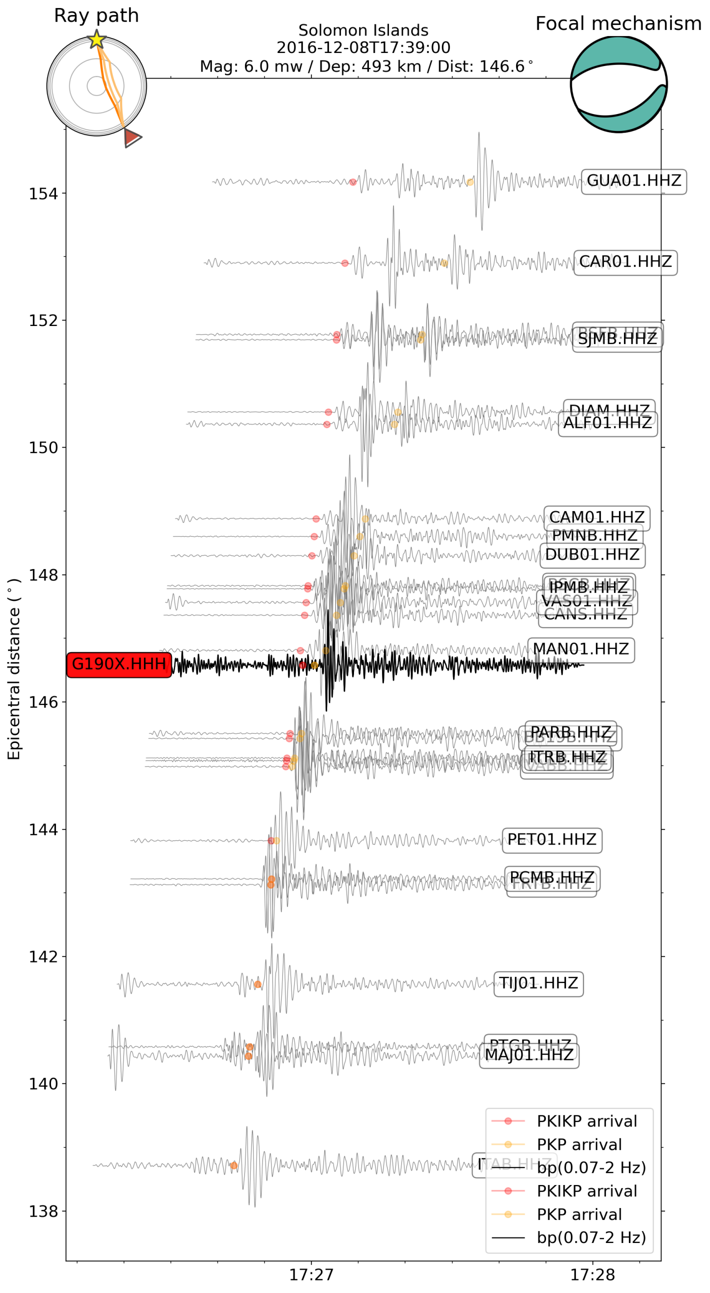

Figure S30: **Comparison of waveforms between ocean gliders and RSBR inland stations.** Comparison of waveforms from the glider (black line) and the Brazilian Seismographic Network stations (grey lines) \ for the magnitude 6.0 mw earthquake (id: 201907111708A) on July 11, 2019, at 17:08:38 UTC at Solomon Islands. The waveforms were filtered using a bandpass filter between 0.7 and 2 Hz. Theoretical travel times of P waves according to the ak135f model (land station) and modified ak135f model (glider) are indicated by red circles (PKIKP) and orange circles (PKP).

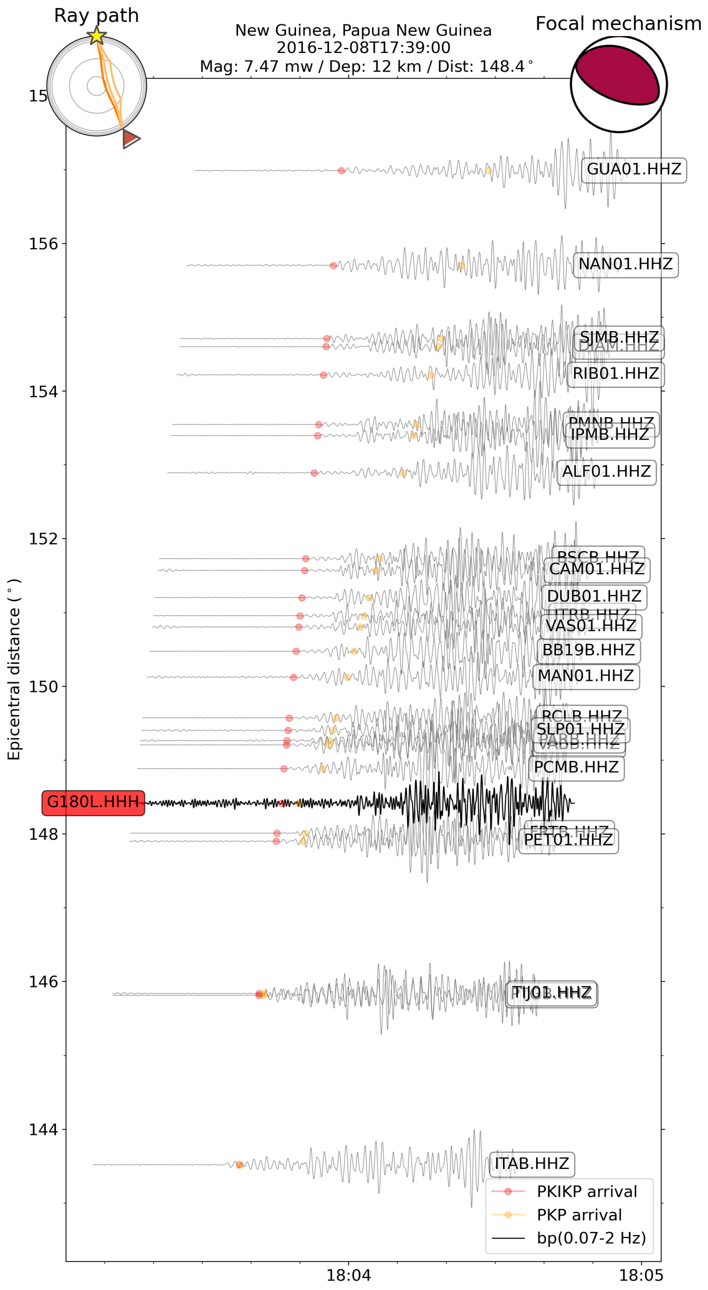

Figure S31: **Comparison of waveforms between ocean gliders and RSBR inland stations.** Comparison of waveforms from the glider (black line) and the Brazilian Seismographic Network stations (grey lines) \ for the magnitude 7.47 mw earthquake (id: 201802251744A) on February 25, 2018, at 17:45:08 UTC at New Guinea, Papua New Guinea. The waveforms were filtered using a bandpass filter between 0.7 and 2 Hz. Theoretical travel times of P waves according to the ak135f model (land station) and modified ak135f model (glider) are indicated by red circles (PKIKP) and orange circles (PKP).

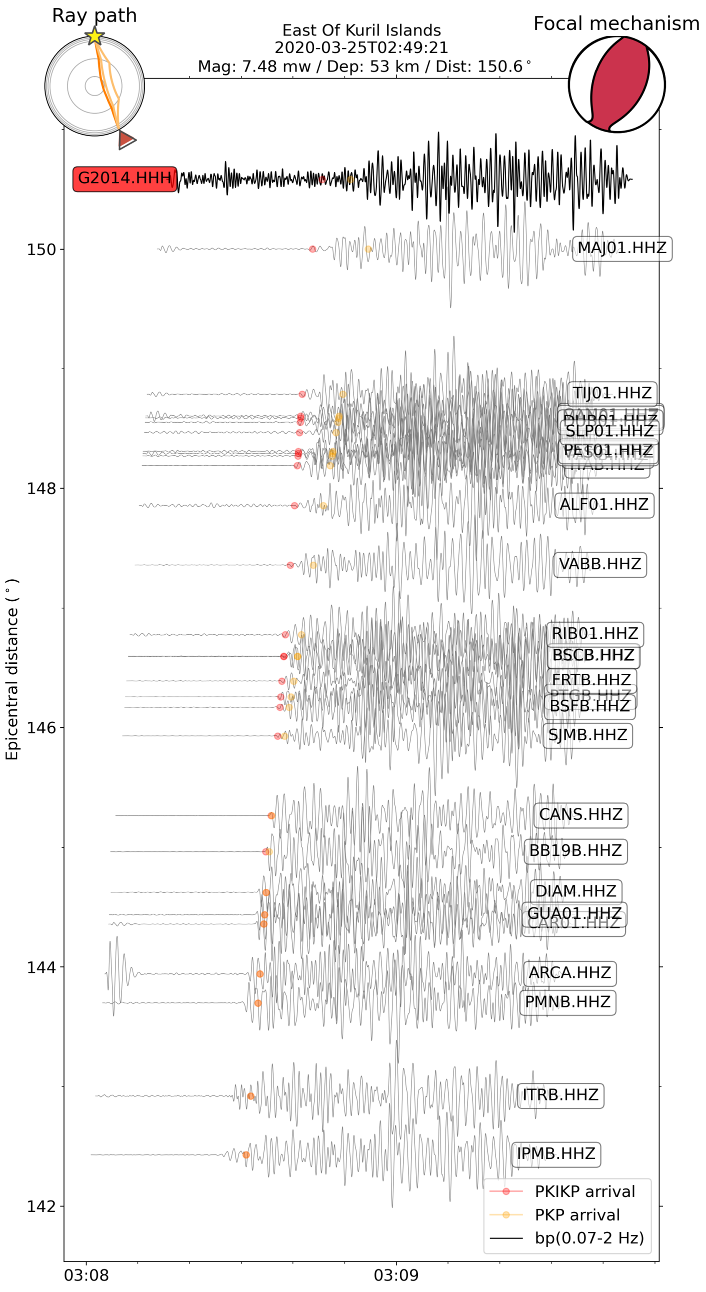

Figure S32: **Comparison of waveforms between ocean gliders and RSBR inland stations.** Comparison of waveforms from the glider (black line) and the Brazilian Seismographic Network stations (grey lines) \ for the magnitude 7.48 mw earthquake (id: 202003250249A) on March 25, 2020, at 02:49:32 UTC at East Of Kuril Islands. The waveforms were filtered using a bandpass filter between 0.7 and 2 Hz. Theoretical travel times of P waves according to the ak135f model (land station) and modified ak135f model (glider) are indicated by red circles (PKIKP) and orange circles (PKP).

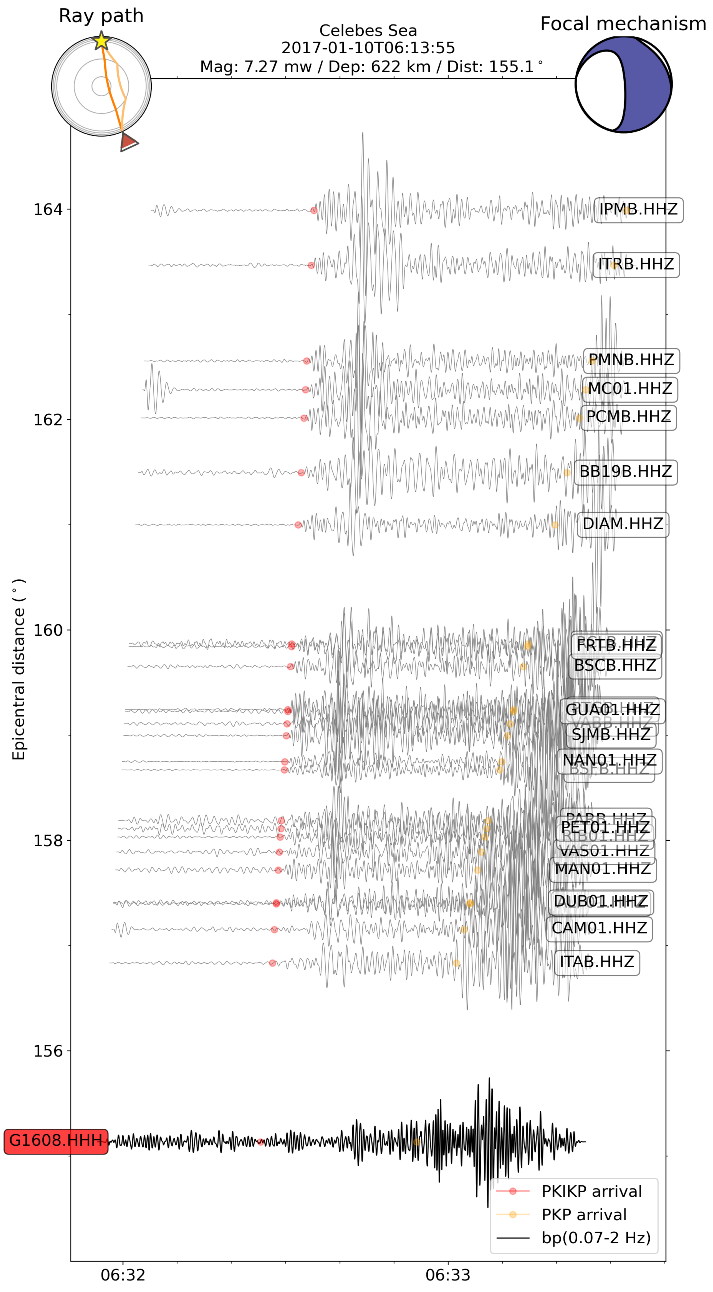

Figure S33: **Comparison of waveforms between ocean gliders and RSBR inland stations.** Comparison of waveforms from the glider (black line) and the Brazilian Seismographic Network stations (grey lines) \ for the magnitude 7.27 mw earthquake (id: 201701100613A) on January 10, 2017, at 06:13:55 UTC at Celebes Sea. The waveforms were filtered using a bandpass filter between 0.7 and 2 Hz. Theoretical travel times of P waves according to the ak135f model (land station) and modified ak135f model (glider) are indicated by red circles (PKIKP) and orange circles (PKP).

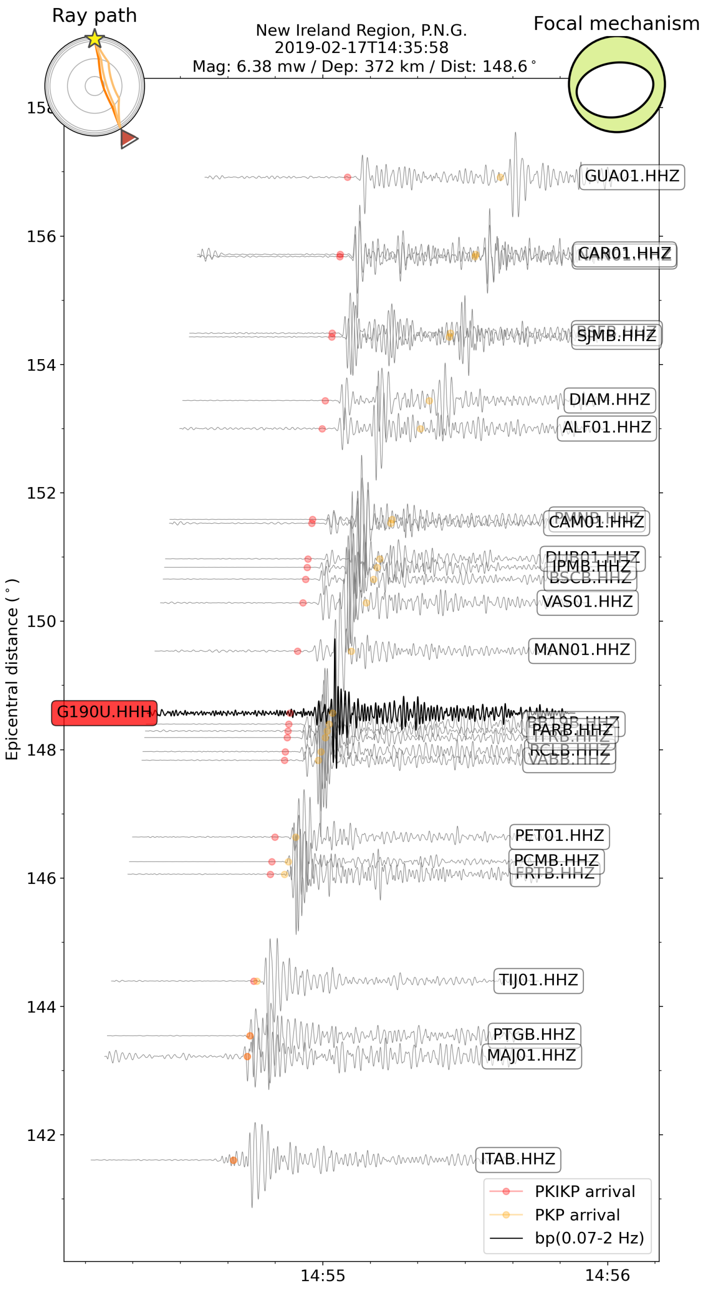

Figure S34: **Comparison of waveforms between ocean gliders and RSBR inland stations.** Comparison of waveforms from the glider (black line) and the Brazilian Seismographic Network stations (grey lines) \ for the magnitude 6.38 mw earthquake (id: 201902171435A) on February 17, 2019, at 14:35:58 UTC at New Ireland Region, P.N.G.. The waveforms were filtered using a bandpass filter between 0.7 and 2 Hz. Theoretical travel times of P waves according to the ak135f model (land station) and modified ak135f model (glider) are indicated by red circles (PKIKP) and orange circles (PKP).

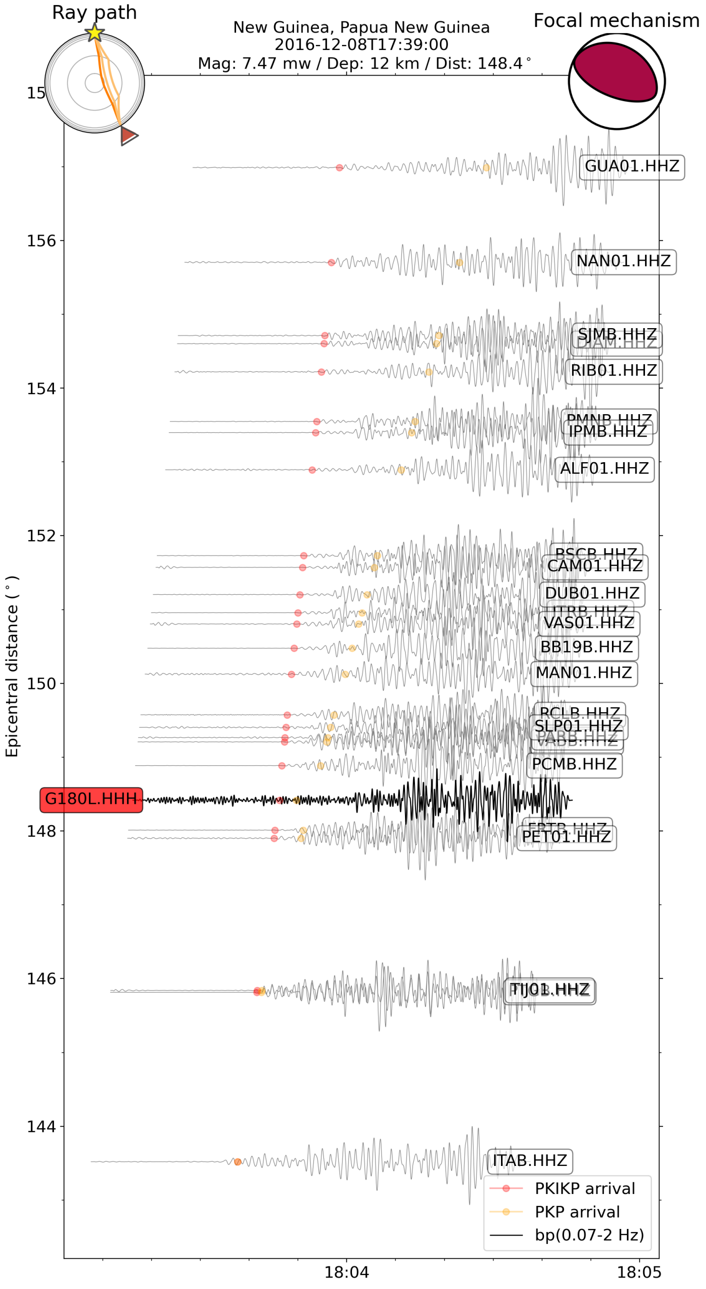

Figure S35: **Comparison of waveforms between ocean gliders and RSBR inland stations.** Comparison of waveforms from the glider (black line) and the Brazilian Seismographic Network stations (grey lines) \ for the magnitude 7.47 mw earthquake (id: 201802251744A) on February 25, 2018, at 17:45:08 UTC at New Guinea, Papua New Guinea. The waveforms were filtered using a bandpass filter between 0.7 and 2 Hz. Theoretical travel times of P waves according to the ak135f model (land station) and modified ak135f model (glider) are indicated by red circles (PKIKP) and orange circles (PKP).

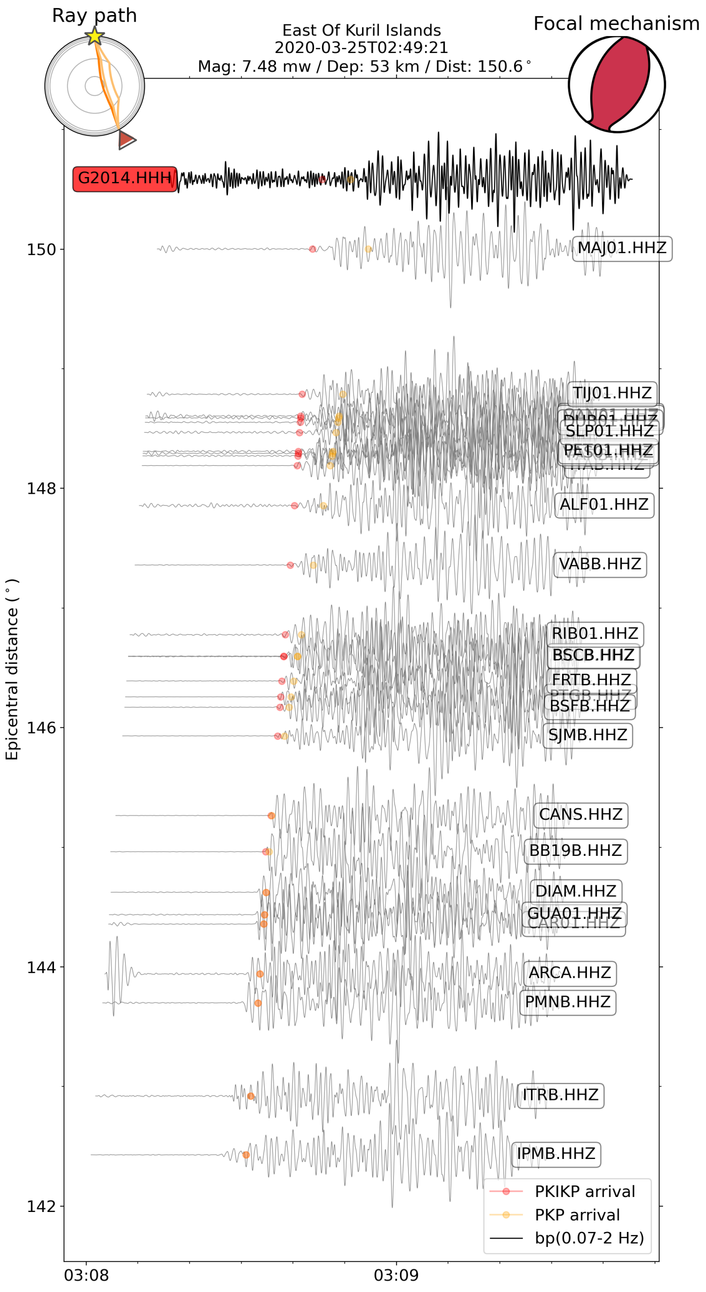

Figure S36: **Comparison of waveforms between ocean gliders and RSBR inland stations.** Comparison of waveforms from the glider (black line) and the Brazilian Seismographic Network stations (grey lines) \ for the magnitude 7.48 mw earthquake (id: 202003250249A) on March 25, 2020, at 02:49:32 UTC at East Of Kuril Islands. The waveforms were filtered using a bandpass filter between 0.7 and 2 Hz. Theoretical travel times of P waves according to the ak135f model (land station) and modified ak135f model (glider) are indicated by red circles (PKIKP) and orange circles (PKP).

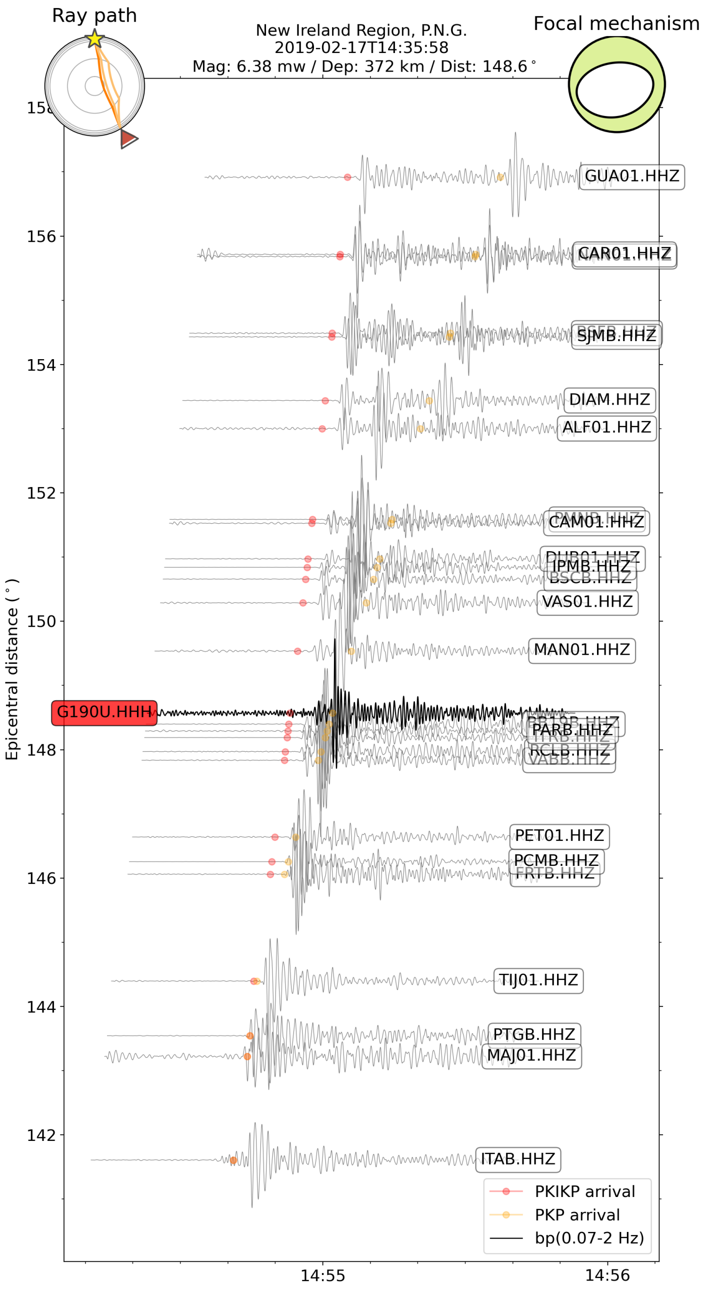

Figure S37: **Comparison of waveforms between ocean gliders and RSBR inland stations.** Comparison of waveforms from the glider (black line) and the Brazilian Seismographic Network stations (grey lines) \ for the magnitude 6.38 mw earthquake (id: 201902171435A) on February 17, 2019, at 14:35:58 UTC at New Ireland Region, P.N.G.. The waveforms were filtered using a bandpass filter between 0.7 and 2 Hz. Theoretical travel times of P waves according to the ak135f model (land station) and modified ak135f model (glider) are indicated by red circles (PKIKP) and orange circles (PKP).

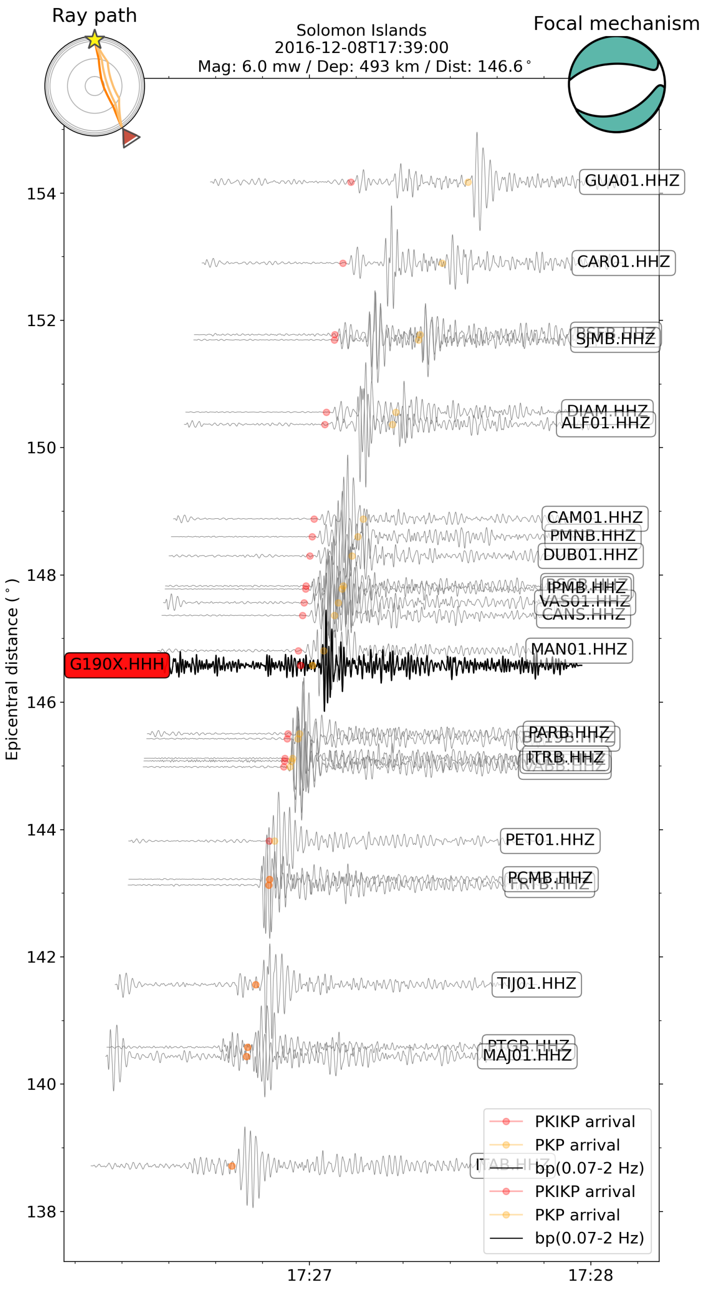

Figure S38: **Comparison of waveforms between ocean gliders and RSBR inland stations.** Comparison of waveforms from the glider (black line) and the Brazilian Seismographic Network stations (grey lines) \ for the magnitude 6.0 mw earthquake (id: 201907111708A) on July 11, 2019, at 17:08:38 UTC at Solomon Islands. The waveforms were filtered using a bandpass filter between 0.7 and 2 Hz. Theoretical travel times of P waves according to the ak135f model (land station) and modified ak135f model (glider) are indicated by red circles (PKIKP) and orange circles (PKP).

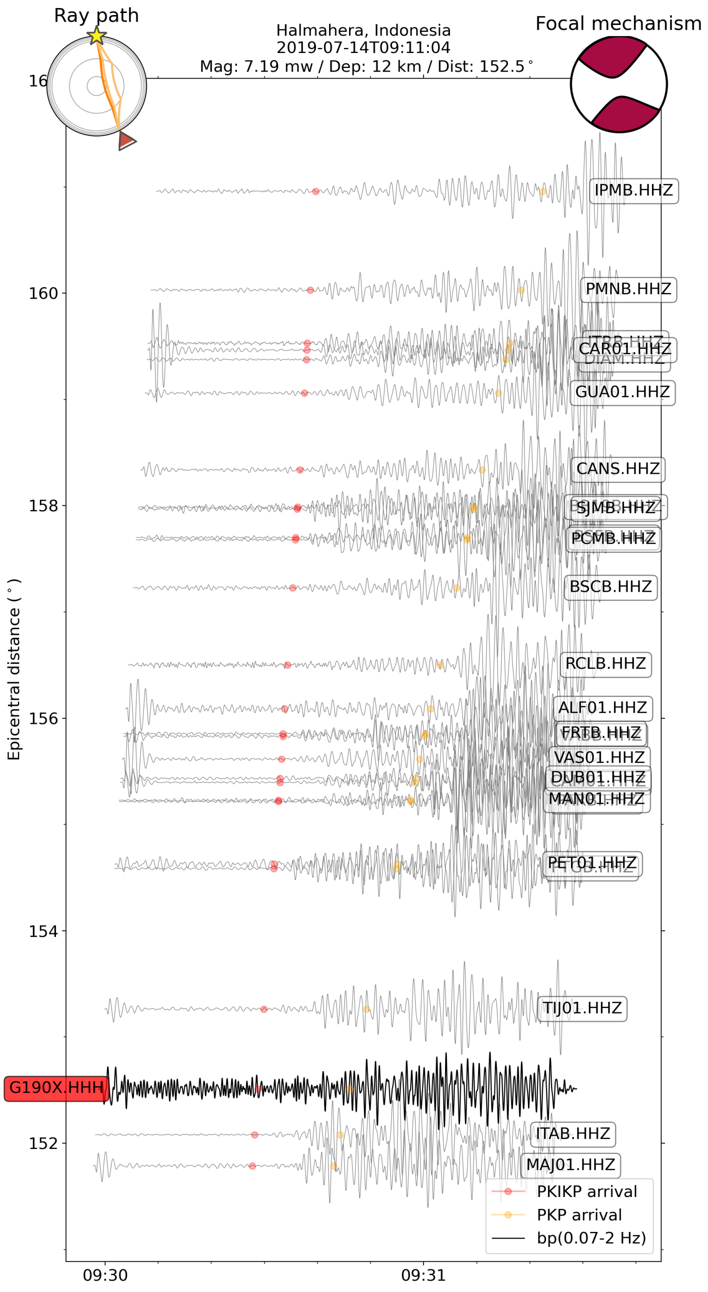

Figure S39: **Comparison of waveforms between ocean gliders and RSBR inland stations.** Comparison of waveforms from the glider (black line) and the Brazilian Seismographic Network stations (grey lines) \ for the magnitude 7.19 mw earthquake (id: 201907140910A) on July 14, 2019, at 09:11:04 UTC at Halmahera, Indonesia. The waveforms were filtered using a bandpass filter between 0.7 and 2 Hz. Theoretical travel times of P waves according to the ak135f model (land station) and modified ak135f model (glider) are indicated by red circles (PKIKP) and orange circles (PKP).

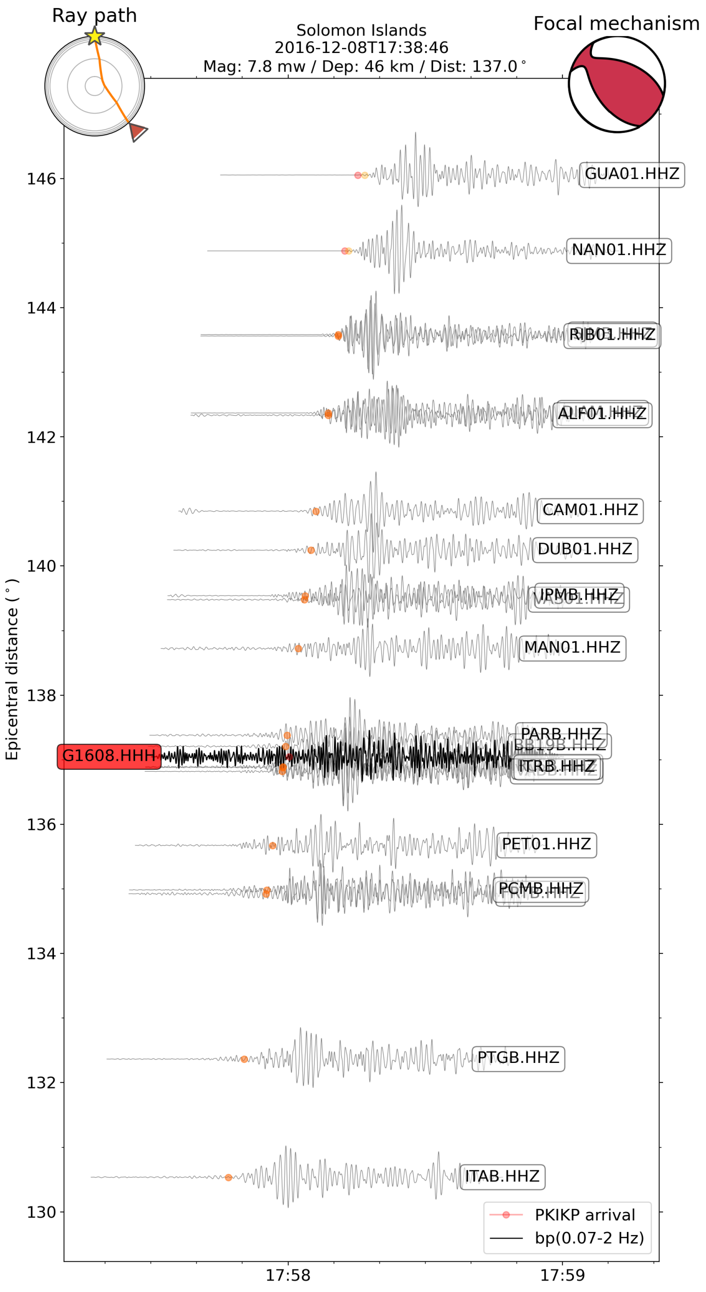

Figure S40: **Comparison of waveforms between ocean gliders and RSBR inland stations.** Comparison of waveforms from the glider (black line) and the Brazilian Seismographic Network stations (grey lines) \ for the magnitude 7.8 mw earthquake (id: 201612081738A) on December 08, 2016, at 17:39:00 UTC at Solomon Islands. The waveforms were filtered using a bandpass filter between 0.7 and 2 Hz. Theoretical travel times of P waves according to the ak135f model (land station) and modified ak135f model (glider) are indicated by red circles (PKIKP) and orange circles (PKP).

Local earthquake automatically detected

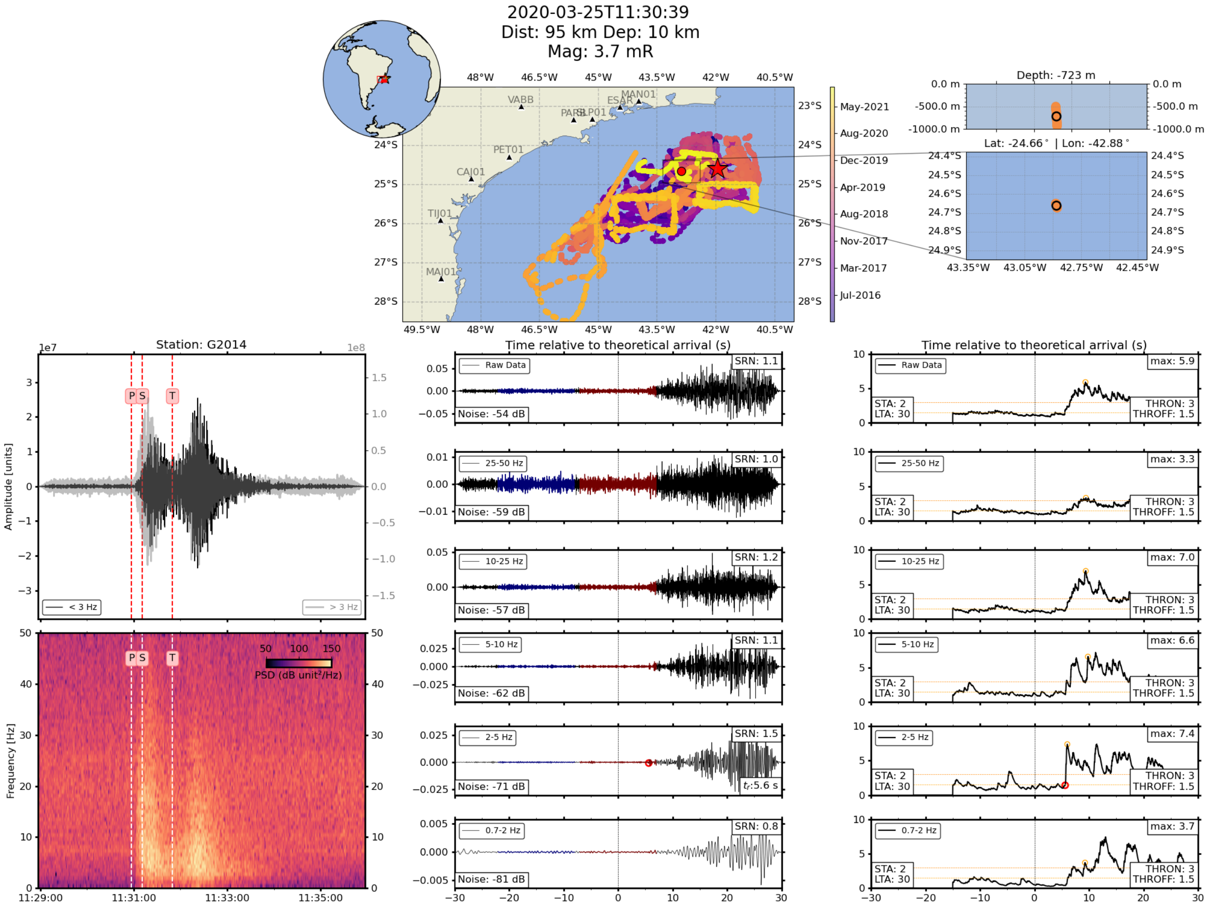

Figure S41: **Waveforms, spectrograms, time series windows centered on the theoretical arrival times, and STA/LTA ratio curves.** Mosaic showing the analysis of the magnitude 3.7 mR earthquake that occurred on March 25, 2020, at 11:30:39 UTC at Brazilian Continental Margin. Global map showing station-event locations and trajectories of the ocean glider, with zoomed-in views of the record moment in latitude-longitude and depth-longitude plots. Lower panel: wide-window waveform filtered (black and grey lines are waveforms filtered below and above 3 Hz, respectively, and the amplitude is presented in digital units) and its spectrogram. Vertical dashed lines (red/white) and the letter indicate the theoretical arrival time estimated using the modified ak135f model. Spectral analysis using five frequency bands centered on the theoretical wave arrival (between -30 and 30 seconds), waveforms, and STA/LTA ratio curves for each band and the raw data. The red dot indicates the estimated phase arrival based on SNR > 1.5 and STA/LTA ratio > 3 in the last two frequency bands. The dashed orange horizontal lines represent the preset lower (1.5) and upper (3) limits for the STA/LTA ratio trigger, and the orange dot is the maximum value.

Table S1: Summary of global earthquakes recorded between November 2015 and November 2021

Table S1: **Summary of global earthquakes recorded between November 2015 and November 2021.** The earthquakes are classified according to depth (H) — shallow (S), intermediate (I), and deep (D) — and focal mechanism (FM) — dip-slip (DS) and strike-slip fault (SS). The data is sorted by epicentral distance (Δ) from the ocean glider.

ID (CMT)	Phase	Mag (mw)	H (km)	Clas	Δ (°)	SNR	STA/LTA	Res (s)	FM
201704150819A	P	6.27	164.20	I	21.40	4.70	8.30	-3.42	DS
201611041620A	P	6.33	96.20	I	25.69	1.90	7.80	1.87	DS
201706140729A	P	6.95	72.70	I	62.22	3.70	9.24	-3.80	DS
201612081738A	PKIKP	7.80	45.50	S	137.05	2.00	4.72	-7.75	DS
201907111708A	PKP	6.00	493.10	D	146.58	3.20	9.29	1.32	DS
201907111708A	PKIKP	6.00	493.10	D	146.58	3.10	9.24	3.82	DS
201802251744A	PKP	7.47	12.00	S	148.42	3.00	6.02	-3.49	DS
201802251744A	PKIKP	7.47	12.00	S	148.42	3.00	5.97	0.01	DS
201902171435A	PKP	6.38	372.20	D	148.57	7.50	11.42	-2.27	DS
201902171435A	PKIKP	6.38	372.20	D	148.57	5.30	11.43	1.83	DS
201810131110A	PKP	6.69	473.40	D	148.86	1.70	6.44	2.11	DS
202003250249A	PKP	7.48	52.60	S	150.58	3.20	5.04	-7.65	DS
202003250249A	PKIKP	7.48	52.60	S	150.58	2.60	5.15	-2.16	DS
201907140910A	PKP	7.19	12.00	S	152.51	1.90	5.36	-0.24	SS
201907140910A	PKIKP	7.19	12.00	S	152.51	1.60	3.53	6.65	SS
201701100613A	PKP	7.27	621.50	D	155.14	2.40	6.83	2.39	DS
